# Single‐Step Synthesis of Mesoporous Vinyl Polymers via Hierarchical Assembly of Stereocontrolled Chains and Their Unique Properties

**DOI:** 10.1002/smll.202509954

**Published:** 2025-11-09

**Authors:** Su Hwa Kim, Jin Jo, Tae Won Park, Young Mee Jung, Hyun Hoon Song, Sang Uck Lee, Yeonju Park, Won Kyung Moon, Eunji Lee, Gyu Weon Hwang, Min Kwan Kang, Min Hwan Jung, Won Gi Hong, Daewon Sohn, Youngil Lee, Kyung Byung Yoon, Seung Hoon Shin, Hee‐Sun Han, Yang‐Kyoo Han

**Affiliations:** ^1^ Department of Chemistry Hanyang University Seongdong‐gu Seoul 04763 Republic of Korea; ^2^ Department of Chemistry Kangwon National University Chuncheon‐si Gangwon‐do 24341 Republic of Korea; ^3^ Department of Advanced Materials Hannam University Daedeok‐gu Daejeon 34430 Republic of Korea; ^4^ School of Chemical Engineering Sungkyunkwan University Jangan‐gu Suwon 16419 Republic of Korea; ^5^ Department of Chemistry Sogang University Mapo‐gu Seoul 04107 Republic of Korea; ^6^ School of Materials Science and Engineering Gwangju Institute of Science and Technology Buk‐gu Gwangju 61005 Republic of Korea; ^7^ Center for Semiconductor Technology Korea Institute of Science and Technology Seongbuk‐gu Seoul 02792 Republic of Korea; ^8^ LG Chem Yuseong‐gu Daejeon 34122 Republic of Korea; ^9^ Research Center for Materials Analysis Korea Basic Science Institute Yuseong‐gu Daejeon 34133 Republic of Korea; ^10^ Department of Chemistry University of Ulsan Nam‐gu Ulsan 44610 Republic of Korea; ^11^ Department of Chemistry University of Illinois at Urbana‐Champaign 600 S. Mathews Avenue Urbana IL 61801 USA

**Keywords:** blue emission, hierarchical assembly, mesoporous materials, vinyl polymers, VOC absorption

## Abstract

Mesoporous materials, vital in energy, environmental, and medical applications, require resource‐intensive production. Mesoporous vinyl polymers, p‐alkyl‐N‐phenyl‐acrylamide (APAA) polymers is introduced, which offer unprecedented advantages in mass production. APAA monomers swiftly form syndiotactic chains through Monomer Aggregation‐mediated Rapid, Radical Stereocontrolled (MARRS) polymerization, spontaneously creating mesoporous structures. Structural analyses (X‐ray, NMR, DSC, FTIR, TEM, and Brunauer‐Emmett‐Teller measurements) and molecular mechanics simulations suggest the formation of Y‐shaped clusters via intermolecular hydrogen bonding between the syndiotactic chains, which then form either a lamellar or hexagonal cylindrical structure containing mesopores. APAA polymers are highly processable, enabling the straightforward production of microfibers, films, and microparticles. They exhibit significant blue light emission (Quantum Yield: 7–18%) while maintaining exceptional transparency in the visible range. UV‐crosslinked APAA polymer fibers, which have both superhydrophobicity and strong water adhesion (petal effect), along with crosslinked APAA polymer microparticles, are highly effective at absorbing liquid‐phase volatile organic compounds (VOCs) such as chloroform, tetrahydrofuran, benzene, and toluene. They achieve both rapid absorption (<10 s) and high absorption capacity. Their remarkably simple and economical synthesis, together with their unique physicochemical properties, position APAA polymers as promising, commercially sustainable mesoporous materials with diverse applications such as UV absorbers, transparent blue‐emitting films, anti‐counterfeiting materials, and water remediation.

## Graphical abstract

This study presents a new class of mesoporous materials—vinyl‐based APAA polymers—offering an efficient, low‐cost alternative to conventional methods. Through a novel MARRS polymerization process, these polymers self‐assemble into structured mesopores and display unique properties like UV absorption, blue light emission, VOC absorption, and water repellency, opening up practical applications in optics, filtration, and sustainability.

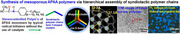

## 1 Introduction

Porous materials^[^ ^1^, ^2^ ^]^ are essential in diverse applications, including molecular separation,^[^ ^3^ ^]^ environmental remediation,^[^ ^4^ ^]^ heterogeneous catalysis,^[^ ^5^, ^6^ ^]^ energy storage,^[^ ^7^ ^]^ water harvesting,^[^ ^8^ ^]^ and drug delivery.^[^ ^9^, ^10^ ^]^ Micro‐ (< 2 nm) and meso‐ (2–50 nm) porous materials are particularly useful due to high surface‐to‐volume ratios and pore sizes that match the size of target adsorbates. However, the production of mesoporous materials remains lengthy and resource‐intensive, limiting industrial scalability and cost competitiveness.

Mesoporous materials are synthesized by templated synthesis or direct assembly. Templated synthesis utilizes sacrificial porous materials, such as porous silica or organosilica‐based templates^[^ ^11^, ^12^, ^13^ ^]^ or polymer‐based templates.^[^ ^14^, ^15^ ^]^ Despite its widespread use, templated synthesis involves multiple steps of sensitive processes, costly reagents or equipment, and toxic chemicals. Direct assembly generally involves fewer steps and produces well‐defined structures. Simple condensation reactions produce various polymer‐based mesoporous structures^[^ ^4^, ^16^, ^17^ ^]^ such as porous organic polymers, covalent organic frameworks, porous aromatic frameworks, and polymers of intrinsic microporosity. Metal–organic coordination and hydrogen bonding are also used for metal–organic frameworks (MOFs),^[^ ^3^, ^18^ ^]^ polymer‐MOFs,^[^ ^19^ ^]^ and hydrogen‐bonded organic frameworks,^[^ ^20^ ^]^ respectively. Despite their simple procedures, direct assembly methods require complex building blocks, such as di‐/tri‐/tetra‐functional monomers, and specialized catalysts, driving up costs and limiting scalability.

Vinyl polymers, with their simple yet versatile chemistry and exceptional processability, offer unparalleled benefits for mass production and commercialization. In fact, polyethylene/polypropylene/polystyrene (PS)/polyvinyl chloride‐based macroporous foams produced by radical polymerization in high internal phase emulsions^[^ ^21^ ^]^ are widely used as shock‐absorbing materials, insulating materials, and sponge‐type nonwovens. However, there is still no method to produce mesoporous vinyl polymers in a cost‐effective and scalable manner.

Here, we report a low‐cost single‐step synthesis method for mesoporous vinyl polymers using p‐alkyl‐N‐phenyl‐acrylamides (APAA, Figures S1–S3, Supporting Information). APAA homopolymers (Figure S4, Supporting Information) exhibit unique polymerization and self‐assembly characteristics, exceptional processability, and unique physicochemical properties, making them highly promising for commercial applications.

## 2 Results and Discussion

In our pursuit to develop block copolymers for next‐generation nanolithography applications,^[^ ^22^, ^23^, ^24^ ^]^ we introduced a hard–soft block copolymer, poly(p‐dodecyl‐N‐phenyl‐acrylamide)‐poly(methyl methacrylate) (PDOPAM‐PMMA). This polymer spontaneously assembles into either lamellar or hexagonal cylindrical structures spanning over 10 µm without guide templates. This exceptional ordering is attributed to the stereocontrolled polymerization of p‐dodecyl‐N‐phenyl‐acrylamide (DOPAM) monomers and the robust self‐assembly capability of the PDOPAM blocks. Stereocontrolled polymerization was inferred from the high melting temperature of the PDOPAM segment (≈240 °C). To further investigate this phenomenon, we synthesized homopolymers of various crystalline APAA monomers‐DOPAM (C_12_), p‐tetradecyl‐N‐phenyl‐acrylamide (TEPAM, C_14_), and p‐hexadecyl‐N‐phenyl‐acrylamide (HEPAM, C_16_). Detailed characterization of the resulting APAA polymers revealed two novel phenomena: 1) Monomer Aggregation‐mediated Rapid, Radical Stereocontrolled (MARRS) polymerization and 2) spontaneous, hierarchical assembly of mesoporous structures from syndiotactic APAA polymer chains. These phenomena arise from the unique chemical structure of the APAA monomers, which contain an amide bond that promotes intermolecular hydrogen bonding, a benzene ring that enables π–π interactions, and a long linear alkyl chain that contributes dispersion (London) forces through hydrocarbon interactions.

### 2.1 High Speed, Stereocontrolled Radical Polymerization via Ordered Aggregation of APAA Monomers: MARRS Polymerization

Homopolymerization of APAA monomers using a simple radical initiator, benzoyl peroxide (BPO), yielded high molecular weight, syndiotactic APAA polymers, contrary to typical radical polymerization outcomes. The polymerization proceeds extremely rapidly in benzene, achieving >100 kDa molecular weight within 30 min with >50% conversion efficiency. These polymers exhibit low dispersity (Đ: <2.0) and high syndiotacticity (75–95%) despite the absence of catalysts^[^ ^25^, ^26^ ^]^ for coordinated monomer alignment. Rapid polymerization, low Đ, and high syndiotacticity were achieved regardless of monomer choice, monomer concentrations, and solvents, except for the sample (PT‐6) designed to obtain low stereoregularity by using azobisisobutylronitile (AIBN) and long polymerization time (**Table** **1** **;** Table S1, Supporting Information). In stark contrast, polymerization of conventional acrylamide‐based monomers^[^ ^27^ ^]^ is at least one order of magnitude slower under comparable conditions, producing amorphous polymers with low number average molecular weights (M_n_ <20 kDa), stereoirregularity, and a broad Đ (2‐5). On the other hand, replacing BPO with AIBN results in a wider Đ (2–3: PD‐9 to PD‐11, PT‐5 and PH‐1 in Table S1, Supporting Information), as do higher polymerization temperature (80 °C) and longer polymerization time (48 h) (PD‐0 and PT‐7).

**Table 1:** Polymerization conditions and physical properties of representative stereocontrolled mesoporous APAA polymers prepared by the Monomer Aggregation‐mediated Rapid, Radical Stereocontrolled (MARRS) polymerization method

Name^a)^	wt%^b^ ^)^ of monomer	Solvent	Temp [°C]/Time [h]	M_n_	Đ	Conversion [%]	Syndio‐tacticity^c)^ [%]	BET surface area [m^2^ g^−1^]	Mean pore diameter [nm]
PD‐0	20	Benzene	80/48	116900	2.84	86.4	86.4	242.44	7.26
PD‐1	20	Benzene	70/0.5	191100	1.93	60.1	87.6	267.99	6.94
PD‐4	10	Benzene	70/0.5	134900	1.93	60.3	82.5	264.14	6.70
PD‐6	5	Benzene	70/0.5	85900	1.98	30.4	82.5	255.13	6.85
PD‐8	2	Benzene	70/72	56600	1.80	32.0	77.2	264.91	6.88
PD‐14	10	THF	70/0.5	13200	1.55	26.7	85.0	270.45	5.85
PD‐16^d)^	10	Monoglyme	70/0.5	56700	1.74	15.2	79.6	273.99	6.10
PD‐17	10	Dioxane	70/0.5	92200	2.04	44.0	94.0	281.25	6.86
PT‐0	10	Benzene	70/0.5	117200	1.89	53.2	86.6	257.46	7.64
PT‐8	10	THF	70/0.5	12400	1.5	27.0	93.2	268.23	6.12
PH‐0	10	Benzene	70/0.5	128500	2.05	54.7	82.3	238.84	7.07
PH‐2	10	THF	70/0.5	15000	1.46	30.1	85.3	226.28	7.78

^a)^ PD: PolyDOPAM; PT: PolyTEPAM; PH: PolyHEPAM.

^b)^ Monomer concentration versus solvent.

^c)^ Triad tacticity: the ratio of [syndiotactic] to [isotactic] as measured by the relative proportions of racemic (rr) to meso (mm) triads in ^13^C NMR spectra (see Supporting Information for details).

^d)^ THF‐d_8_ was used as solvent for ^13^C NMR except for PD‐16, which used CDCl_3_.

**Figure 1:**
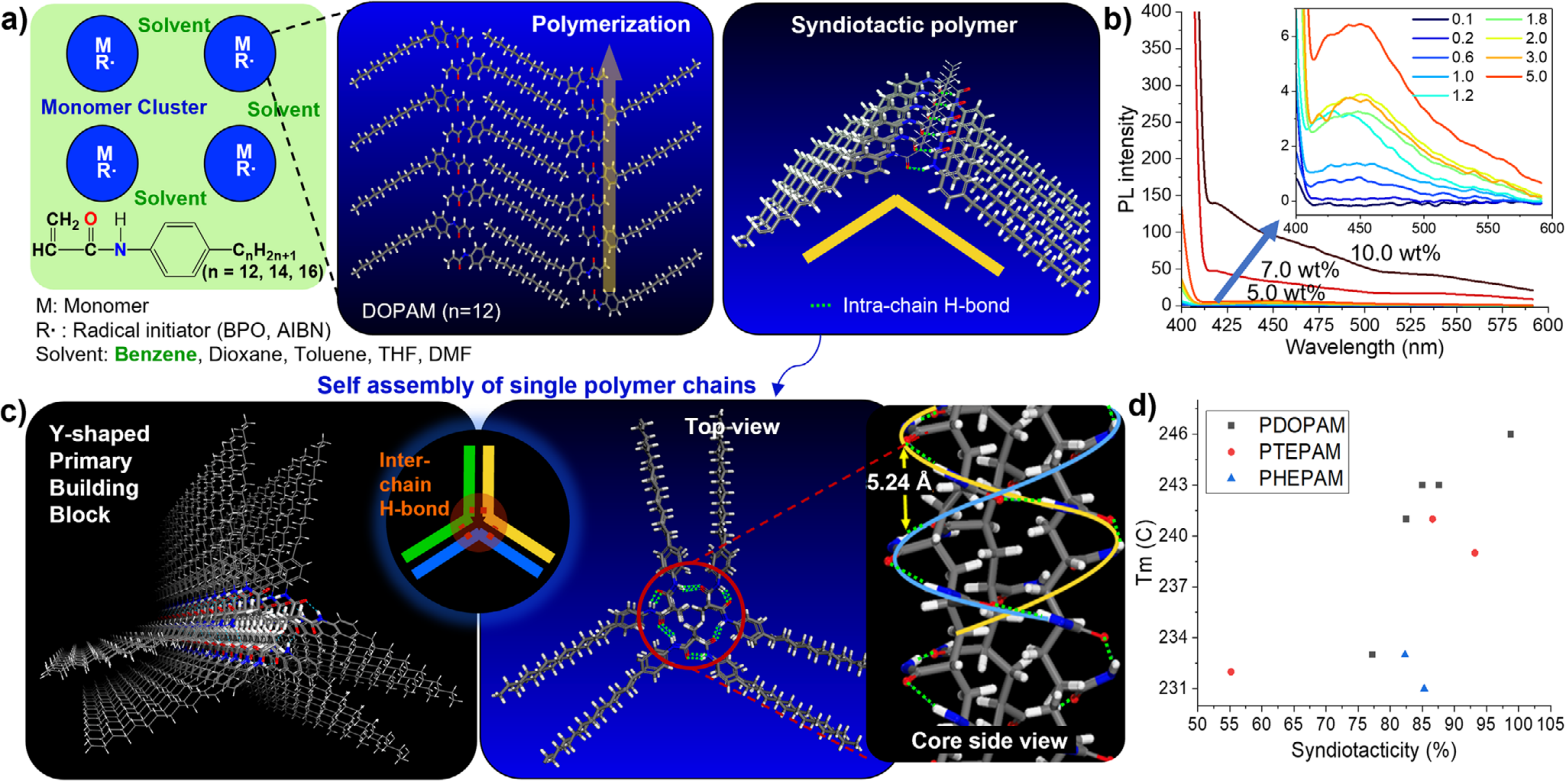
MARRAS polymerization and self‐assembled Y‐clusters. a) Simulated structure of ordered aggregates of APAA monomers. The vertically stacked vinyl groups enable rapid polymerization along the stacking axis, while adjacent amide groups form hydrogen bonds, promoting syndiotactic chain growth; b) Fluorescence spectra of DOPAM in benzene at varying concentrations (λ_ex_ = 365 nm), showing aggregation‐induced emission enhancement; c) Proposed Y‐shaped primary building block (Y‐cluster) composed of three syndiotactic polymer chains. The structure is stabilized by interchain hydrogen bonding and van der Waals interactions among alkyl groups, with intra‐ and interchain hydrogen bonds forming helical networks at the core; d) Melting temperatures of PDOPAM, PTEPAM, and PHEPAM exhibiting strong positive correlations with syndiotacticity, consistent with the proposed assembly model.

These characteristics suggest that APAA monomers undergo ordered aggregation and then polymerize along aligned vinyl groups, a mechanism named MARRS polymerization (**Figure** **1**a). Aggregation‐induced emission enhancement^[^ ^28^ ^]^ in APAA solutions confirms the formation of monomer aggregates. DOPAM solutions in benzene exhibit minimal fluorescence signals up to 5.0 wt.%, but above this concentration, the emission intensity increases substantially, indicating monomer aggregation (Figure 1b; Figure S5, Supporting Information). These aggregates likely mimic the ordered arrangement in DOPAM crystals,^[^ ^24^ ^]^ held together by intermolecular hydrogen bonding, π–π stacking, and hydrocarbon interactions (Figure S6, Supporting Information). Intermonomer hydrogen bonding was confirmed by ^13^C cross‐polarization magic angle spinning (CP‐MAS) solid state NMR spectra: the free carbonyl peak of DOPAM (164.6 ppm in Figure S7, Supporting Information) shifted downfield during polymerization (≈174 ppm in Figures S8–S10 and Table S2, Supporting Information), indicating hydrogen bonding between the amide groups. This organized monomer arrangement would yield high‐speed polymerization along the vertically stacked vinyl groups. Higher monomer concentrations yield DOPAM polymers with higher molecular weights, supporting this model. Specifically, the M_n_ of PDOPAM increases from 56 to 191 kDa when the DOPAM concentration is increased from 2.0 to 20 wt.% (Table 1, PD‐1 > PD‐4 > PD‐6 > PD‐8).

Solvent‐dependent polymerization outcomes further support the ordered aggregation of APAA monomers. The molecular weight of PDOPAM decreases in the order of benzene, toluene, dioxane, monoglyme, and tetrahydrofuran (THF),, which corresponds to an increasing order of solvent dielectric constant. This result suggests that polar ether‐type solvents disrupt monomer aggregation by solvation, thereby slowing polymerization. Benzene produced particularly high molecular weight polymers, likely due to its planar structure, which helps to maintain the ordered monomer arrangement even with intercalation. The high syndiotacticity in all solvents indicates that the monomer orientation is locked into the syndiotactic conformation during incorporation by hydrogen bonding between adjacent monomer units. Syndiotacticity was characterized using ^13^C NMR in solution by analyzing the splitting of the alpha‐carbon peak in the polymer backbone into racemic and meso‐triad peaks (Figures S11–S14, Supporting Information). In contrast to the stereocontrolled APAA polymers, PT‐6 showed neither alpha‐carbon splitting nor splitting of the characteristic carbons in the long alkyl chain, indicating the lack of stereoselectivity (Figures S10 and S13C, Supporting Information).

### 2.2 Self‐Assembly of Syndiotactic APAA Polymer Chains into Y‐Shaped Clusters

PDOPAM samples synthesized under different conditions showed melting temperatures ranging from 233 to 246 °C (Figure S15, Supporting Information). This range is comparable to crystalline syndiotactic polystyrenes (PSs) synthesized using titanium‐based catalysts (240–270 °C),^[^ ^29^, ^30^, ^31^, ^32^ ^]^ supporting the syndiotactic structure identified by NMR. Moreover, the significant variation in melting point despite their high molecular weights (>50 kDa) suggests the presence of self‐assembled suprastructures, as for crystalline PS. Figure 1c presents the thermodynamically stable structure obtained from molecular mechanics simulation, which is consistent with the structural features observed by NMR, IR, XRD, and TEM. Detailed structural characterization presented in this work primarily focused on PD‐14, PT‐8, and PH‐2 because of their high structural regularity stemming from narrow molecular weight distribution (Đ: 1.46‐1.55) and low M_n_ (12–15 kDa). These characteristics minimize branching caused by chain transfer during polymerization.

NMR and IR analyses show that the self‐assembly is driven by interchain hydrogen bonding between the syndiotactic chains. The simulated structure of a syndiotactic polymer chain using SPARTAN‐2008 reveals that half of the carbonyls and amines in the amide groups participate in intramolecular hydrogen bonding, while the other half remains free (Figure S22A,B, Supporting Information). However, both ^13^C CP‐MAS NMR (174 ppm in Figures S8–S10, Supporting Information) and 1D‐IRRAS (3310 cm^−1^, amine;1658 cm^−1^, carbonyl; Figures S16 and S17, Supporting Information) spectra display a single broad peak for carbonyl and amine groups, indicating hydrogen‐bonded amides with no peak in the free amide region. These results suggest that the carbonyl and amine groups, which are not involved in intramolecular hydrogen bonding, engage in intermolecular hydrogen bonding, driving self‐assembly of the polymer chains.

Simulations were performed using nanoscale molecular dynamics (NAMD)^[^ ^33^ ^]^ based on the optimized potentials for liquid simulations (OPLS) force field. The predicted assembly structure indicates that three syndiotactic chains form a Y‐shaped suprastructure (Y‐cluster) through intermolecular hydrogen bonding between free amide groups. These amides form well‐ordered, helical hydrogen‐bonding networks at the core (Figure 1c). This arrangement induces dispersion interactions between the long alkyl chains of adjacent polymer chains. The simulated distance between the helical hydrogen bonding networks is 5.24 Å, and the intra‐ and inter‐chain distances between the benzene moieties are 7.2 and 3.3 Å, respectively (Figure S22C, Supporting Information). Formation of the Y‐cluster requires syndiotactic, linear polymer chains, explaining the positive correlation between syndiotacticity and melting temperature (Figure 1d).

### 2.3 Spontaneous Formation of Mesoporous Structures from Y‐Clusters

**Figure 2:**
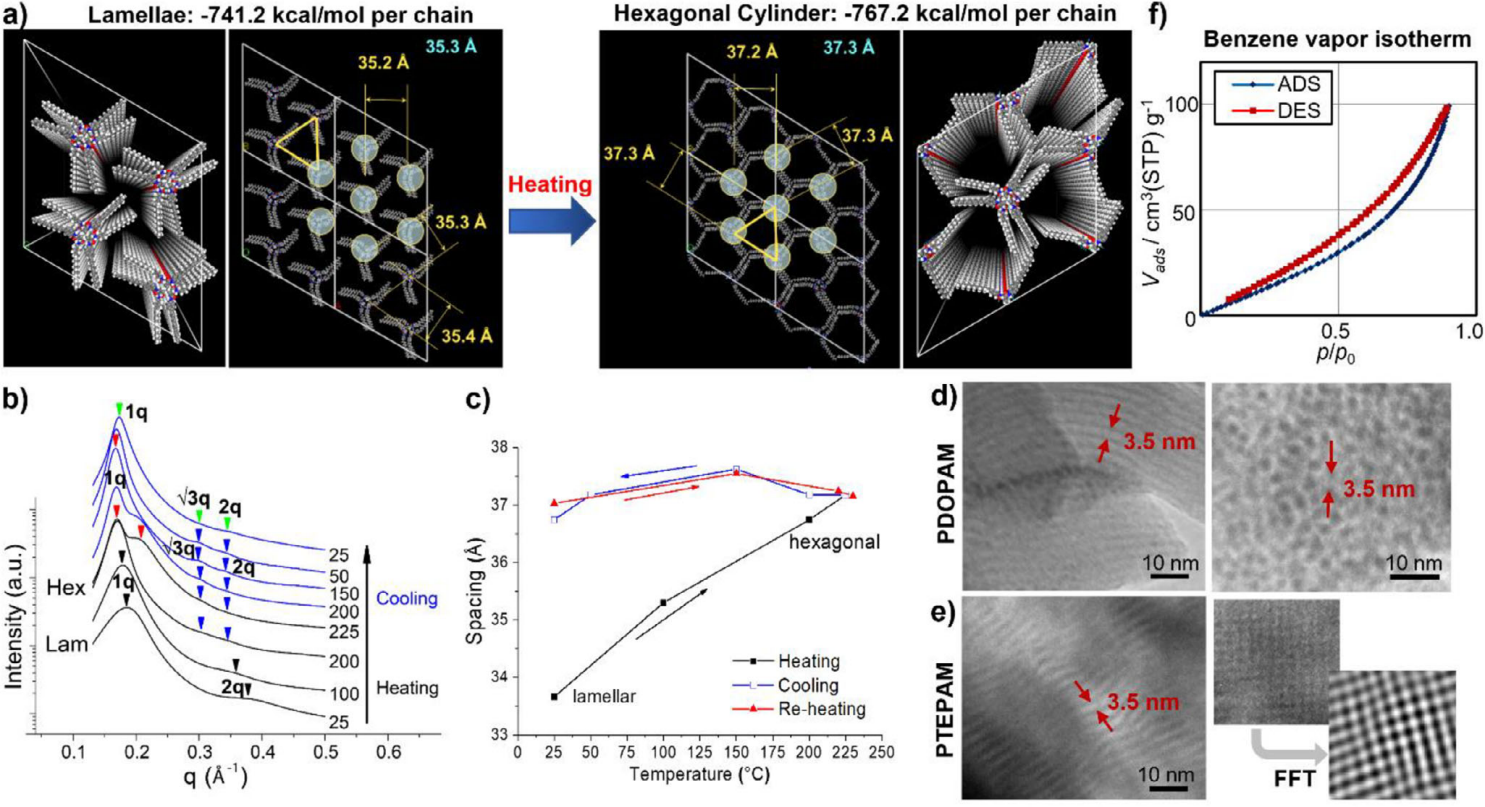
Mesoporous suprastructures formed from Y‐clusters. a) Simulated lamellar and hexagonally packed cylindrical suprastructures and their formation energies. The hexagonal structure contains internal voids that generate mesoporosity; b) Evolution of the SAXS pattern (PD‐14) during heating and cooling cycles, showing a lamellar‐to‐hexagonal‐cylinder transition ≈200 °C. The hexagonal phase remains stable upon cooling; c) Changes in interdomain spacing during thermal cycling. A significant shift accompanies the lamella‐to‐hexagonal transition, while spacing remains constant after the hexagonal structure is formed; d,e) TEM images of ultrathin films of PDOPAM (PD‐14) and PTEPAM (PT‐8). The interdomain spacing between the RuO_4_‐stained dark lines (side view) and the pore‐to‐pore distance (top view) agree with SAXS results. PT‐8 exhibits a typical oblique pattern representing the hexagonally packed cylinders; f) Benzene vapor sorption isotherms of PDOPAM powders (PD‐14).

XRD, TEM, and porosimetry analyses reveal the formation of regularly arranged mesoporous structures with either lamellar or hexagonal ordering, in agreement with the simulation result (**Figure** **2**a). The ability to achieve single‐step hierarchical assembly of mesoporous structures is remarkable, as it enables rapid, large‐scale synthesis of mesoporous materials from simple vinyl monomers without specialized catalysts. We routinely conduct 10 g scale reactions (Figure S4, Supporting Information) and anticipate that larger‐scale synthesis would yield materials of similar quality due to the simplicity of the process.

Wide‐angle X‐ray diffraction (WAXD) and small‐angle X‐ray scattering (SAXS) measurements show that APAA polymers with low Đ and low M_n_ (PD‐14, PT‐8, and PH‐2) form a lamellar structure at room temperature, represented by peaks at 1q, 2q, 3q, and 4q^[^ ^34^ ^]^ (Figure 2b; Figure S18, Supporting Information). Upon thermal annealing above 200 °C, this structure evolves into a hexagonal cylinder structure, represented by peaks at 1q, √3q, 2q, and √7q^[^ ^34^ ^]^ (Figure 2b; Figures S19 and S20, Supporting Information). During this transformation, the interdomain spacing for PD‐14 increases from 33.6 to 37.2 Å (Figure 2c). Once formed, the hexagonal structure remains intact during cooling, indicating its thermodynamic stability. Unlike the lamellar structure, the interdomain spacing of the hexagonal structure shows minimal variation during both cooling and reheating. Additional peaks in WAXD spectra are attributed to pi–pi stacking between benzene groups in the syndiotactic polymer chains (2θ = 19°–19.5°) and hydrophobic chain interactions between long alkyl groups (2θ = 19.7°–21.3°). APAA polymers with high M_n_ (PD‐4, PT‐0, and PH‐0; M_n_ >100 kDa) also form a hexagonal cylinder structure upon annealing at 150 °C (Figure S21, Supporting Information).

Simulation illustrates that the lamellar structure consists of closely packed Y‐clusters with alkyl chains filling the spaces between them. The hexagonal arrangement is formed by rotating the Y‐clusters along the axial axis, entangling the ends of the alkyl chains. As seen in the XRD result, the lamellar‐to‐hexagonal transformation is driven by thermal movement of the alkyl chains because the hexagonal structure accommodates greater movement, reducing resistance to thermal expansion. The hexagonal structure has cylindrical voids surrounded by alkyl chains, suggesting mesoporosity. The predicted intercluster distance for the hexagonal structure is ≈2 Å greater than for the lamellar structure (Figures S22–S25, Supporting Information), matching the distances calculated from the SAXS spectra (Table S3, Supporting Information). The calculated formation energies for the lamellar and hexagonal structures of PDOPAM are −741.2 and −767.2 kcal mol^−1^ per chain, respectively, indicating a higher stability for the hexagonal structure (Figure 2a). Unlike PDOPAM and PTEPAM, PHEPAM is more stable in its lamellar structure (Figure S26, Supporting Information).

TEM measurements confirm the simulated hexagonal arrangement (Figure 1d,e; Figures S27 and S28, Supporting Information). RuO_4_‐stained PDOPAM and PTEPAM samples show regularly spaced dark lines (3.5 and 1.5 nm) corresponding to the Y‐cluster backbones, with spacings consistent with the simulations (Figure S23, Supporting Information) and XRD analysis. Microtome‐sectioned PD‐14 films show regularly arranged dark circles representing individual hexagonal cylinders, with spacing matching the predicted pattern. The typical oblique pattern indicating hexagonal packing of cylindrical structures is also observed in PT‐8. In contrast, PHEPAM shows no obvious pattern, suggesting that its longer alkyl chains prevent ordered alignment due to increased movement and folding.^[^ ^35^ ^]^

The mesoporosity of APAA polymers was probed by porosimetry using benzene vapor (Figure 2f). The Bruanauer–Emmett–Teller (BET) surface areas ranged from 220 to 290 m^2^g^−1^ (Table S1, Supporting Information). These values are comparable to those of mesoporous polymers synthesized via radical polymerization of divinylbenzene monomers within mesoporous silica templates^[^ ^12^ ^]^ and slightly higher than those of mesoporous β‐cyclodextrin‐containing polymers^[^ ^36^ ^]^ and reticulated mesoporous polymers.^[^ ^15^ ^]^ Samples with narrower Đ and higher syndiotacticity exhibit higher surface areas, consistent with the prediction that such properties improve the quality of the self‐assembled structure. The measured pore size (5.8–7.8 nm) is slightly larger than the simulated predictions (3.5–4.0 nm). This discrepancy is attributed to structural imperfections in the synthesized material, including alkyl‐chain folding, minor chain branching, and non‐syndiotactic segments, which reduce packing efficiency and enlarge the pores. Pore characterization was carried out using benzene absorption because the N_2_ adsorption/desorption isotherms measured at 77 K were inconsistent, likely due to heterogeneous interactions between nitrogen's quadrupole moment and the chemically non‐uniform pore surface. A rigorous NLDFT analysis to obtain a pore‐size distribution was not performed, as no validated theoretical kernel exists for benzene absorption on a complex polymer surface. The use of benzene is also significant because its intercalation between the polymer's benzyl groups may induce a slightly swollen, more open state, meaning the measured pore width represents the material's functional porosity in the presence of aromatic guests. All samples exhibit Type III sorption isotherms (Figures S29–S31, Supporting Information), with hysteresis attributed to weak pi–pi interactions between benzene vapor and the absorbent, as well as pore defects. Hydrogen sorption tests (see Supporting Information‐Methods‐9) revealed H_2_ uptake of 0.96, 0.64, and 0.86 wt.% g^−1^ for PD‐4, PD‐16, and PT‐1 powders, respectively, at 77 K under 81 atm, also showing Type III sorption isotherms (Figure S32, Supporting Information). Powder and film forms exhibit similar hydrogen storage capacities (Figure S33, Supporting Information). Molecular mechanics simulations suggest exothermic hydrogen bonding in APAA Y‐clusters, except at the center position (Figure S34, Supporting Information).

### 2.4 Temperature‐Dependent Transitions Supporting the Proposed Model

**Figure 3:**
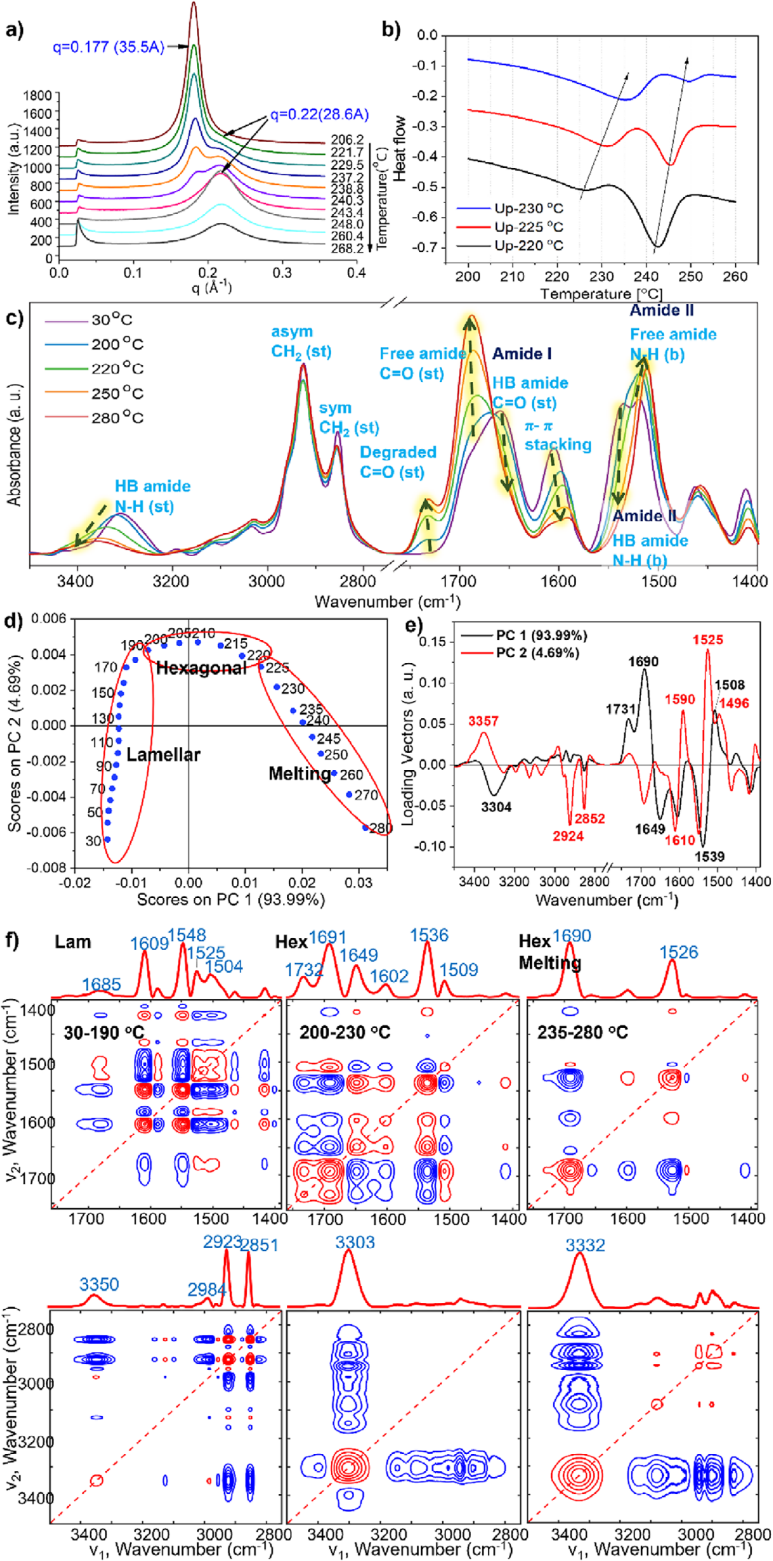
Temperature‐dependent structural analysis of PD‐14 corroborating the suggested suprastructure model: a) SAXS patterns during heating to 280 °C show a two‐step melting: transition from the hexagonal phase to a less ordered state, followed by complete disordering; b) DSC thermograms of PD‐14 with isothermal annealing near the critical temperature (T_c_ = 230 °C), showing two endothermic peaks corresponding to the two phase transitions; c) Evolution of 1D IRRAS spectra (30–280 °C) showing reduced π–π stacking and hydrogen‐bonded amide groups, and increased free amide groups. st: stretching; b: bending; d) Score plot of the first two principal components (PC1, PC2); e) Plot of the loading vectors for PC1 (black) and PC2 (red) in the N─H/ C─H stretching (3500–2750 cm^−1^) and amide I/II vibration (1750–1400 cm^−1^); f) Synchronous 2D correlation spectra in the same regions at three temperature ranges (30–190, 200–230, and 235–280 °C). Red and blue lines denote positive and negative cross peaks, respectively.

Temperature‐dependent structural analyses of APAA polymers further support the suggested molecular arrangements. SAXS data reveal a two‐step transition during melting. Upon continuous heating of PD‐14, a broad shoulder peak at q = 0.22 Å^−1^ appeared ≈220 °C. This peak disappeared below 150 °C and reemerged upon reheating to 225 °C, indicating a reversible state (Figure 2b). As the temperature increased above 220 °C, the shoulder peak grew while the 1q peak at 0.177 Å^−1^ steadily decreased (**Figure** **3**a; Figure S35, Supporting Information). At 243 °C, the melting temperature of PD‐14 identified by DSC, the 1q peak from the hexagonal cylindrical structure vanished, leaving only the peak at q = 0.22 Å^−1^. Beyond this temperature, this broad peak gradually diminished. Since APAA polymers decompose above 300 °C (Figure S36 and Table S4, Supporting Information), the disappearance of the XRD peaks indicates the loss of the ordered molecular arrangement, yielding a fully disordered state.

DSC measurements also showed two‐stage melting. Isothermal annealing near the melting temperature caused a single melting peak (Figure S15, Supporting Information) to split into two (Figure 3b). Samples subjected to three melting–annealing cycles, heated to 260 °C and annealed at 220, 225, and 230 °C, displayed consistent results in both ascending and descending temperature sequences (Figures S37 and S38 and Table S5, Supporting Information). For PD‐14, two critical transition temperatures (T_c_^1^ = 225–236 °C; T_c_^2^ = 242–250 °C) correspond to the growth and reduction of the shoulder peak at q = 0.22 Å^−1^ in SAXS. The observed two‐step transition reflects the sequential disruption of the hexagonal cylinder suprastructure and the Y‐clusters: interchain hydrogen‐bond cleavage dissociates Y‐clusters, while intrachain bond cleavage disrupts syndiotactic ordering. Similar behavior was observed for PT‐8 and PH‐2 in TGA, XRD, and DSC studies (Figures S36, S39–S41, Supporting Information).

Temperature‐dependent FT‐IRRAS studies confirm both lamellar‐to‐hexagonal and melting transitions, and the observed spectral changes align with the established structural model. Principal component analysis (PCA) of the 1D IR spectra identifies three distinct temperature regimes for PD‐14: 30–190 °C (lamellar), 190–220 °C (hexagonal cylindrical), and above 220 °C (disordered) (Figure 3c–e). The most substantial spectral changes occur above 220 °C, marking the order‐to‐disorder transition. The loading vector of PC1, which accounts for 94% of the spectral changes, shows that melting primarily involves hydrogen bond breakage between amide groups. Specifically, peaks for hydrogen‐bonded amides (3304 cm^−1^, N─H stretching; 1649 cm^−1^, C═O stretching; 1539 cm^−1^, N─H bending) have negative vectors, while free amide peaks (1690 cm^−1^, C═O stretching; 1508 cm^−1^, N─H bending) have positive vectors.^[^ ^37^, ^38^ ^]^ Amide peaks undergo significant changes only during melting, while C═C stretching (1604 cm^−1^) and C─H bending (1518 cm^−1^) in π–π stacked benzene groups^[^ ^39^ ^]^ decrease steadily with a slight redshift as temperature increases. Other APAA polymer samples show similar behavior (Figures S16, S17, and S42, Supporting Information).

2D correlation spectroscopy (2D‐COS)^[^ ^40^ ^]^ further distinguishes the lamellar and hexagonal phases of PD‐14 films below and above 200 °C (Figure 3f; Figure S43, Supporting Information). The hydrogen‐bonded amide N─H stretching peak splits into inter‐ and intrachain components at 3303 and 3332 cm^−1^, respectively.^[^ ^41^ ^]^ Interchain hydrogen bonds begin to break above 200 °C, while intrachain bonds are disrupted mainly above 235 °C. The sequence of spectral changes (**Table** **2**), determined using the Noda rule, shows that below 190 °C minor variations in CH_2_ stretching and π–π stacking dominate, supporting the model that increased alkyl‐chain mobility drives the lamellar‐to‐hexagonal transition. The order‐disorder transition between 200 and 230 °C is governed by hydrogen bond breakage, and above 235 °C, continued hydrogen bond breakage enhances alkyl chain motion, further destabilizing hydrogen‐bond networks.

**Table 2:** Sequence of transitions at different temperature ranges derived from 2D‐COS spectra of PD‐14.

T range [°C]	Sequence of transitions [cm^−1^]
30–190	2841 → 2910 → 3328 → 2854 → 1525 → 3354 → 1550 → 2928 → 1609 → 1509
200–230	1729 = 1647 → 1703 → 1541 → 1682 → 1509 → 1597 → 1527 → 2925 (weak) → 3374 (weak) → 2887 (weak) → 3303 → 2948 (weak)
235–280	2925 (weak) → 3336 → 1533 → 1598 (weak) → 1689 → 2947 = 2900 (weak) → 2839 (weak) → 1513

### 2.5 Excitation‐Dependent, Blue Fluorescence of Transparent APAA Polymers

**Figure 4:**
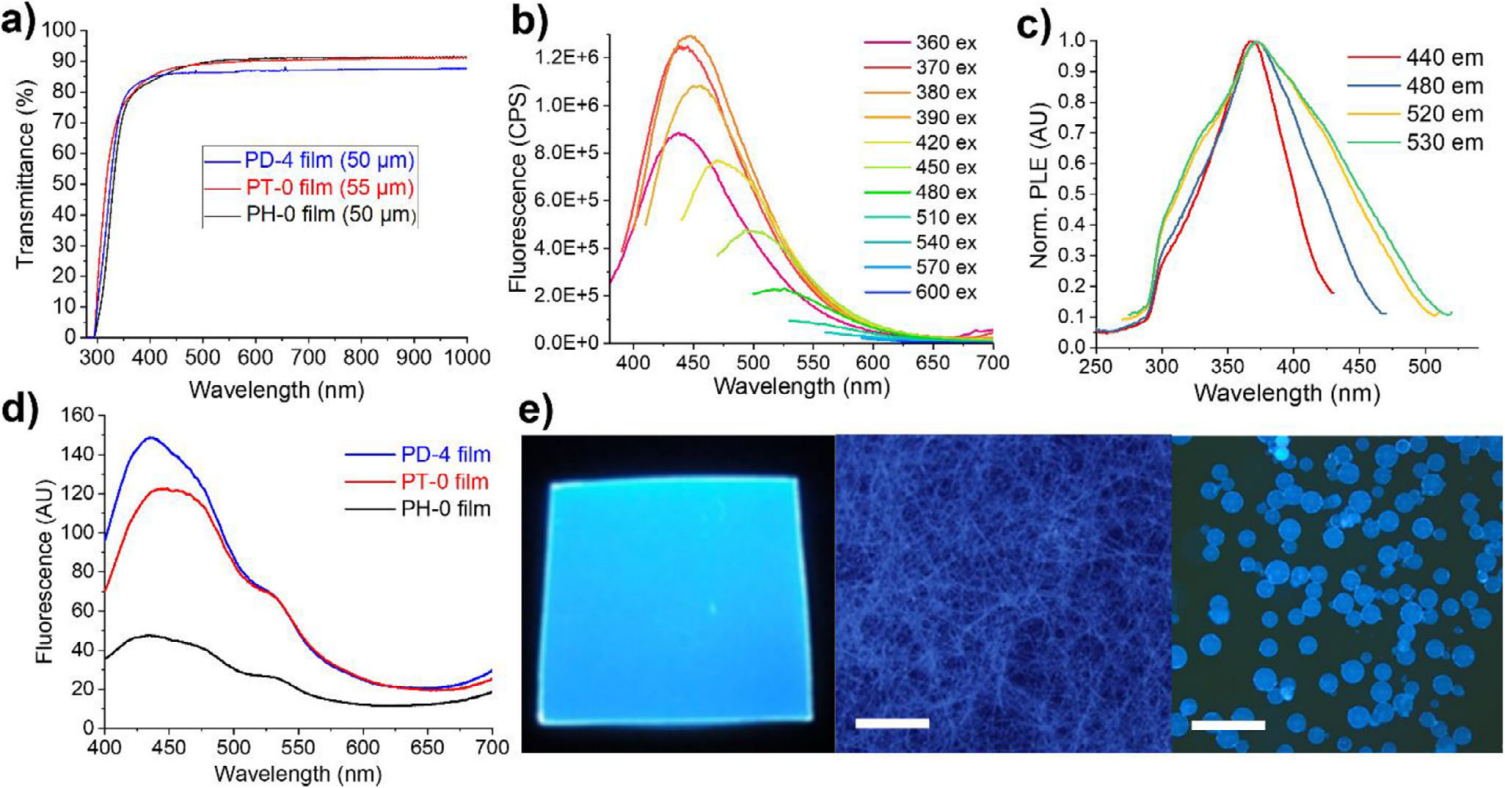
Optical properties of mesoporous APAA polymers: a) UV–vis transmittance spectra showing uniform, 85–90% transparency across the visible range; b) Fluorescence spectra of PD‐4 film exhibiting excitation‐dependent emission. *Inset*: Unnormalized spectra showing the maximal fluorescence upon excitation at 380 nm; c) Photoluminescence excitation (PLE) spectra of PD‐4 film recorded at various emission wavelengths, showing a consistent peak at 374 nm; d) Fluorescence spectra of large‐area APAA films (PD‐4, PT‐0, and PH‐0) under 365 nm excitation; e) Fluorescence images of PD‐4 film (4 cm × 4 cm × 50 µm), electrospun fibers (d = 4.5 µm) and crosslinked microparticles (d = 120 ± 20 µm). under 365 nm excitation; Scale bar: 500 µm.

The newly synthesized APAA polymers exhibit unique optical properties, combining high transparency throughout the visible spectrum, efficient UV absorption, and excitation‐dependent fluorescence with maximum fluorescence efficiency in blue. Films of 50–55 µm thickness show 85–90% transmittance above 400 nm and 75–85% transmittance in 350–400 nm (**Figure** **4**a). Transmittance remains uniform from 400 to 900 nm, with virtually no color distortion. Figure S44 (Supporting Information) compares 50 µm PD‐4 film with 25 µm commercial PET‐based UV blocking film (Decorative Films, IR8360). Despite being half the thickness of PD‐4, the commercial film exhibits lower transmittance, with a strong bias toward lower wavelengths. Additionally, APAA polymers are excellent deep UV absorbers, with transmittance dropping below 0.1% below 295 nm.

Despite minimal absorption above 350 nm, APAA polymers exhibit strong fluorescence when excited in this region. The emission peak shifts from 443 to 560 nm as the excitation 7 wavelength increases from 360 to 550 nm (Figure 4b; Figure S45, Supporting Information). Maximum emission intensity occurs with excitation at 370–380 nm, producing blue light that peaks at 440–450 nm. Various forms of APAA polymers, such as films, fibers, and microparticles, displayed comparable fluorescence characteristics (Figure 4e). Fluorescence images depicting blue, green, and red emissions from APAA polymer products, under excitation at 360, 475, and 530 nm, are presented in Figure S46 (Supporting Information). The QY of APAA polymer films excited at 365 nm ranges 7–18%, correlating with the degree of ordering (Figures S47 and S48 and Table S6, Supporting Information): Samples with high syndiotacticity (PD‐0, PD‐4, PT‐0, and PT‐1) exhibited higher fluorescence yields (10–12% at 40–50 µm and 12–16.5% at 130–160 µm) than samples with low stereoregularity (PT‐6: 7.5% at 45 µm or 8.3% at 140 µm). These results indicate that the spatial arrangement of suprastructures formed through hierarchical assembly of syndiotactic polymer chains contributes to their emission.

Despite the excitation‐dependent fluorescence, the photoluminescence excitation (PLE) spectra consistently show a peak at 374 nm, independent of the emission wavelength monitored (Figure 4c). Notably, excitation at the absorption peak (254 nm) does not produce fluorescence in any APAA polymer, likely due to cleavage of N─H bonds in amide groups by high‐energy photons (Figures S47C and S49, Supporting Information).

The fluorescence of APAA polymers likely originates from clusterization‐triggered emission (CTE).^[^ ^42^ ^]^ This phenomenon is characterized by several features: a significant emission redshift compared to single units, notable differences between excitation and absorption spectra, and excitation‐dependent emission behavior, all of which are observed in APAA polymers. The emission peaks of APAA polymers are at 440–550 nm, while that of a single benzyl unit is at 280 nm. CTE often arises from through‐space conjugation (TSC),^[^ ^43^ ^]^ where its ground state transition is optically forbidden, explaining the minimal absorption for cluster emission. In APAA assemblies, the ordered arrangement of benzyl and carbonyl groups would facilitate short‐range electron delocalization,^[^ ^44^ ^]^ driving TSC and CTE. Each syndiotactic chain has two arrays of stacked benzyl groups separated by 4.94 Å. Within Y‐clusters, benzyl groups in adjacent chains are 3.3 Å apart (Figure S22, Supporting Information). Additionally, intra‐ and intermolecular hydrogen bonding aligns carbonyl groups along the vertical axis of Y‐clusters (Figure 1c, top view). The arrangement of benzyl and carbonyl groups is nearly identical for PDOPAM, PTEPAM, and PHEPAM (Figure S25, Supporting Information), explaining the similar fluorescence behavior among APAA polymers.

Emission spectra (Figure 4d) collected from large‐area PD‐4 films revealed shoulder peaks, suggesting different aggregation states.^[^ ^45^ ^]^ Time‐resolved fluorescence measurements also showed a double exponential decay, indicating multiple fluorescent species. The faster decay component (τ_1_: 1–2 ns) accounts for 65–75% of the emission, while the slower decay component (τ_2_: 5–7 ns) contributes ≈30% (Figure S50 and Table S7, Supporting Information). These species likely include fully and partially assembled suprastructures due to branched chains or non‐syndiotactic segments.

APAA polymers represent a unique class of blue‐emitting materials. They can be easily fabricated into films using a simple process, exhibit exceptional transparency across the visible spectrum, incorporate cost‐effective chemistry, and produce robust blue emission with QY of 7–18%. Conventional conjugated blue‐emitting polymers, such as polyfluorenes, exhibit substantial absorption above 380 nm, resulting in a yellowish hue due to π‐electron transitions.^[^ ^46^ ^]^ In addition, their excitation‐dependent emission properties hold promise for anti‐counterfeiting applications.^[^ ^47^ ^]^ Beyond their intrinsic fluorescence, APAA films can be decorated with intricate patterns of higher fluorescence intensity by patterning lipid‐based organic molecules onto the films. Figure S51 (Supporting Information) presents the fluorescence images of fingerprinted PD‐4 films, which show significantly enhanced fluorescence in the fingerprint regions. The heightened fluorescence is attributed to improved refractive index matching between the fingerprint residues and the polymer films compared to the air–polymer interface. With these properties, APAA polymers can be explored for various applications.

### 2.6 Versatile Processability and Applications as VOC Absorbents

**Figure 5:**
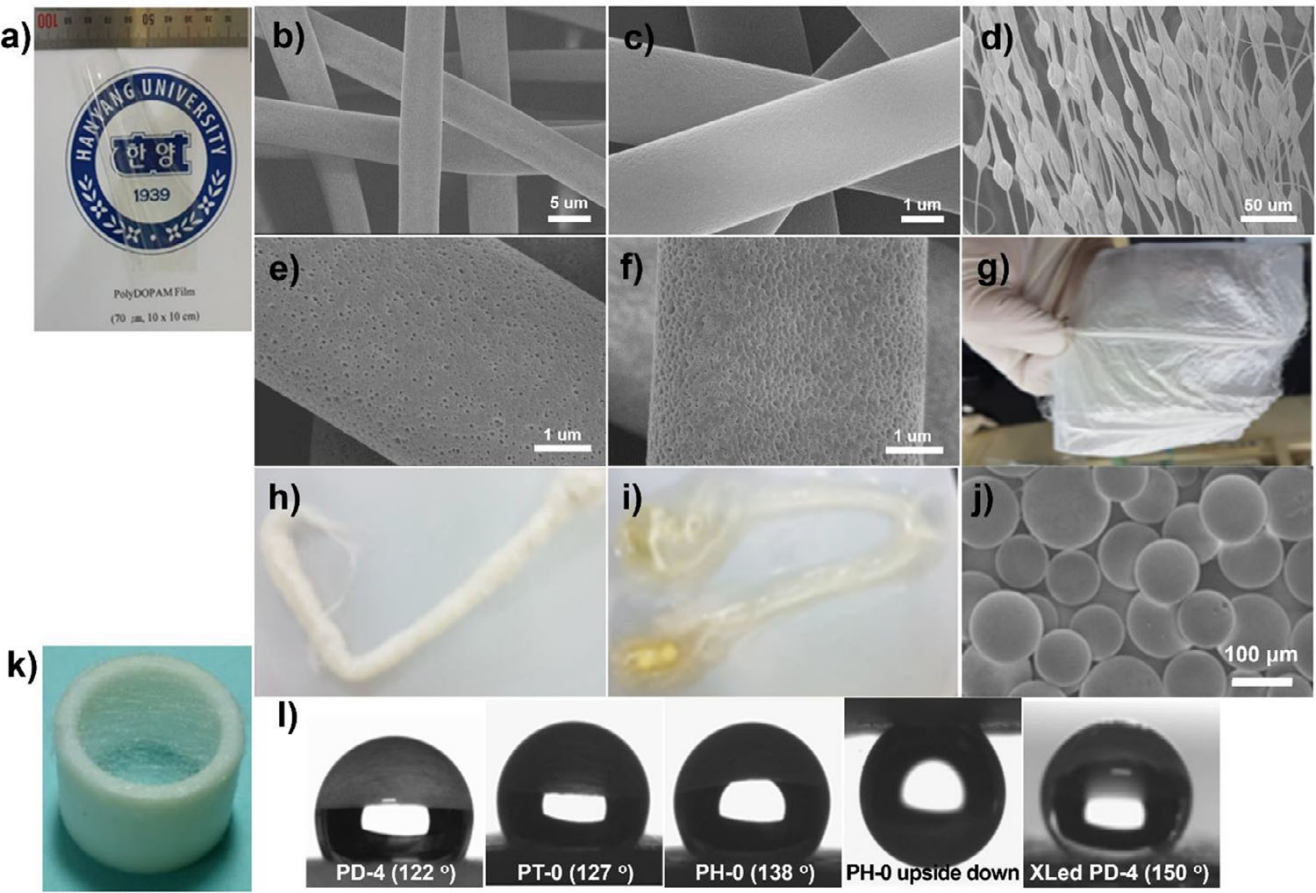
Different forms of mesoporous APAA polymers and their applications: a) Large‐area, thick film of PD‐4 (70 µm × 10 cm × 10 cm); b–f) SEM images of PD fibers prepared by electrospinning; b,c) PD‐4 fibers with diameters of 5 and 2.5 µm; d) PD‐19 fibers with spindle knots of 13–16 µm along the fiber axis (d: 4–5 µm); e,f) Surface images of PD‐4 fibers before and after UV crosslinking; g) Nonwoven fiber mat prepared from electrospun PD‐4 fibers; h,i) Crosslinked PD‐4 fiber bundles before and after swelling in benzene. The fiber bundles display an expansion of 12.5 times their weight; j) Crosslinked PDOPAM microparticles prepared by suspension polymerization using divinylbenzene as the crosslinking agent; k) Electrospun fibers coated on a Teflon O‐ring; l) Contact angle measurements of a 7 mg water droplet on electrospun fibers of PD‐4, PT‐0, and PH‐0 and on crosslinked PD‐4 fibers (d: 4–5 µm). The contact angle increases with the alkyl chain length in APAA polymers. Crosslinking further increases the contact angle, rendering the crosslinked PD‐4 fibers superhydrophobic. Despite their hydrophobicity, APAA polymers exhibit strong water adhesion, demonstrating the “petal effect”, allowing a water droplet to adhere to the fiber even in an upside‐down configuration.

APAA polymers are soluble in common solvents, such as chloroform, THF, and 1,4‐dioxane, allowing easy processing into films, fibers, and spherical particles (**Figure** **5**). Ten‐centimeter‐scale films (Figure 5a) with controlled thickness (0.01–500 µm) and high‐quality microfibers (3–10 µm diameter) and ultramicrofibers (<3 µm diameter) can be produced via casting and electrospinning,^[^ ^48^ ^]^ respectively, from a broad molecular weight range (M_n_: > 80 kDa). These properties contrast with conventional amorphous vinyl polymer, such as polystyrene, which requires stringent conditions, including ultrahigh M_n_ (150–600 kDa), narrow Đ (<1.2), and M_n_‐dependent humidity requirements.^[^ ^49^ ^]^ Representative images of (ultra)microfibers and fibers with spindle knots are shown in Figure 5b–d. SEM images show a uniform distribution of nanopores (50–100 nm) on the fiber surfaces (Figure 5e). (Ultra)microfibers and their products, such as woven mats and bundles (Figure 5g,h), have a high surface‐to‐volume ratio, rendering them invaluable for absorption, filtration, and catalysis applications. Post‐processing crosslinking with the photoinitiator bis(2,4,6‐trimethylbenzoyl)‐phenylphosphineoxide and UV irradiation^[^ ^50^ ^]^ renders APAA fibers resistant to organic solvents and creates a rough sponge‐like texture (Figure 5f). Crosslinked spherical microparticles were prepared by suspension polymerization with divinylbenzene (Figure 5j).

Mechanical properties were characterized using ductile films with wide Đ (PD‐0 and PT‐6) (Figures S52 and S53 and Table S8, Supporting Information). The ultimate tensile strength was measured to be 23 MPa for PD‐0 and 21 MPa for PT‐6. Elongation at break was 10% for PD‐0 and 14% for PT‐6, while Young's modulus was 0.5 and 0.4 GPa, respectively. These values were two to three times higher than those of reticulated nanoporous polymers.^[^ ^15^ ^]^ These mechanical properties would be further enhanced in fibers due to the longitudinal alignment of the polymer chains.^[^ ^48^ ^]^

Crosslinked APAA polymers exhibit exceptional potential to meet the challenges of large‐scale VOC remediation.^[^ ^4^, ^11^, ^51^ ^]^ Crosslinked mesoporous APAA polymer products are chemically resistant, cost‐effective, and demonstrate high swelling ratios and rapid absorption rates, making them ideal for VOC removal. Crosslinked PD‐4 fiber bundles and crosslinked microparticles absorbed chloroform at 20.7 and 16.3 times their dry weight, respectively, and 10–20 times their weight for THF, toluene, and benzene (Figure 5i; Figure S54 and Movies S1 and S2, Supporting Information). The swelling ratio can be adjusted by varying the crosslinker concentration and length. Remarkably, most VOCs reached maximum absorption in less than 10 s, far exceeding the U.S. Environmental Protection Agency guideline of 7 min for removal of low surface tension oils to reduce secondary environmental damage. The microparticles maintained their durability and performance through multiple cycles of absorption and desorption, demonstrating recyclability. Compared to existing materials, APAA polymers achieve both rapid absorption and high capacity. Polyacrylate‐based lipophilic polyelectrolyte gels^[^ ^52^ ^]^ absorb 2.5–5 times more than crosslinked APAA products, but require 48 h to reach saturation. Polydimethylsiloxane embedding mesoporous silica nanoparticles have similar capacities but take 15 min to saturate.^[^ ^53^ ^]^ Porous polymers containing β‐cyclodextrin^[^ ^36^ ^]^ reach saturation within 10 s for a wide range of organic micropollutants, but with capacities of sub‐mm or less. More importantly, these materials are not suitable for industrial‐scale synthesis due to the complex and costly chemistry involved.

Crosslinked APAA polymer fibers exhibit superhydrophobicity and high contact angle hysteresis for water, demonstrating the petal effect.^[^ ^54^, ^55^ ^]^ Crosslinked PD‐4 fibers showed higher contact angles (150°) than as‐spun fibers (PD‐4: 122°, PT‐0: 127°, and PH‐0: 138°) due to increased surface roughness (Figure 5k,l). Crosslinked PT and PH fibers are expected to yield even higher angles with longer alkyl chains. Notably, water droplets adhered strongly to the inverted fiber bed, demonstrating strong water adhesion (Figure S55, Supporting Information). High contact angle hysteresis was also observed in native APAA films (Figure S56, Supporting Information). The strong water adhesion is likely due to hydrogen bonding between water molecules and amide groups. These properties make crosslinked APAA products promising for next‐generation biofilters or membrane materials.^[^ ^56^ ^]^

## 3 Conclusion

We report a new class of vinyl polymers that self‐assemble into mesoporous structures. Traditional methods for mesoporous structures involve either multi‐step templated synthesis or bottom–up assembly using complex building blocks and specialized catalysts. In contrast, mesoporous APAA polymers enable single‐step synthesis using simple vinyl monomers, a general radical initiator, and thermal annealing. APAA polymer synthesis involves two unique mechanisms: 1) MARRS polymerization, yielding high molecular weight syndiotactic polymers without using special catalysts; and 2) spontaneous, hierarchical assembly of mesoporous structures.

The high structural regularity of APAA polymers is likely to drive their hierarchical assembly. Characterization by XRD, TEM, and porosimetry shows that APAA polymer chains spontaneously assemble into mesoporous, hexagonal cylinder structures. NMR spectrum and temperature‐dependent FT‐IRRAS suggest the pivotal role of inter‐chain hydrogen bonding in suprastructure assembly. We propose a Y‐shaped suprastructure (Y‐cluster) composed of three syndiotactic APAA polymer chains held together by inter‐chain hydrogen bonding and van der Waals interactions. The filamentous Y‐clusters then assemble into either lamellar or hexagonal cylindrical structures via van der Waals interactions. The void space between the arms of the Y‐cluster creates porosity. This model agrees well with both experimental and simulation results.

The study focused on homologs with C_12_ to C_16_ alkyl chains, as this range is known to have a low critical micelle concentration (CMC), which is crucial for the efficient formation of well‐ordered micellar mesostructures. A clear trend was observed across these samples: the C_12_ and C_14_ homologs produced well‐defined hexagonal mesostructures, supported by both experimental data and stability in simulations. However, the C16 system showed a diminished tendency to form hexagonal phases, with simulations indicating a preference for a lamellar arrangement, likely due to increased alkyl chain folding. Based on this trend, it is anticipated that longer chains, such as C_18_, would further favor lamellar packing, leading to reduced porosity. Further, shorter chains like C_8_ would be less efficient at forming regular suprastructures due to their significantly higher CMC.

The assembly mechanism of APAA polymers resembles the organization seen in spider silk and silkworm cocoon fibers, where hydrogen bonding mediates the formation of filamentous units that further assemble into suprastructures.^[^ ^57^, ^58^ ^]^ In silk fibers, β‐sheet nanocrystals formed by hydrogen bonding assemble with other β‐structures to form fibrils, which are then aligned into a fiber.^[^ ^59^ ^]^ Like silk fibers, APAA polymers can be processed into fibers by electrospinning. We have also produced fibers with spindle knots to mimic the unique structure of spider silk used for water harvesting.^[^ ^60^ ^]^ Robust fiber formation from relatively low molecular weight polymer samples (>80 kDa) supports the Y‐cluster model.

The optical and chemical data are broadly consistent with the model we propose. In particular, the excitation‐dependent emission, together with the clearly separated absorption and excitation spectra, points toward CTE through TSC, which aligns with the proposed relative positioning of benzyl and carbonyl groups within the Y‐clusters. The crosslinked fibers and microparticles also showed unusually strong uptake of liquid phase VOCs, with fast kinetics. This behavior strongly suggests mesoporosity throughout the APAA network, consistent with the cylindrical void volumes formed along the Y‐clusters in the proposed model. Finally, the strong adhesion of water droplets to the superhydrophobic fibers is also consistent with the presence of nanoscale pores in the structure.

APAA polymers present opportunities that span both fundamental studies and practical applications. Their unusual self‐assembly and polymerization process could motivate the design of new monomers that favor stereocontrol and ordered mesoporous architectures. At the same time, the relatively simple synthetic route makes them attractive for industry‐scale production. They can be processed into films, fibers, or microparticles, which makes them quite flexible for designing materials suited to specific applications. The distinctive combination of optical and surface properties makes them relevant for applications such as UV absorbers, transparent blue‐emitting films, anti‐counterfeiting coatings, and potentially for water remediation and biofiltration.

Looking forward, the structural regularity and porosity of APAA polymers could be further improved by strategies that limit chain‐transfer reactions and enhance stereoregularity, for example, by employing UV polymerization without radical initiators. Such modifications are expected to increase the BET surface area and potentially improve mechanical strength. Although moving away from MARRS polymerization may complicate industrial scale‐up, the possibility of achieving mechanical properties comparable to spider silk makes this direction highly promising.

## 4 Experimental Section

The Supporting Information provide comprehensive data on reagents, materials, monomer and polymer syntheses, stereoregularity of polymer chains, characterization methods for thermal, morphological and physicochemical properties. It also includes details on molecular mechanics simulation for polymer suprastucture prediction, hydrogen gas storage, mechanical properties, preparation methods for polymer films, fibers, and spherical microparticles, along with the solvent absorbency and absorption rate of crosslinked polymer products.

### Synthesis of p‐Alkyl‐N‐Phenyl‐Acrylamide (APAA) Monomers

APAA monomers (TEPAM and HEPAM, Figures S1–S3, Supporting Information) were synthesized by the same method as the DOPAM synthesis reported in the previous study.^[^ ^24^ ^]^ Yield (%): DOPAM, 86; TEPAM, 82; HEPAM, 84. T_m_ (°C): DOPAM, 101; TEPAM, 103; HEPAM, 107. ^1^H NMR (400 MHz, CDCl_3_): DOPAM: δ7.5 (d, 2H), 7.2 (s, 1H), 7.15 (d, 2H), 6.4 (d, 1H), 6.2 (q, 1H), 5.8 (d, 1H), 2.6 (t, 2H), 1.62–1.20 (m, 20H), 0.935 (t, 3H) ppm; TEPAM 7.5 (d, 2H), 7.2 (s, 1H), 7.15 (d, 2H), 6.4 (d, 1H), 6.2 (q, 1H), 5.8(d, 1H), 2.6 (t, 2H), 1.62–1.20 (m, 24H), 0.935 (t, 3H); HEPAM: 7.5 (d, 2H), 7.2 (s, 1H), 7.15 (d, 2H), 6.4 (d, 1H), 6.2 (q, 1H), 5.8 (d, 1H), 2.6 (t, 2H), 1.62–1.20 (m, 28H), 0.935 (t, 3H).

### Preparation of APAA Polymers

The monomer DOPAM (3. 0 g, 9.52 mmol, 10% solution in benzene) was added to a 50 mL ampoule together with the solvent benzene (30.80 mL) and BPO (20.1 mg, 0.083 mmol) used as radical initiator. The solution was deoxygenated twice by the freeze–thaw method, and the ampoule was sealed and then placed in a thermostat preheated to 70 °C to perform radical polymerization for 30 min. At the end of the polymerization reaction, 10 mL of THF was added to the ampoule to dilute the polymerization solution. The solution was precipitated with methanol (300 mL) and then filtered to obtain a pale yellowish fibrous solid. The solid was dissolved in THF (20 mL) and reprecipitated with an excess of methanol (200 mL) to remove traces of unreacted monomer and low molecular weight polymer. The solid obtained was dried in a vacuum oven at 25 °C for 24 h to give pale yellow pure PolyDOPAM‐4 (PD‐4) (conversion, 60.3%; M_n_, 134900; Đ, 1.93). In addition, other APAA polymers (PD, PT, PH) with a wide range of number average molecular weight (M_n_) and molecular weight distribution (Đ) were prepared under different conditions (Table S1, Supporting Information). The polymerization parameters include monomer concentration (2, 5, 10, or 20 wt.%) to solvent, radical initiator concentration (BPO or AIBN), solvents with different dielectric constants (benzene, toluene, dioxane, monoglyme, THF, or DMF), temperature (70, or 80 °C), and time (0.5, 1, or 48 h).

### Preparation of APAA Polymer Films

The thickness of the film is controlled by the concentration of the solution and the molecular weight of the polymer. The polymer was dissolved in THF or CHCl_3_ to give a polymer solution in the range of 2–10 wt.%. The polymer solution filtered through a plastic membrane filter was poured onto a glass plate with a rectangular Teflon frame (3 × 7 × 1 cm or 10 × 10 × 1 cm), dried in the atmosphere for 24 h, and then dried in a vacuum oven for 24 h to make a *thick film* with a thickness of 10–330 µm. The polymer was also dissolved in THF or CHCl_3_ to make a 2.0% solution. 1.0 mL of the polymer solution filtered through a plastic membrane filter was dropped onto a silicon wafer (1 × 1 cm) and coated with a spin coater at 3000 rpm to produce a *thin film* of 25 ± 2 nm.

### Preparation of APAA Polymer Fibers by Electrospinning

PD‐4 (1.0 g) in Table 1 was dissolved in 2.9 mL (or 3.4 mL) of THF to prepare a 28 wt.% (or 25 wt.%) polymer solution. The polymer solution was filled into a syringe and electrospun using an electrospinning device (Figure S57A, Supporting Information; NanoNC Model ESR‐200RD, Korea). The polymer solution in the syringe was electrospun on a conventional stainless steel drum‐type collector through a 25‐gauge stainless steel needle (diameter, d = 0.51 mm) under the conditions of an applied voltage of 5 kV (or 20 kV), a spinning rate of 15 mL min^−1^, and a distance of 12 cm between the needle and the collector. As a result, PD‐4 fibers with diameters of 5.0 and 2.5 µm (Figure 5b,c), respectively, were obtained. In addition, PT‐0 and PH‐0 were also electrospun under the same conditions to obtain (ultra)microfibers for contact angle measurements against water.

### Preparation of Crosslinked PD‐4 Fibers

PD‐4 (1.0 g) and bis(2,4,6‐trimethylbenzoyl)‐phenylphosphineoxide (B‐PPO, photoinitiator) were dissolved in 2.9 mL of THF to prepare a 28 wt.% polymer solution. The polymer solution in the syringe was electrospun through the needle onto the aluminum collector (Figure S57, Supporting Information) under the conditions of 28 wt%, 10 kV, 30 mL min^−1^, and 7 cm. Crosslinked fibers were prepared by irradiating the fiber surface with UV (λ_max_ = 365 nm, intensity = 200 mW cm^−2^) for 400 s while rotating (speed = 3.0 m min^−1^) the aluminum collector wound with the aligned PD‐4 fibers containing the photoinitiator B‐PPO in a home‐made UV irradiator equipped with a mercury lamp. The surface of the crosslinked fibers has a sponge‐like morphology (Figure 5f). The diameter of the fibers produced depends on variables such as the molecular weight of the homopolymer (M_n_ > 80 000), the polymer concentration in the solvent (chloroform or THF), the voltage applied, the nozzle size, and the distance between the nozzle and the collector. In addition, detailed preparation methods for aligned fibers (Figure S57B, Supporting Information) with spindle knots, nonwoven mats, and absorbent fiber bundles are given in the Supplementary Material.

### Preparation of Crosslinked PDOPAM Particles by Suspension Polymerization

A monomer solution was prepared by dissolving 3 g (9.51 mmol) of monomer DOPAM, AIBN (0.03 g, 0.18 mmol) as initiator, and divinylbenzene (DVB, 0.066 mL, 0.46 mmol) as a crosslinking agent in benzene (15 mL). An aqueous solution prepared by dissolving a suspending agent (1.2 g, Mowiol 40) in distilled water (120 mL) was placed in a 250 mL three‐necked jacket flask equipped with a mechanical stirrer, and the previously prepared monomer solution was added to the flask at a rate of 0.03 mL s^−1^ through a dropping funnel. Suspension polymerization was then carried out at 70 °C for 9 h, with the monomer solution being added to the flask at a constant rate. The stirring speed during the polymerization reaction was 250 rpm. At the end of the polymerization reaction, the polymerization solution was precipitated in 1500 mL of methanol, stirred at room temperature for 3 h, and filtered to obtain crosslinked PD particles. In this step, unreacted monomer, suspending agent, water, and benzene were dissolved in methanol and separated. The filtered crude particles were reintroduced into 200 mL of methanol, stirred at room temperature for 2 h, and filtered to obtain pure crosslinked PD particles. The particles were dried in a vacuum oven at room temperature for 24 h, yielding white crosslinked spherical PD microparticles (Figure 5j) of 110–140 µm (mean diameter = 120 µm). The polymerization conversion was 88.3%.

### Determination of Polymer Stereoregularity by ^13^C NMR Spectroscopy

The triad tacticity describes isotactic, syndiotactic, and heterotactic (so‐called atactic), whose fractions are designated as (mm), (rr), and (mr), respectively. The atactic triad (mr) has no stereoregularity between the repeating units.^[^ ^61^ ^]^ Tacticity, the ratio of [syndiotactic] to [isotactic], was determined by quantifying the relative proportions of racemic (rr) to meso (mm) triads in the solution phase ^13^C NMR (Bruker Avance III HD 500) spectrum (Figure S11, Supporting Information). The quantitative area ratio of rr to mm was calculated using commercially available Origin or Igor software (see Supporting Information‐Chacracterization‐1 for details, Figures S12–S14, Supporting Information). The methyn (or alpha, α) carbon^*^ (─CH_2─_^*^CH‐L) peak in the backbone chain of PD depends on the stereoregularity of the side chain (L) in adjacent repeat units. For example, the α‐carbon peak in a typical polyacrylamide backbone chain is split into three peaks appearing at 43.20, 42.82, and 42.38 ppm, respectively, which are assigned in the ^13^C NMR literature as syndiotatic, atactic, and isotactic triads.^[^ ^62^ ^]^ When THF‐d_8_ was used as the NMR solvent, the methyn peak of the new APAA polymers had a slightly higher value (0.2 ppm) than that of the polyacrylamide, the reference sample, due to the benzene group substituted on the amide linkage. With CDCl_3_ the methyn peak shifted 1.0 ppm higher than with THF‐d_8_. Unfortunately, the atactic triad was indistinguishable from the syndiotactic and isotactic triads even in the 600 or 700 MHz ^13^C NMR spectra. ^13^C CP MAS solid state NMR spectra of DOPAM monomer and APAA polymers (Figures S7–S10, Supporting Information) were recorded on a Bruker AV 600 MHz spectrometer using a standard Bruker Magic Angle Spinning (MAS) probe with 4 mm zirconia rotors to confirm the characteristic carbon peaks of the mesoporous APAA polymers.

### X‐Ray Diffractometry Measurements

Synchrotron X‐ray (SAXS) measurements were performed at the 9A beamline at the Pohang Accelerator Laboratory (PAL) in Korea to investigate the morphological structures of APAA polymers. The wavelength of the X‐ray was 1.119 Å, and the distance from the sample to the detector was 2.6 m. The exposure time for each image was 1 s. The SAXS patterns were recorded by a 2D X‐ray detector using a CCD (PI‐SCX4300‐165/2, Princeton Instrument). The SAXS patterns were measured after holding the polymer samples for 10 min at predetermined temperatures for both heating from 25 to 225 °C and cooling from 225 to 25 °C to minimize the temperature influence. For continuous heating measurements, the sample was continuously heated from 25 to 280 °C at the rate of 5.0 °C min^−1^, with spectra acquired every ≈1.5 °C. 1D WAXD patterns were obtained using an X‐ray radiation generator (CuK_α_, λ = 1.5417 Å) coupled to a diffractometer (Rigaku, Japan) at room temperature or −70 °C. Silicon crystal powder was used as an internal reference showing a diffraction ring at 2θ = 28.466°.

### DSC Experiments with Isothermal Annealing Near the Melting Temperature

Multi‐step DSC experiments consisting of three cycles of melting at 260 °C and isothermal annealing at 220, 225, and 230 °C (Figure S37, Supporting Information) were performed. These cycles were performed in both ascending and descending sequences, with DSC curves measured after each annealing step. The identified T_c_^1^ and T_c_^2^ and their enthalpies are summarized in Table S5 (Supporting Information). Two‐phase transition peaks were detected at all annealing temperatures, and T_c_^1^ and T_c_^2^ remained consistent regardless of the annealing cycle history. It was also observed that higher annealing temperatures resulted in higher T_c_^1^ and T_c_^2^.

### Temperature‐Dependent IR Spectroscopy

The temperature‐dependent infrared reflection absorption spectrum (IRRAS) of PD‐14, PT‐8, and PH‐2 was measured in 5 °C increments in the range of 25–280 °C. To measure IRRAS, the polymer films were prepared by casting their chloroform solutions on a Pt‐coated silicon wafer and kept in a vacuum drying oven at room temperature for 12 h. In order to study the temperature dependence of the polymer suprastructures (lamellae and hexagonal cylinders), the synchronous and asynchronous 2D correlation spectra were calculated using the algorithm based on the numerical method developed by Noda and executed in MATLAB. PCA^[^ ^63^, ^64^ ^]^ was carried out using PLS_Toolbox Ver. 4.2 (Eigenvector research, Inc., Wenatchee, WA) for MATLAB.

### Molecular Mechanics Simulation

To gain insight into the structural changes during the phase transition from lamellar to hexagonal cylindrical nanostructures observed in the XRD and DSC results, and to predict the 3D nanostructures of APAA polymers, molecular mechanics simulations were performed. These simulations, conducted using NAMD^[^ ^33^ ^]^ based on the optimized potentials for liquid simulations (OPLS) force field,^[^ ^65^ ^]^ employed a periodic boundary condition (PBC) and a conjugated gradient method for structural optimization.

### Transmission Electron Microscopy

TEM was performed at 300 kV using a JEOL‐JEM 3010. Ultrathin sectioning of the samples was performed on an ultramicrotome using a Leica EM UC7. Prior to ultrathin sectioning, the samples were aligned by annealing at 250 °C for 2 h. Thin sections of the samples were transferred to a carbon‐coated copper grid and stained with R_u_O_4_ vapor. Data were analyzed using Digital Micrograph software.

### BET Surface Area Analysis

To determine the surface area of novel mesoporous APAA polymers, their benzene vapor absorption and desorption isotherms were recorded on BELSORP‐max. Prior to measurement, all polymer samples were annealed at 200 °C for 1 h to activate polymer chains with hexagonal cylindrical nanostructures based on DSC and XRD results, and then 110 to 330 mg of polymer powder samples were degassed under vacuum at 120 °C for 1 h. The surface area of each sample was calculated by the Brunauer–Emmett–Teller (BET) method^[^ ^66^ ^]^ using adsorption data at relative pressures (P/P_o_) ranging from 0.01 to 0.5 at standard temperature and pressure (STP, 25 °C, 1 atm). The cross‐sectional area^[^ ^67^ ^]^ of the adsorbed benzene molecule is 0.430 nm^2^. The total pore volume is the amount of benzene molecules adsorbed in the monolayer at a P/P_o_ of 0.90.

### Hydrogen Gas Storage Measurements

The absorption of hydrogen gas at high pressures up to 90 bar was measured volumetrically^[^ ^68^ ^]^ using a pressure‐composition isotherm (PCT, BELSORP‐HP, BEL JAPAN). Hydrogen gas of 99.9999% purity was used for all H_2_ sorption measurements. For the PCT measurement, the system was calibrated with LaNi_5_ at room temperature and with activated carbon (surface area ≈3000 m^2^ g^−1^) at 77 K, respectively. PD‐4 film prepared with a thickness of 100 µm was cut into 2.0 × 1.0 cm and then the films of 150 ± 10 mg (or PD‐4 powder, 150 ± 10 mg) were degassed before measurement and heated at 120 °C until a pressure of 4–8 × 10^−7^ mbar was reached. The desired hydrogen pressure was then introduced into a thermostatic chamber, and once equilibrium was reached, the gas was allowed to expand in a sample holder. The measured pressure drop was caused by gas expansion within the sample holder and hydrogen adsorption into the sample. If no further pressure change was observed, a further 300 s was allowed to elapse until thermal equilibrium was reached.

### Mechanical Property Measurements

The mechanical properties (ASTM D882) of APAA polymer films (thickness, 60–65 µm) cast from 5% chloroform solution were measured at a crosshead speed of 10 mm min^−1^ using an Instron Tensile Tester (Model 4201). For each sample, mechanical tests were performed on three specimens (60 mm × 5 mm). Modulus (MegaPascal, MPa), ultimate stress (MPa), stress at break (MPa), and strain at break (%) are the average of the mechanical properties obtained from the stress–strain curves of the three specimens.

### Fluorescence Microscopy

Films, fibers, or crosslinked polymer microparticles were placed on a microscope slide and their red, green and blue (RGB) images (Figure S46, Supporting Information) were observed using a fluorescence microscope (OLYMPUS BX51) in red (U‐MWG2 filter; excitation: 510–550 nm; emission: >590 nm), green (U‐MWB2 filter; excitation: 460–490 nm; emission: >520 nm) and blue (U‐MWU2 filter; excitation: 330–385 nm; emission: >420 nm). The images emitted by the sample were captured using a CCD camera (DP 70) with an exposure time of 300 ms for films and fibers, but 400 ms for microparticles.

### Fingerprint Storage and Reading

After printing a thumbprint on PD‐4 transparent film (60 µm), a photograph (left side in Figure S51, Supporting Information) was taken with a Sony Alpha 6000 digital camera equipped with a macrolens (Sony SEL30M35) while exposed to a 365 nm portable UV lamp (2.5 mW cm^−2^). The contrast of the photograph was increased by 40% using the PowerPoint program (Microsoft Co. Ltd.). Enlarged RGB images (right side) of the thumbprint were measured using the fluorescence microscope with an exposure time of 555 ms. The RGB images were corrected for noise using standard noise reduction software. The measurement procedure is the same as described for the RGB fluorescence image of the film.

### Quantum Yield Measurements

The photoluminescence quantum yield (QY) of APAA polymer films was measured using an optical setup equipped with an integrating sphere (IS200‐4, Thorlabs) and a spectrophotometer (HR2000+CG‐UV‐NIR, Ocean Optics). The sample was illuminated with a 365 nm LED (M3655FP, Thorlabs). A 400 nm long pass filter (FELH400, Thorlabs) was used to block the excitation beam. The spectrophotometer was calibrated using a calibrated silicon photodetector (UV‐818, Newport). The experimental procedure^[^ ^69^ ^]^ for QY measurements is shown in Figure S48 (see Supporting Information for the calculation formula of QY).

### Measurement of VOC Absorption Properties of Crosslinked Polymers

Solvent absorbency was measured by placing 0.2 g of crosslinked polymer microparticles or 30 ± 3 mg of crosslinked fiber bundle in a beaker filled with 10 cc of VOC (benzene, chloroform, THF, toluene, n‐octane, or DMF), and retrieving them after 30 min for the microparticles or 10 min for the bundle. The polymer gel swollen in the solvent was filtered through a 100 mesh stainless steel sieve for 10 min and weighed on an electric balance. Absorbency (swelling ratio) for a given solvent was calculated by dividing the weight of the swollen polymer gels by the weight of the dried polymer particles (ASTM F726‐81). Absorption rate was measured using an optical microscope (Keyence VH‐Z100UR). The absorption rate was defined as the time taken for the polymer microparticle to reach its maximum absorption capacity for a given solvent (see Supporting Information for details and additional Movies S1 and S2, Supporting Information).

## Conflict of Interest

The authors declare no conflict of interest.

## Author Contributions

S.H.K., J.J., and T.W.P. performed synthesis and polymerization. Electrospinning was performed by S.H.K. and J.J. Measurement and Data acquisition: DSC (J.J., H.H.S., and T.W.P.), XRD (M.K.K. and H.H.S.), FT‐IR (Y.P.and Y.M.J.), Porosimetry (W.K.M.), TEM (E.L.), QY (G.H.), ^13^C NMR for tacticity (M.H.J. and J.J.), ^13^C CP MAS solid state NMR (Y.L. and D.S.), Hydrogen sorption (W.K.H.) and Fluorescence (J.J. and H.‐S.H.). Data analysis and Writing: DSC (H.H.S., Y.‐K.H. and H.‐S.H.), XRD (H.H.S., Y.‐K.H. and H.‐S.H.), FT‐IR (Y.M.J. and H.‐S.H.), Porosimetry (W.K.M. and K.B.Y.), TEM (E.L.), QY (G.H.), Tacticity (M.H.J., T.W.P., J.J. and S.H.S.), Solid state NMR (Y.‐K.H. and D.S.), Hydrogen sorption (W.K.H. and Y.‐K.H.), and Fluorescence (S.H.K., H.‐S.H. and Y.‐K.H.). Molecular mechanics simulation: S.U.L. Y.‐K.H. conceived the idea and designed the experiments. Y.‐K.H. and H.‐S.H. analyzed the data and prepared the manuscript and the Supporting Information.

## Supporting information



Supporting Information

Supplemental Movie 1

Supplemental Movie 2

## Data Availability

The data that support the findings of this study are available in the supplementary material of this article.
