# Supplementary material for: Single‐Step Synthesis of Mesoporous Vinyl Polymers via Hierarchical Assembly of Stereocontrolled Chains and Their Unique Properties
Source: Small. 2025 Nov 9;21(51):e09954. doi: 10.1002/smll.202509954 (PMC12723350; doi:10.1002/smll.202509954)
Supplement: Supplementary file 1 — Supporting Information [file SMLL-21-e09954-s003.pdf]

NANO · MICRO
small

Supporting Information

for *Small*, DOI 10.1002/smll.202509954

Single-Step Synthesis of Mesoporous Vinyl Polymers via Hierarchical Assembly of Stereocontrolled Chains and Their Unique Properties

Su Hwa Kim, Jin Jo, Tae Won Park, Young Mee Jung, Hyun Hoon Song, Sang Uck Lee, Yeonju Park, Won Kyung Moon, Eunji Lee, Gyu Weon Hwang, Min Kwan Kang, Min Hwan Jung, Won Gi Hong, Daewon Sohn, Youngil Lee, Kyung Byung Yoon, Seung Hoon Shin, Hee-Sun Han and Yang-Kyoo Han**

Supporting Information for

Single-Step Synthesis of Mesoporous Vinyl Polymers via Hierarchical Assembly of Stereocontrolled Chains and Their Unique Properties

Su Hwa Kim *et al.*

Co-corresponding authors: Yang-Kyoo Han, ykhan@hanyang.ac.kr; Hee-Sun Han, hshan@illinois.edu

The PDF file includes:

Supplementary Text

1. Abbreviation
2. Reagents
3. Materials 1 to 6
4. Methods 1 to 11
5. Characterization of physicochemical properties 1 to 8
6. Legends for Movies S1 and S2 in a separate file
7. Figures S1 to S57
8. Tables S1 to S8
9. References

Table of Contents

1. Abbreviation --3

2. Reagents --4

3. Materials --4

1) Synthesis of crystalline p-alkyl-N-phenyl-acrylamide monomers ------------------------------------4

2) Preparation of APAA polymers --5

3) Single crystal structure of DOPAM monomer ---5

4) Preparation of APAA polymer films ---5

5) Production of APAA polymer fibers by electrospinning --6

6) Preparation of crosslinked PDOPAM particles by suspension polymerization --------------------6

4. Methods 1 to 11 --7

5. Characterization of physicochemical properties of APAA polymers

1) Determination of polymer stereoregularity by ^{13}C NMR spectroscopy -----------------------------8

2) X-ray diffractometry measurements ---9

3) DSC experiments with isothermal annealing near the melting temperature ---------------------10

4) Temperature dependent IR spectroscopy ---10

5) Molecular mechanics simulation ---10

6) Fluorescence spectroscopy ---10

7) Quantum yield measurements ---11

8) Measurement of VOC absorption properties of crosslinked plymers ------------------------------11

6. Legends for Movies S1 and S2 in a separate file ---12

7. Figures S1 to S57 ---13

8. Tables S1 to S8 ---58

9. References ---64

1. Abbreviation

AIBN:	2,2-azobisisobutyronitrile
AIEE:	aggregation-induced enhanced emission
APAA:	p-alkyl-N-phenyl-acrylamide
AWH:	atmospheric water harvesting
BET:	Brunauer-Emmett-Teller
BPO:	benzoyl peroxide
B-PPO:	bis(2,4,6-trimethylbenzoyl)-phenylphosphineoxide
^{13}C CP-MAS:	carbon-13 cross-polarization magic angle scanning
CTE:	clusterization-triggered emission
2D-COS:	two dimensional correlation analysis
DOPAM:	p-dodecyl-N-phenyl-acrylamide
DSC:	differential scanning calorimetry
FT-IRRAS:	Fourier-transform infrared reflection absorption spectroscopy
GPC:	gel permeation chromatography
HIPE:	high internal phase emulsion
HEPAM:	p-hexadecyl-N-phehyl-acrylamide
MARRS polymerization:	monomer aggregation-mediated rapid, radical stereo-controlled polymerization
MMP:	micro- and meso-porous
MW:	number average molecular weight (M_n)
NAMD:	nanoscale molecular dynamics
NMR:	nuclear magnetic resonance
OPLS:	optimized potential for liquid simulations
PCA:	principal component analysis
PC1:	first principal component
Đ:	Dispersity (molecular weight distribution, polydispersity index)
PDOPAM:	poly(p-dodecyl-N-phenyl-acrylamide)
PD/PT/PH:	PDOPAM/PTEPAM/PHEPAM
PE/PP/PS/PVC:	polyethylene/polypropylene/polystyrene/poly(vinyl chloride)
PLE:	photoluminescence excitation
QY:	quantum yield
SAXS:	small angle x-ray scattering
SEM:	scanning electron microscopy
SM:	supplementary materials
TEM:	transmission electron microscopy
TEPAM:	p-tetradecyl-N-phenyl-acrylamide
TGA:	thermogravimetric analyzer
THF:	tetrahydrofuran
T_A:	annealing temperature
T_m:	melting temperature (order-to-disorder transition temperature)
T_C^1 (T_C^2):	first (second) critical phase transition temperature
TSC:	through-space conjugation
UV:	ultraviolet
VOCs:	volatile organic compounds
WAXD:	wide angle x-ray diffraction
XRD:	x-ray diffractometry

2. Reagents

4-Dodecylaniline (97%), 4-tetradecylaniline (97%), 4-hexadecylaniline (97%), polymerization solvents (HPLC grade) such as benzene, toluene, 1,4-dioxane, monoglyme (1,2-dimethoxyethane), tetrahydrofurane (THF), and N,N-dimethyl formamide (DMF) were used as received from Sigma-Aldrich without further purification. Acryloyl chloride (Aldrich, 97%) was fractionally distilled over calcium hydride before use. 2,2'-Azobisisobutyronitrile (AIBN, Aldrich, 99%) and benzoyl peroxide (BPO, Aldrich, 75% in water as stabilizer), radical initiators, were recrystallized from ethanol. Other reagents were used as received unless noted. All materials were identified by ^{1}H nuclear magnetic resonance (^{1}H-NMR) spectroscopy, using a Varian Gemini 400 MHz spectrometer with TMS as a reference solvent.

3. Materials

1) Synthesis of crystalline p-alkyl-N-phenyl-acrylamide monomers

p-Dodecylaniline (12 g, 0.046 mol) was dissolved in THF solvent (100 mL). The solution was transferred into a 100mL three-necked round flask, and an acid eliminator containing imidazole and triethyl amine at the same molar ratio (0.023 mol) was added dropwise through a funnel for 10 min. Under a nitrogen atmosphere, a solution containing acryloyl chloride (3.8 mL, 0.047 mol) in THF (20 mL) was gradually added dropwise to the mixed solution through a dropping funnel for 20 min. Meanwhile, the solution was cooled on an ice bath to prevent the temperature of the reaction mixture from rising above 5°C. After 6 h of reaction at 0°C, the solution was kept at 25°C for a further 9 h of reaction. At the end of the reaction, the solution was passed through a filter paper to remove precipitated salts and the solvent was evaporated from the filtrate on an evaporator. The solid thus obtained was dissolved in dichloromethane (100 mL) and added to a separate funnel together with a 10% aqueous solution of $NaHCO_3$ (50 mL). The funnel was shaken vigorously and set aside to allow complete separation of the aqueous phase, thereby removing unreacted acryloyl chloride. Magnesium sulfate (1.0 g) was added to the separated dichloromethane solution. After stirring for 5 h, the solution was filtered to remove the traces of water dissolved in the solvent. The dichloromethane solution thus obtained was kept on the evaporator, and n-hexane (100 mL) was added. The solution was stirred for 2 h and unreacted p-dodecyl aniline was filtered from the solution. The solvent was then removed from the filtrate solution by evaporation to give a white solid p-dodecyl-N-phenyl-acrylamide (**DOPAM**, yield=86%). DOPAM product (T_m=101°C) was purified by recrystallization with ethanol three times and then used for polymerization (**Figure S1)**[24].

In addition, new p-tetradecyl-N-phenyl-acrylamide (**TEPAM**, yield=82%) and p-hexadecyl-N-phenyl-acrylamide (**HEPAM**, yield=84%) monomers were synthesized by the same method as described above. Crude TEPAM (T_m=103°C) and HEPAM (T_m=107°C) monomers were also recrystallized three times with ethanol prior to polymerization (**Figure S2**).

The chemical structure of p-alkyl-N-phenyl-acrylamide (**APAA**) monomers (DOPAM, TEPAM, and HEPAM) was identified from their ^{1}H NMR spectra.

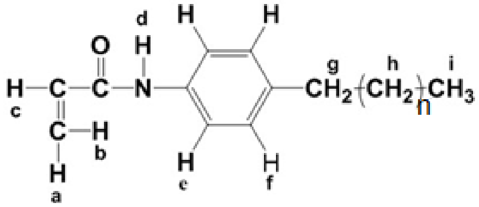

^{1}H NMR(400MHz, $CDCl_3$) for **DOPAM** (n=10): e, 7.5(d, 2H); d, 7.2(s, 1H); f, 7.15(d, 2H); b, 6.4(d, 1H); c, 6.2(q, 1H); b, 5.8(d, 1H); g, 2.6(t, 2H); h, 1.62–1.20(m, 20H); i, 0.935(t, 3H) ppm; for

TEPAM (n=12, **Figure S3**): e, 7.5(d, 2H); d, 7.2(s, 1H); f, 7.15(d, 2H); b, 6.4(d, 1H); c, 6.2(q, 1H); b, 5.8(d, 1H); g, 2.6(t, 2H); h, 1.62–1.20(m, 24H); i, 0.935(t, 3H); for **HEPAM** (n=14): e, 7.5(d, 2H); d, 7.2(s, 1H); f, 7.15(d, 2H); b, 6.4(d, 1H); c, 6.2(q, 1H); b, 5.8(d, 1H); g, 2.6(t, 2H); h, 1.62–1.20(m, 28 H); i, 0.935(t, 3H).

2) Preparation of APAA polymers

Representative **M**onomer **A**ggregation-mediated **R**apid, **R**adical **S**tereocontrolled (**MARRS**) polymerization for Entry PD-4**:** The monomer DOPAM (3. 0 g, 9.52 mmol, 10% solution in benzene) was added to a 50 mL ampoule together with the solvent benzene (30.80 mL) and BPO (20.1 mg, 0.083 mmol) used as radical initiator. The solution was deoxygenated twice by the freeze-thaw method, and the ampoule was sealed and then placed in a thermostat preheated to 70° C to perform radical polymerization for 30 min. At the end of the polymerization reaction, 10 mL of THF was added to the ampoule to dilute the polymerization solution. The solution was precipitated with methanol (300 mL) and then filtered to obtain a pale yellowish fibrous solid. The solid was dissolved in THF (20 mL) and reprecipitated with an excess of methanol (200 mL) to remove traces of unreacted monomer and low molecular weight polymer. The solid obtained was dried in a vacuum oven at 25°C for 24 h to give pale yellow pure PolyDOPAM-4 (PD-4) (conversion=60.3%; M_n=134,900; PDI=1.93).

In addition, other APAA polymers (PD, PT, PH) with a wide range of number average molecular weight (M_n) and molecular weight distribution (polydispersity index, PDI) were prepared under different conditions (**Table 1; Table S1)**. The polymerization parameters include monomer concentration (2, 5, 10, or 20 wt%) to solvent, radical initiator concentration (BPO or AIBN), solvents with different dielectric constants (benzene, toluene, dioxane, monoglyme, THF, or DMF), temperature (70, or 80 ^{o}C), and time (0.5, 1, or 48 h).

Large-scale synthesis: Using a 200 mL glass ampoule, novel APAA polymers up to 10 g scale were prepared from DOPAM, TEPAM, and HEPAM via **MARRS** polymerization first developed in this study to evaluate the feasibility of large scale synthesis (**Figure S4**).

3) Single crystal structure of DOPAM monomer

We reported that the single crystal of DOPAM synthesized for the first time in our laboratory has a monoclinic ($P2_1/c$) structure, one of the 14 Bravais lattice structures.[24] The interlayer distance of the dodecyl alkyl chains introduced into DOPAM was 0.36 nm, and the distance between the alkyl groups was 0.365 nm when hydrophobic interactions were present. The distance of 0.369 nm from the layer plane to the neighboring plane (C_{16}) was also exactly the same. Intermolecular hydrogen bonds were formed between the layer and the layer through amide (-CO-NH-) groups. These results are induced by selfassembly of long alkyl groups introduced into DOPAM monomers, π-π stacking between benzene rings, and intermolecular hydrogen bonding between amide groups (**Figure S6**).

4) Preparation of films

The thickness of the film is controlled by the concentration of the solution and the molecular weight of the polymer. **Thick film** (**Figure 5a; Figures S47 and S56A:** The APAA polymer was dissolved in THF or $CHCl_3$ to give a polymer solution in the range of 2~10 wt%. The polymer solution filtered through a plastic membrane filter was poured onto a glass plate with a rectangular Teflon frame (3 x 7 x 1 cm or 10 x 10 x 1 cm), dried in the atmosphere for 24 h, and then dried in a vacuum oven for 24 h to make a film with a thickness of 10~330 μm. **Thin film** (**Figure S56B**: The polymer was dissolved in THF or $CHCl_3$ to make a 2.0% solution. 1.0 mL of the polymer solution filtered through a plastic membrane filter was dropped onto a silicon wafer (1 x 1 cm) and coated with a spin coater at 3000 rpm to produce a

film of 20±2 nm.

5) Production of fibers by electrospinning

Representative manufacturing processes of PD fibers, crosslinked fibers, nonwoven mats, and absorbent fiber bundles are as follows.

PD-4 fibers: PD-4 (1.0 g) in **Table 1** was dissolved in 2.9 mL (or 3.4 mL) of THF to prepare a 28 wt% (or 25 wt%) polymer solution. The polymer solution was filled into a syringe and electrospun using an electrospinning device (NanoNC Model ESR-200RD, Korea: **Figure S57A**). The polymer solution in the syringe was electrospun on a conventional stainless steel drum-type collector through a 25-gauge stainless steel needle (diameter, d=0.51 mm) under the conditions of an applied voltage of 5 kV (or 20 kV), a spinning rate of 15 mL/min, and a distance of 12 cm between the needle and the collector. As a result, PD-4 fibers with diameters of 5.0 μm and 2.5 μm, respectively, were obtained, as shown in **Figure 5b,c**. In addition, PT-0 and PH-0 were also electrospun under the same conditions to obtain (ultra)microfibers for contact angle measurements against water. The diameter of the fibers produced depends on variables such as the molecular weight of the APAA homopolymers (M_n > 80,000), the polymer concentration in the solvent (chloroform or THF), the voltage applied, the nozzle size, and the distance between the nozzle and the collector.

Aligned PD-19 fiber: As shown in **Figure 5d**, PD-19 fiber with spindle knots of 13~16 μm along the fiber (d=4~5 μm) axis were electrospun on a special aluminum collector consisting of 6 wires as shown in **Figure S57B.** Aligned fibers were collected by electrospinning through the needle under conditions of 28wt%, 10kV, 30mL/min, and 7cm.

Crosslinked PD-4 fibers: PD-4 (1.0 g) and bis(2,4,6-trimethylbenzoyl)-phenylphosphineoxide (B-PPO, photoinitiator) were dissolved in 2.9 mL of THF to prepare a 28 wt% polymer solution. The polymer solution in the syringe was electrospun through the needle onto the aluminum collector under the same conditions as the preparation of the aligned fibers. Crosslinked fibers were prepared by irradiating the fiber surface with UV (λ_{max}=365 nm, intensity=200 mW/cm^2) for 400 s while rotating (speed=3.0 m/min) the aluminum collector wound with the aligned PD-4 fibers containing the photoinitiator B-PPO in a home-made UV irradiator equipped with a mercury lamp. As shown in **Figure 5f**, the surface of the crosslinked fibers has a sponge-like morphology as well as a high nanopore density.

Nonwoven fiber mats (**Figure 5g**) were prepared by electrospinning 3-4 times on a stainless steel drum-type collector under the same conditions as PD-4 fibers (**Figure 5b,c**).

Fiber bundles (**Figure 5h**) were prepared by collecting the crosslinked PD-4 fibers produced after the UV irradiation.

6) Preparation of crosslinked PDOPAM particles by suspension polymerization

A monomer solution was prepared by dissolving 3 g (9.51 mmol) of monomer DOPAM, AIBN (0.03 g, 0.18 mmol) as initiator, and divinylbenzene (DVB, 0.066 mL, 0.46 mmol) as a crosslinking agent in benzene (15 mL). An aqueous solution prepared by dissolving suspending agent (1.2 g, Mowiol® 40) in distilled water (120 mL) was placed in a 250 mL three-necked jacket flask equipped with a mechanical stirrer, and the previously prepared monomer solution was added to the flask at a rate of 0.03 mL/s through a dropping funnel. Suspension polymerization was then carried out at 70°C for 9 h, with the monomer solution being added to the flask at a constant rate. The stirring speed during the polymerization reaction was 250 rpm. At the end of the polymerization reaction, the polymerization solution was precipitated in 1500 mL of methanol, stirred at room temperature for 3 h, and filtered to obtain crosslinked PD particles. In this step, unreacted monomer, suspending agent, water, and

benzene were dissolved in methanol and separated. The filtered crude particles were reintroduced into 200 mL of methanol, stirred at room temperature for 2 h, and filtered to obtain pure crosslinked PD particles. The particles were dried in a vacuum oven at room temperature for 24 h, yielding white spherical crosslinked PD particles (**Figure 5j**) of 110-140 μm (mean diameter=120 μm). The polymerization conversion was 88.3%.

4. Methods

1) Thermal analysis

Thermal phase transition temperature (melting temperature, T_m) of novel monomers (DOPAM, TEPAM, HEPAM) and their corresponding homopolymers was measured with a differential scanning calorimeter (DSC, TA Instrument DSC-Q100) at a heating/cooling rate of 20°C/min under a nitrogen atmosphere (**Figure S15**). The thermal stability (weight loss temperature) of the homopolymers was investigated using a thermal gravimetric analyzer (TGA, PERKIN ELMER TGA7) at a heating rate of 20°C/min in a nitrogen atmosphere (**Figure S36; Table S4)**.

2) Molecular weight measurement

Gel permeation chromatography (GPC) was performed on a Waters chromatograph system equipped with four Waters columns (Styragel HR 0.5, 2, 4 & 5) and a Waters 2414 refractive index detector. THF was used as an eluent. The number and weight average molecular weights of the polymers were calculated using polystyrene standard samples (**Table 1; Table S1**).

3) Scanning electron microscopy

The polymer solution of each sample was electrospun directly onto a silicon wafer (2.0 x 2.0 cm) to obtain microfibers. The diameter and surface morphology of the electrospun fibers (or polymer particles) were analyzed using a scanning electron microscope **(**SEM, Hitachi S-4800) at 15.0 kV after vacuum-drying the sample (**Figure 5b-f**).

4) Optical spectroscopy

UV-visible absorption spectra of polymer films were recorded on a Varian Cary-100 spectrophotometer (**Figure 4a; Figure S44**).

5) Fluorescence spectroscopy

The fluorescence properties of APAA-based polymer films were measured using fluorescence spectrometers: (1) F-900 equipped with a 450 W Xenon arc lamp (EDINBURGH Instruments Co. Ltd) for **Figure 4d**. (2) FluoroMax-4 (Horiba Scientific) for **Figure 4b,c** and **Figure S45**. To obtain the fluorescence spectra with different excitation wavelengths, the films were illuminated at different wavelengths and the fluorescence spectrum was scanned between λ_{ex}+20 nm and 700 nm. The photoluminescence excitation (PLE) spectra were collected by monitoring the fluorescence intensity at different emission wavelengths (440, 480, 520, and 530 nm) while scanning the excitation wavelengths from $\lambda_{em}/2+15$ nm to $\lambda_{em}/2-15$ nm and dividing the signal to the detector responsibility at each excitation wavelength. For **Figure 4b**, the slit widths for excitation and emission were kept constant for different excitation wavelengths. For **Figure 4c** and **Figure S45**, which focus on the peak position, the slit widths of excitation and emission were adjusted to obtain the high quality spectra.

6) Dynamic fluorescence spectroscopy

The lifetime (or decay time) of the blue fluorescence emitted from the polymer films was measured using a time-resolved and integrated photoluminescence/Raman spectrometer with the following conditions: detector, Streakscope C4334 (Hamamatsu, Japan); excitation light source, picosecond light

pulser (374nm, 65ps pulse width, 50mW peak power); DG535 four-channel digital delay/pulse generator; 600kHz repetition rate; 20ns time scale (**Figure S50; Table S7**).

7) Transmission electron microscopy

TEM was performed at 300 kV using a JEOL-JEM 3010. Ultrathin sectioning of the samples was performed on an ultramicrotome using a Leica EM UC7. Prior to ultathin sectioning, the samples were aligned by annealing at 250°C for 2 h. Thin sections of the samples were transferred to a carbon-coated copper grid and stained with R_uO_4 vapor. Data were analyzed using Digital Micrograph software (**Figures S27 and S28**).

8) BET surface area measurement

To determine the surface area of novel mesoporous APAA polymers, their benzene vapor adsorption and desorption isotherms were recorded on BELSORP-max. Prior to measurement, all polymer samples were annealed at 200°C for 1h to activate polymer chains with hexagonal cylindrical nanostructures based on DSC and XRD results, and then 110 to 330 mg of polymer powder samples were degassed under vacuum at 120°C for 1 h. The surface area of each sample was calculated by the Brunauer-Emmett-Teller (BET) method[66] using adsorption data at relative pressures (P/P_o) ranging from 0.01 to 0.5 at standard temperature and pressure (STP, 25°C, 1 atm). The cross-sectional area[67] of the adsorbed benzene molecule is 0.430 nm^2. The total pore volume is the amount of benzene molecules adsorbed in the monolayer at a P/P_o of 0.90 (**Figures S29-S31**).

9) Hydrogen gas storage

The adsorption of hydrogen gas at high pressures up to 90 bar was measured volumetrically[68] using a pressure-composition isotherm (PCT, BELSORP-HP, BEL JAPAN). Hydrogen gas of 99.9999% purity was used for all H_2 sorption measurements. For the PCT measurement, the system was calibrated with $LaNi_5$ at room temperature and with activated carbon (surface area ~3000 m^2/g) at 77 K, respectively. PD-4 film prepared with a thickness of 100 μm was cut into 2.0 x 1.0 cm and then the films of 150±10 mg (or PD-4 powder, 150 ±10 mg) were degassed before measurement and heated at 120 ºC until a pressure of 4~8 x 10^{-7} mbar was reached. The desired hydrogen pressure was then introduced into a thermostatic chamber and once equilibrium was reached, the gas was allowed to expand in a sample holder. The measured pressure drop was caused by gas expansion within the sample holder and hydrogen adsorption into the sample. If no further pressure change was observed, a further 300 s was allowed to elapse until thermal equilibrium was reached (**Figures S32 and S33**).

10) Mechanical properties

The mechanical properties (**ASTM D882**) of APAA polymer films (thickness, 60~65 μm) cast from 5% chloroform solution were measured at a crosshead speed of 10 mm/min using an Instron Tensile Tester (Model 4201). For each sample, mechanical tests were performed on three specimens (60 mm x 5 mm). Modulus (MegaPascal, MPa), ultimate stress (MPa), stress at break (MPa), and strain at break (%) are the average of the mechanical properties obtained from the stress-strain curves of the three specimens (**Figures S52 and S53; Table S8**).

11) Water contact angle of APAA polymers

The contact angle of a 7.0 mg water droplet on eletrospun fibers and films coated on a silicon wafer was measured using a KRUSS EasyDrop FM 40 (**Figure 5I; Figures S55 and S56**).

5. Characterization of physicochemical properties of mesoporous APAA polymers

1) Determination of polymer stereoregularity by ^{13}C NMR spectroscopy

The stereoregularity of new APAA polymers (PD, PT, PH) was analyzed by ^{13}C NMR spectroscopy (500 MHz, Bruker Avance III HD 500). All ^{13}C NMR spectra were measured under the following conditions: solvent=THF-d_8 (or $CDCl_3$), room temperature, number of scans=16,000 (~15 hours) and d1=2 seconds.

Stereoregularity model for PD: Dyad tacticity is defined as the fraction of pairs of adjacent repeating units that are isotactic or syndiotactic to one another. As shown in the polymer chain below, the fractions of isotactic and syndiotactic dyads are commonly referred to as meso (m) and racemic (r) dyads. The triad tacticity describes isotactic, syndiotactic, and heterotactic (so-called atactic), whose fractions are designated as (mm), (rr) and (mr), respectively. The atactic triad (mr) has no stereoregularity between the repeating units[61].

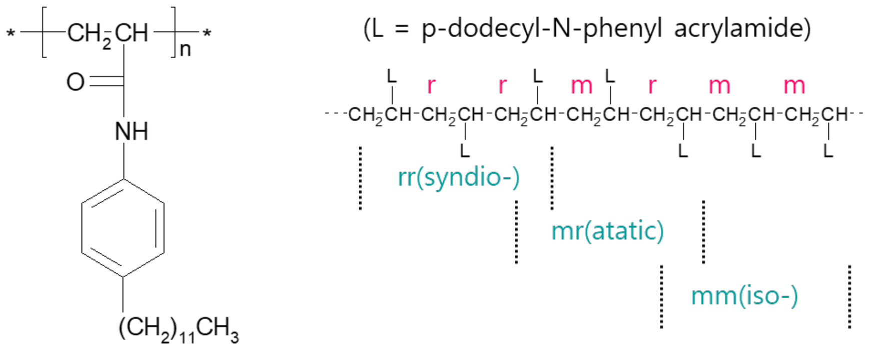

Tacticity, the ratio of [syndiotactic] to [isotactic], was determined by quantifying the relative proportions of racemic (rr) to meso (mm) triads in the solution phase ^{13}C NMR (Bruker Avance III HD 500) spectrum (**Figure S11)**. The quantitative area ratio of rr to mm was calculated using commercially available Origin or Igor software **(Figures S12 to S14**). The methyn (or alpha, α) carbon* (-CH_2-*CH-L) peak in the backbone chain of PD depends on the stereoregularity of the side chain (L) in adjacent repeat units. For example, the α-carbon peak in a typical polyacrylamide backbone chain is split into three peaks appearing at 43.20, 42.82, and 42.38 ppm, respectively, which are assigned in the ^{13}C NMR literature as syndiotactic (rr), atactic (mr), and isotactic (mm) triads[62]. When THF-d_8 was used as the NMR solvent, the methyn peak of the new APAA polymers had a slightly higher value (0.2 ppm) than that of the polyacrylamide, the reference sample, due to the benzene group substituted on the amide linkage. With $CDCl_3$, the methyn peak shifted 1.0 ppm higher than with THF-d_8. Unfortunately, the atactic triad was indistinguishable from the syndiotactic and isotactic triads even in the 600 or 700 MHz ^{13}C NMR spectra.

^{13}C CP MAS solid state NMR spectrum of DOPAM monomer and APAA polymers (**Figures S7 to S10**): Solid state NMR spectra were recorded on a Bruker AV 600 MHz spectrometer using a standard Bruker Magic Angle Spinning (MAS) probe with 4 mm (o.d.) zirconia rotors: The magic angle was adjusted by maximizing the number and amplitude of the rotational echo signals observed in the 79 Br MAS FID signal from KBR. The ^{13}C NMR transmitter frequency is 150.90 MHz. The solid state ^{13}C NMR spectra were obtained using the cross polarization (CP) MAS technique with the ninety degree pulse of ^1H with a pulse width of 4.2 μs. The CP contact time was 2 ms. The decoupling frequency was 32 kHz. The Mas sample spin rate was 11 kHz. The cycle delay between scans was 2 s.

2) X-ray diffractometry

Synchrotron X-ray (**SAXS**) measurements were performed at the 9A beamline at the Pohang Accelerator Laboratory (PAL) in Korea to investigate the morphological structures of APAA polymers. The wavelength of the X-ray was 1.119 Å and the distance from the sample to the detector was 2.6 m.

The exposure time for each image was 1 s. The SAXS patterns were recorded by a two-dimensional X-ray detector using a CCD (PI-SCX4300-165/2, Princeton Instrument). The SAXS patterns were measured after holding the polymer samples for 10 min at predetermined temperatures for both heating from 25 to 225℃ and cooling from 225 to 25℃ to minimize the temperature influence (**Figure 2b; Figures S19 and S20**). For continuous heating measurements, the sample was continuously heated from 25 to 280℃ at the rate of 5.0℃ /min, with spectra acquired every ~1.5℃ (**Figure 3a; Figures S35, S39, S40**). One dimensional **WAXD** patterns were obtained using an X-ray radiation generator (CuK_{α}, λ=1.5417 Å) coupled to a diffractometer (Rigaku, Japan) at room temperature or -70 ^{o}C. Silicon crystal powder was used as an internal reference showing a diffraction ring at 2θ = 28.466° (**Figures S18 and S21**).

3) DSC experiments with isothermal annealing near the melting temperature

We performed multi-step DSC experiments consisting of three cycles of melting at 260°C and isothermal annealing at 220°C, 225°C, and 230°C (**Figure S37**). These cycles were performed in both ascending and descending sequences, with DSC curves measured after each annealing step (**Figures S38 and S41**). The identified T_c^1 and T_c^2 and their enthalpies are summarized in **Table S5**. Two phase transition peaks were detected at all annealing temperatures and T_c^1 and T_c^2 remained consistent regardless of the annealing cycle history. It was also observed that higher annealing temperatures resulted in higher T_c^1 and T_c^2.

4) Temperature-dependent IR spectroscopy

The infrared reflection absorption spectrum (IRRAS) was measured at a spectral resolution of 4 cm^{-1} using a Bruker (Karlsruhe, Germany) VERTEX 80v FT-IR spectrometer equipped with a liquid nitrogen cooled MCT detector. The IRRAS measurements were performed using a Bruker A513 reflection attachment with a heating block attachment and a p-polarized infrared beam at an angle of incidence of 79°. To ensure a high signal-to-noise ratio, 1024 scans were co-added for each measurement. Both sample and source compartments were evacuated to 1 mbar. The temperature-dependent IRRAS of PD-14, PT-8, and PH-2 was measured in 5 ^{o}C increments in the range of 25~280°C (**Figure 3c; Figures S16 and S17**). To measure IRRAS, the polymer films were prepared by casting their chloroform solutions on a Pt-coated silicon wafer and kept in a vacuum drying oven at room temperature for 12 h. In order to study the temperature dependence of the polymer suprastructures (lamellae and hexagonal cylinder), the synchronous and asynchronous 2D correlation spectra (**Figure 3f; Figure S43**) were calculated using the algorithm based on the numerical method developed by Noda and executed in MATLAB. PCA (**Figure 3d,e; Figure S42**) was carried out using PLS_Toolbox Ver. 4.2 (Eigenvector research, Inc., Wenatchee, WA) for MATLAB[63,64].

5) Molecular mechanics simulation

To gain insight into the structural changes during the phase transition from lamellar to hexagonal cylindrical nanostructures observed in the XRD and DSC results, and to predict the three-dimensional (3D) nanostructures of APAA polymers (**Figures 1a,c and 2a; Figures S22–S26**), we performed molecular mechanics simulations. These simulations, conducted using nanoscale molecular dynamics (NAMD)[33] based on the optimized potentials for liquid simulations (OPLS) force field[65], employed with a periodic boundary condition (PBC) and a conjugated gradient method for structural optimization.

6) Fluorescence microscopy

In a dark room, films, fibers, or crosslinked polymer microparticles were placed on a microscope slide and then their red, green, and blue (RGB) images (**Figure S46**) were observed using a fluorescence microscope (OLYMPUS BX51) in red (U-MWG2 filter; excitation: 510-550 nm; emission:

>590 nm), green (U-MWB2 filter; excitation: 460-490 nm; emission: >520 nm), and blue (U-MWU2 filter; excitation: 330-385 nm; emission: >420 nm). The images emitted by the sample were captured using a CCD camera (DP 70) with an exposure time of 300 ms for film and fibers, but 400 ms for microparticles.

After printing a thumbprint on PD-4 transparent film (60 μm), a photograph (**left side, Figure S51**) was taken using a Sony Alpha 6000 digital camera equipped with a macrolens (Sony SEL30M35) while exposed to a 365nm portable UV lamp (2.5mW/cm^2). The contrast of the photograph was increased by 40% using the PowerPoint program (Microsoft Co. Ltd.). Enlarged RGB images (**right side**) of the thumbprint were obtained using the fluorescence microscope with an exposure time of 555 ms. The RGB images were corrected for noise using standard noise reduction software. The measurement procedure is the same as described for the RGB fluorescence image of the film.

7) Quantum yield measurements

The photoluminescence quantum yield (QY) of APAA polymer films was measured using an optical setup equipped with an integrating sphere (IS200-4, Thorlabs) and a spectrophotometer (HR2000+CG-UV-NIR, Ocean Optics). The sample was illuminated with a 365 nm LED (M3655FP, Thorlabs). A 400 nm long pass filter (FELH400, Thorlabs) was used to block the excitation beam. The spectrophotometer was calibrated using a calibrated silicon photodetector (UV-818, Newport). The optical setup for QY measurements is shown in **Figure S48**[69]**.** The calculation formula for QY is as follows. The resulting QY is given in **Table S6.**

Sequence of experimental measurements:

1) Blank without long-pass filter (P1)
2) Blank with long-pass filter (P2)
3) Sample without long-pass filter (P3)
4) Sample with long-pass filter (P4)

$$QY = \frac{\#\ of\ emitted\ photons}{\#\ of\ absorbed\ photons} = \frac{P_e/E_e}{P_a/E_a}$$

$$Emission\ (P_e)\ =\ P4 - P2, \quad Absorption\ (P_a) =\ P1 - P1 + P4 - P2$$

$$E_e = \frac{hc}{\lambda_e}, \quad E_a = \frac{hc}{\lambda_a}$$

8) Measurement of VOC absorption properties of crosslinked polymers

Solvent absorbency was measured by placing 0.2 g of crosslinked polymer microparticles or 30±3 mg of crosslinked fiber bundle in a beaker filled with 10 cc of VOC (benzene, chloroform, THF, toluene, n-octane, or DMF), and retrieving them after 30 min for the microparticles or 10 min for the bundle. The polymer gel swollen in the solvent was filtered through a 100 mesh stainless steel sieve for 10 min and weighed on an electric balance. Absorbency (swelling ratio) for a given solvent was calculated by dividing the weight of the swollen polymer gels by the weight of the dried polymer particles (**ASTM F726-81**).

Absorption rate was measured using an optical microscope (Keyence VH-Z100UR). The absorption rate is defined as the time taken for the microparticles (**Figure S54**) to reach its maximum absorption capacity for a given solvent. In other words, the absorption rate was determined as the time taken for the spherical vertex formed inside the swollen polymer gel particles to disappear upon absorption of a

given solvent (see **Movies S1 and S2 in Supporting Information**).

The measurement procedure is as follows:

(i) 10mg of crosslinked PD spherical particles are evenly distributed on a transparent glass plate.

(ii) Cover the particles with a cover glass.

(iii) Drop 2 mL of benzene on the edge of the cover glass and take photographs, observing the expansion of the particles over time.

Summary of results

Solvent absorbency: PD-4 fiber bundles absorbed chloroform at 20.7 times their dry weight, followed by THF at 15.5 times, toluene at 13.4 times, benzene at 12.5 times (**Figure 5h,i**), and n-octane at 4.5 times. Similarly, crosslinked PD microparticles (**Figure 5j**) displayed notable VOC absorbency, absorbing 16.3 times for chloroform, 10.5 times for benzene, and 3.4 times for n-octane.

Absorption rate: Remarkably, absorption saturation was reached within 10 s. Fiber bundles showed faster absorption than microparticles; both PD-4 and PT-0 fiber bundles reached saturation for benzene in less than 5 s. For PD microparticles, chloroform and benzene reached absorption saturation in only 7 and 10 s, respectively. In contrast, the absorption rate of n-octane was much slower, taking 15 min to reach saturation. This sluggish absorption can be attributed to weak van der Waals interactions compared to the strong π-π interactions between benzene and benzyl groups or polar interactions between chloroform and amide groups in APAA polymers **(Movies S1 and S2)**.

6. Legends of Movies S1 and S2 in a separate file

Movie S1 (Absorption rate of fiber bundles for benzene): PD-4 fiber and PT-0 fiber bundles selectively separate benzene (12.5 times) mixed in water within 5 seconds. Benzene was colored with a methyl red indicator dissolved in methanol.

Movie S2 (Absorption rate of crosslinked microparticles for chloroform): It takes about 7 seconds for the crosslinked polymer microparticles to absorb the maximum amount of chloroform (16.3 times).

7. Figures S1 to S56

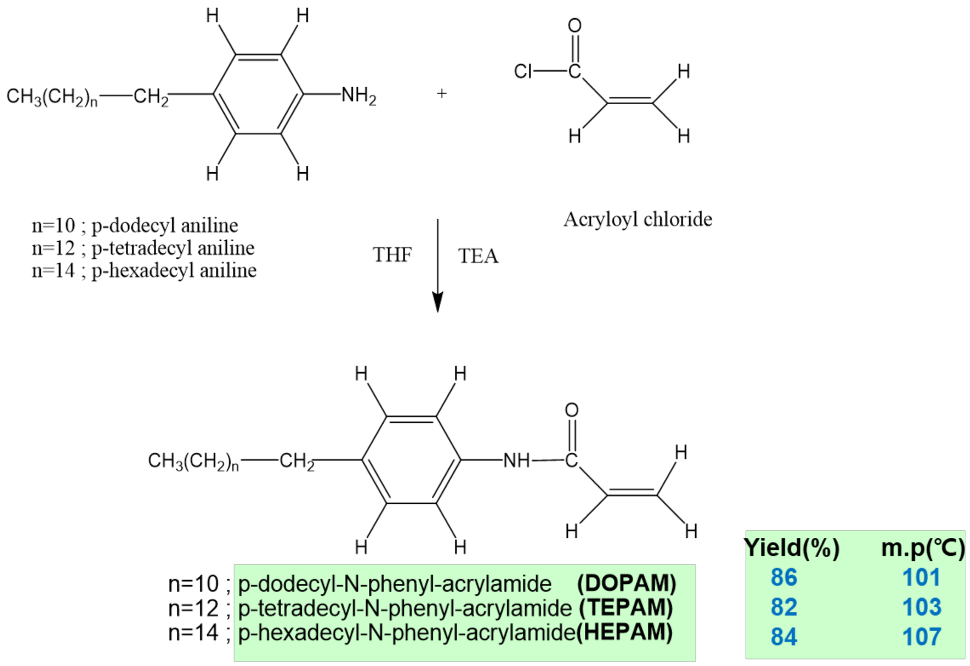

Figure S1. Synthesis of novel p-alkyl-N-phenyl-acrylamides (APAA) as vinyl monomers.

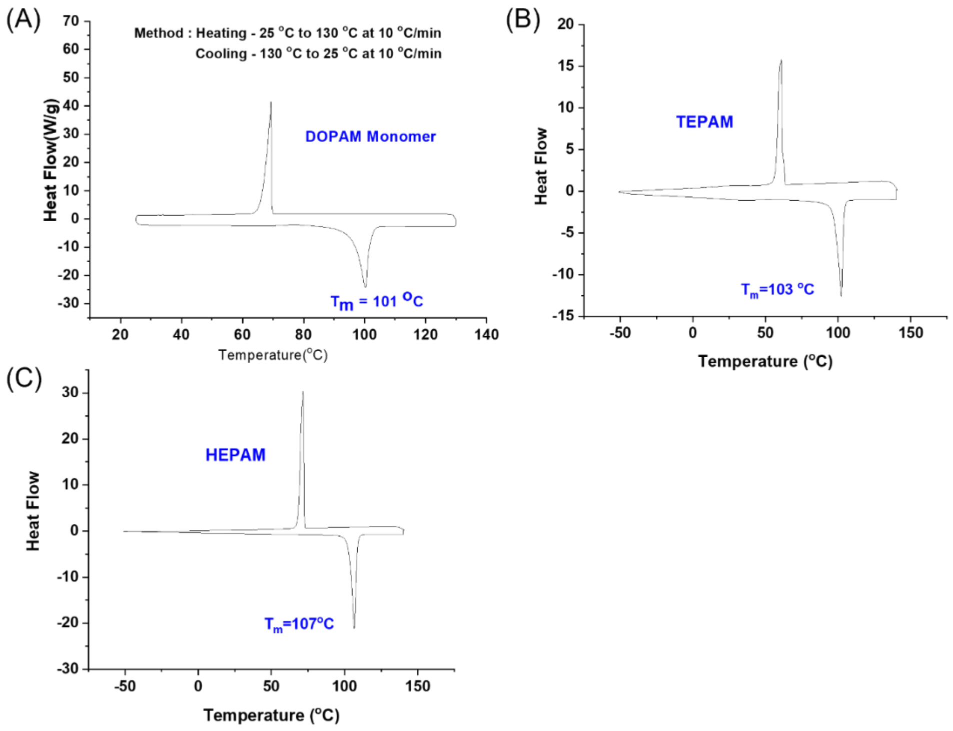

Figure S2. DSC thermograms of APAA monomers: (A) DOPAM, (B) TEPAM, and (C) PHEPAM.

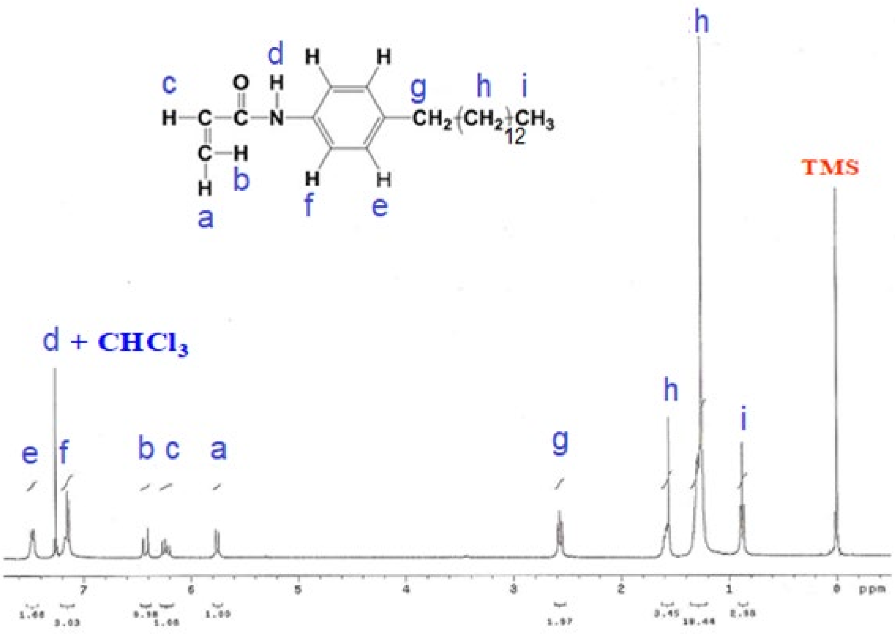

Figure S3. ^{1}H-NMR spectrum of representative crystalline TEPAM monomer.

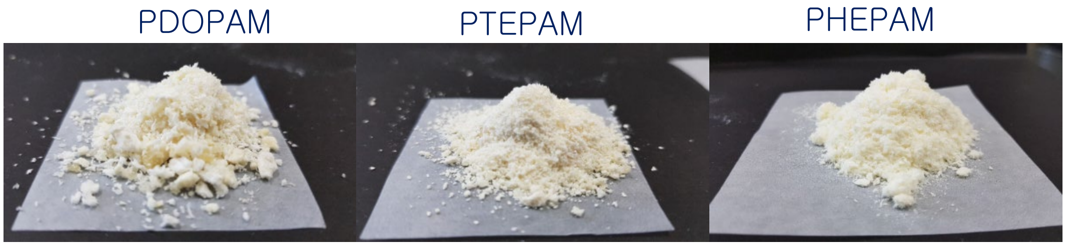

Figure S4. 10 g scale synthesis of APAA polymers.

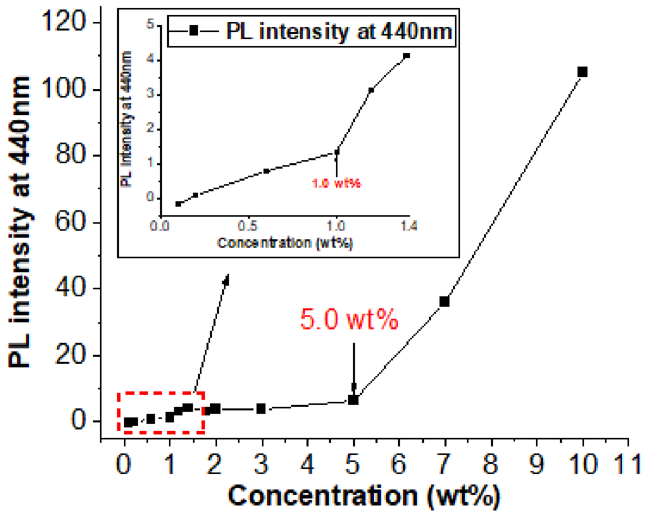

Figure S5. Concentration dependent photoluminescence intensity of DOPAM solutions at 440 nm.

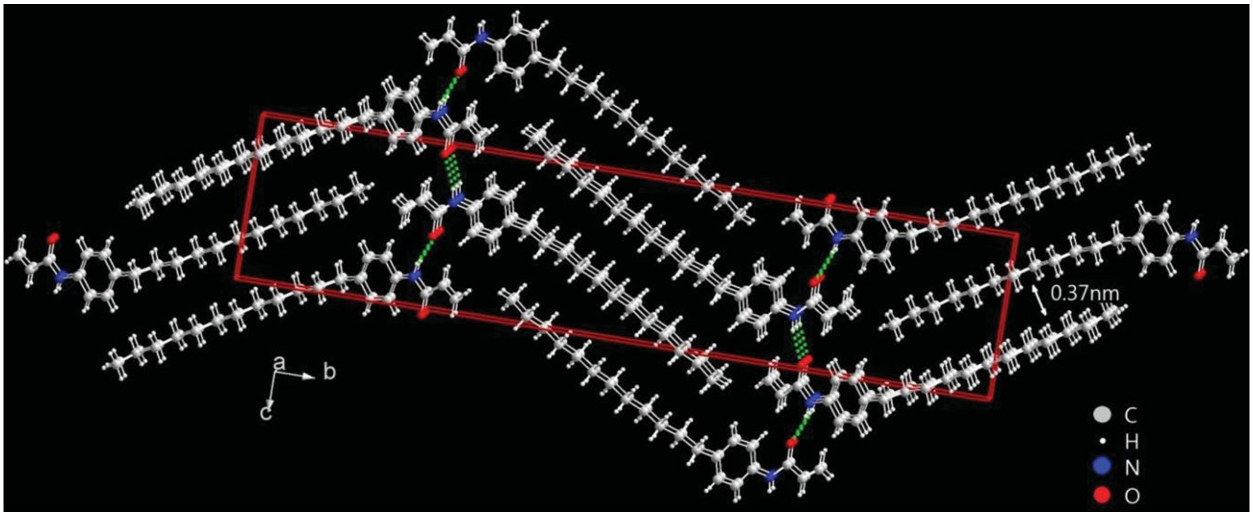

Figure S6. Three-dimensional structure of the single crystal of DOPAM monomer. The dotted green line indicates the intermolecular hydrogen bond (bond distance: 2.046±0.010 angstrom) between amide groups in each layer. The distance between benzene groups (3.653±0.017 angstrom) in the same layer is the same as that of the well known π-π interaction.[24]

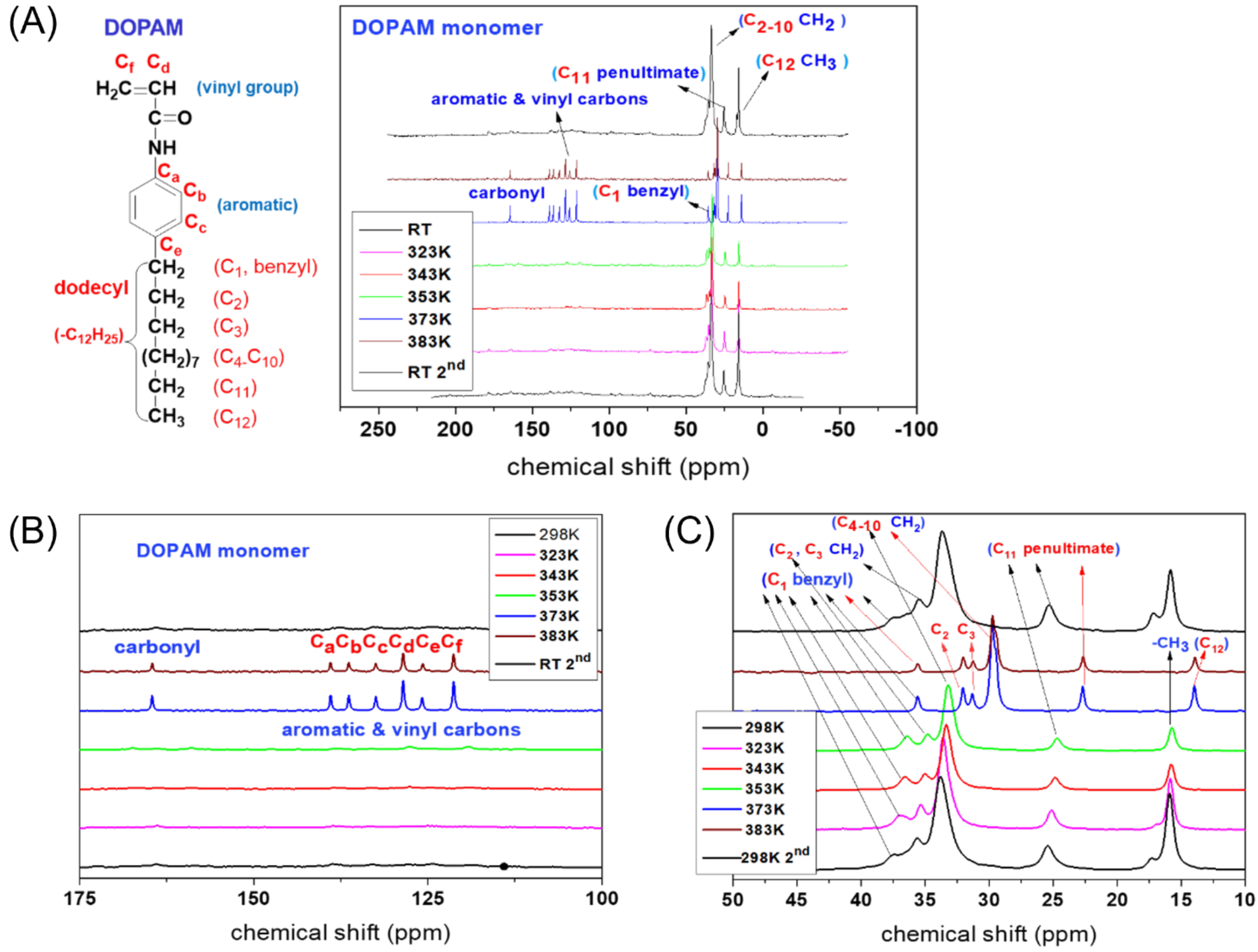

Figure S7. ^{13}C CP MAS solid state NMR spectrum (A) of crystalline DOPAM monomer as a function of temperature and magnified spectra between 175~100 (C) and 50~10 ppm (C).

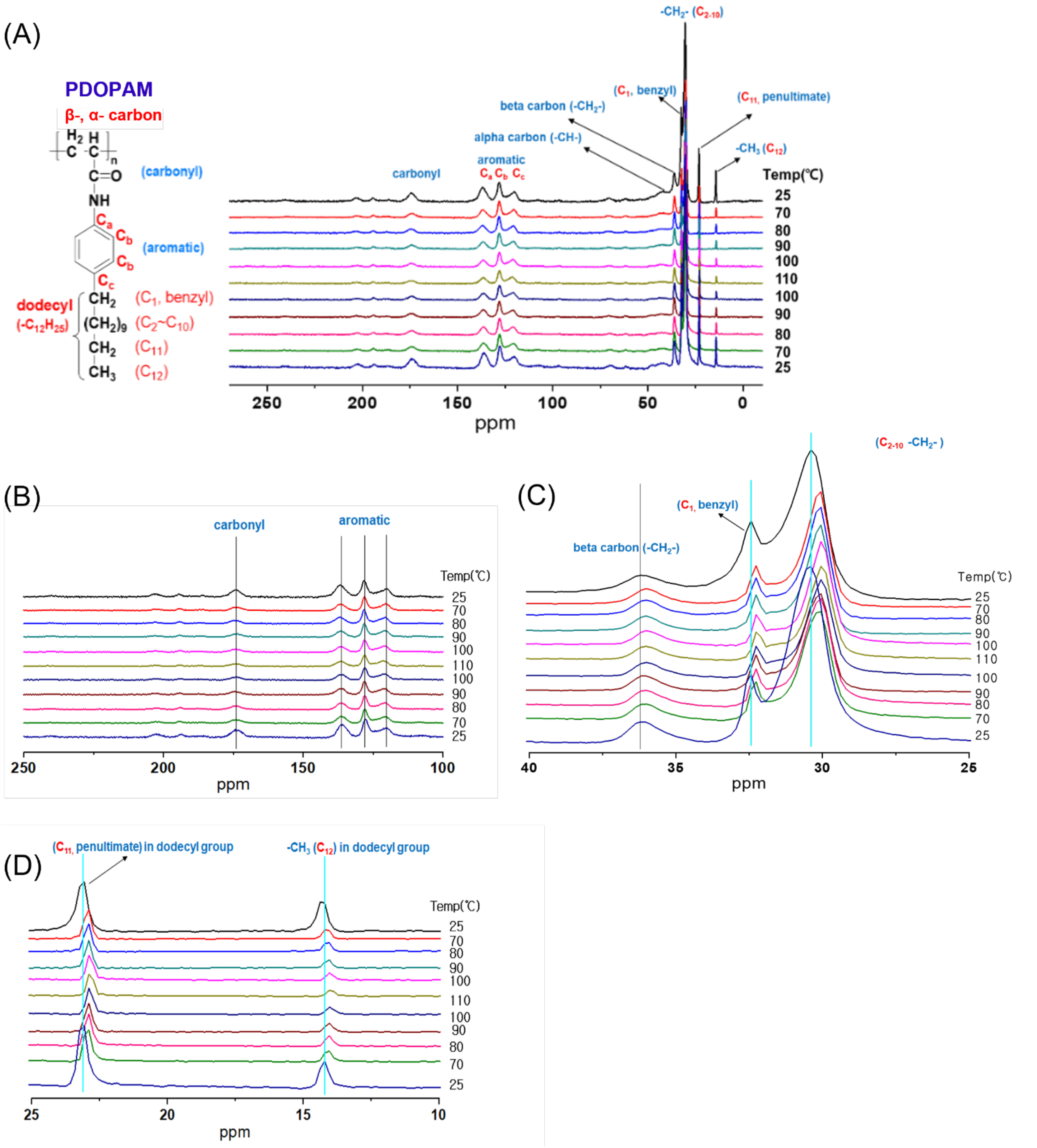

Figure S8. ^{13}C CP MAS solid state NMR spectra of stereocontrolled (A) PD-4 obtained during successive heating and cooling cycles, and magnified spectra between (B) 100-250 ppm, (C) 25-40 ppm and (D) 10-25 ppm.

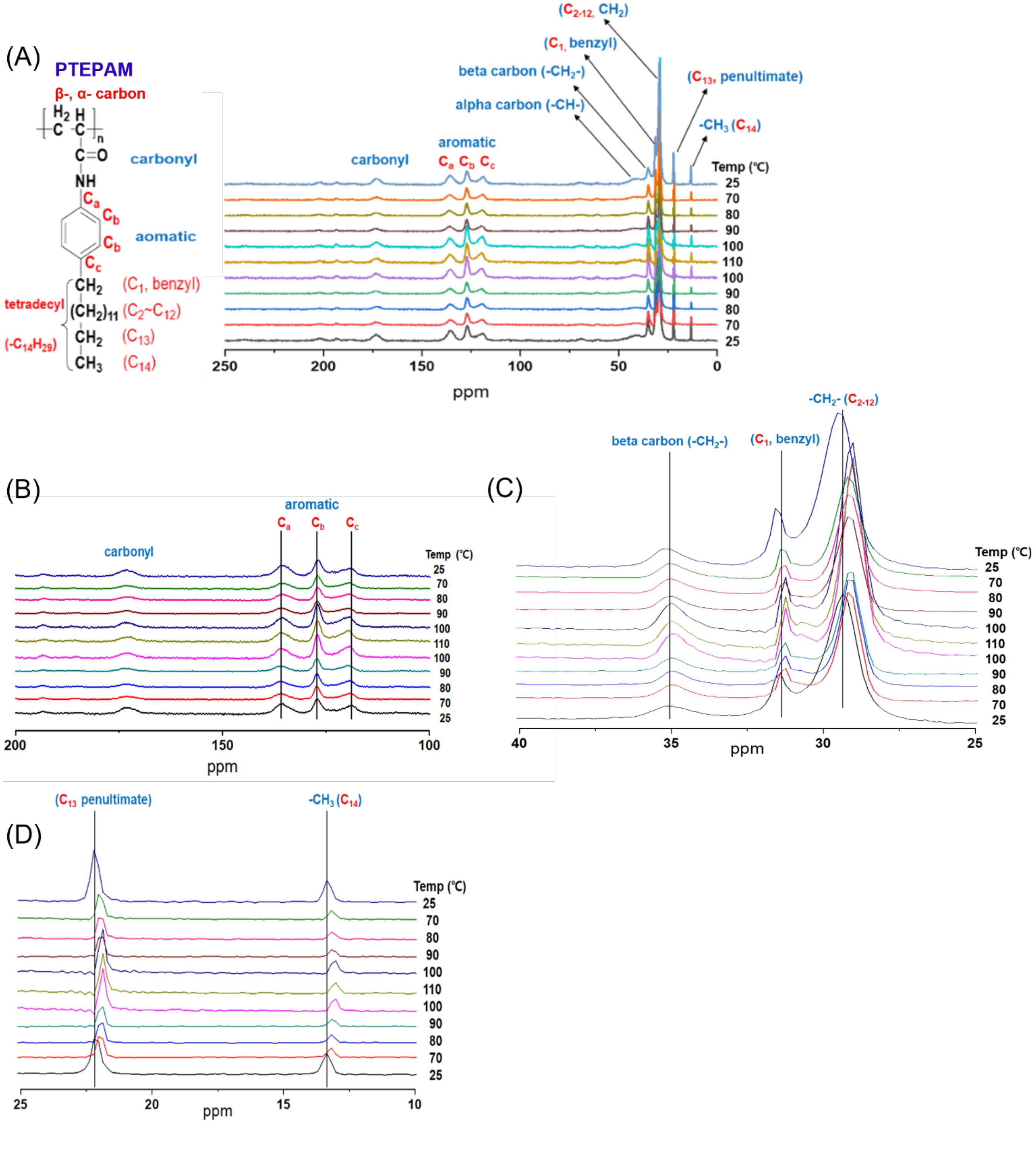

Figure S9. ^{13}C CP MAS solid state NMR spectra of stereocontrolled (A) PT-0 during successive heating and cooling cycles and magnified spectra for (B) 100-200 ppm, (C) 25-40 ppm and (D) 10-25 ppm.

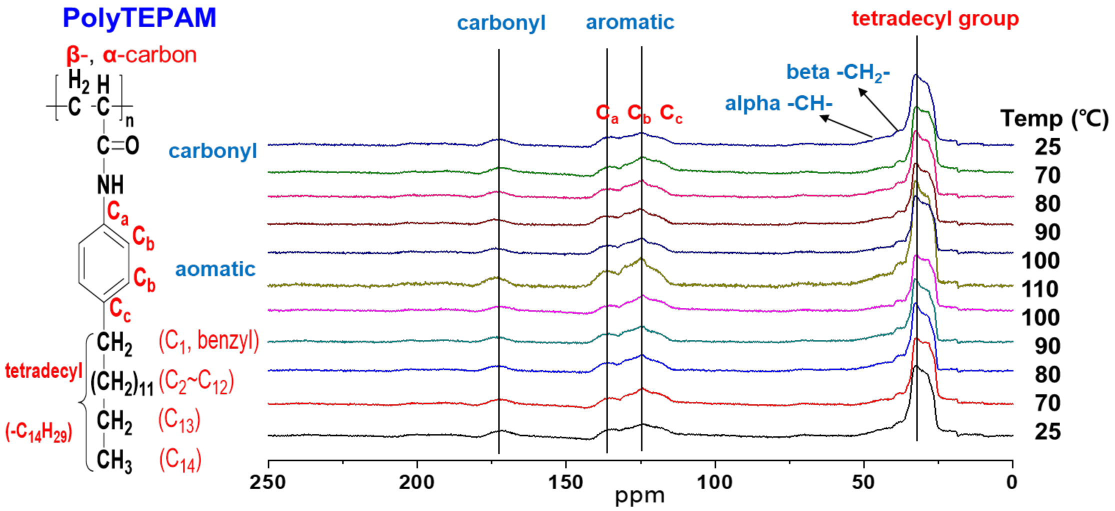

Figure S10. ^{13}C CP MAS solid state NMR spectra of non-stereocontrolled PT-6 obtained during successive heating and cooling cycles.

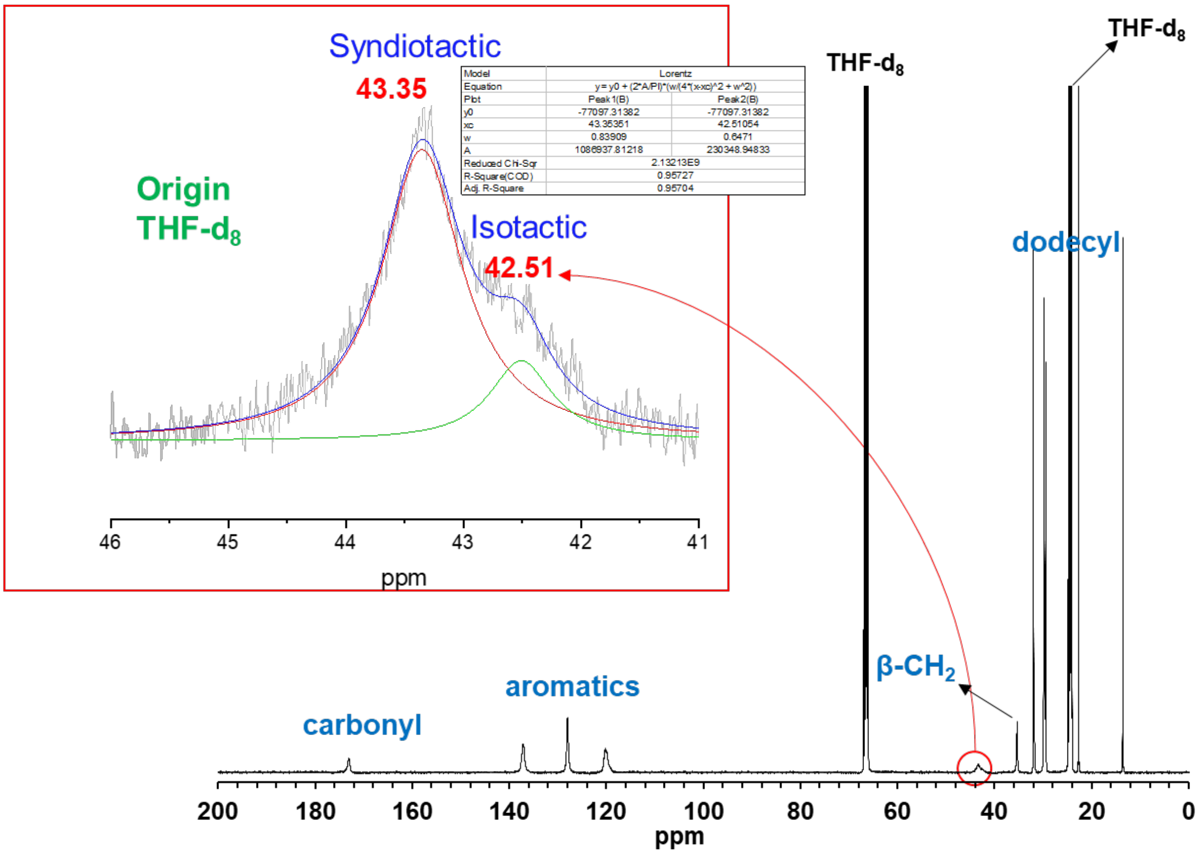

Figure S11. ^{13}C NMR (500 MHz) spectrum of representative PD-4 in d_8-THF.

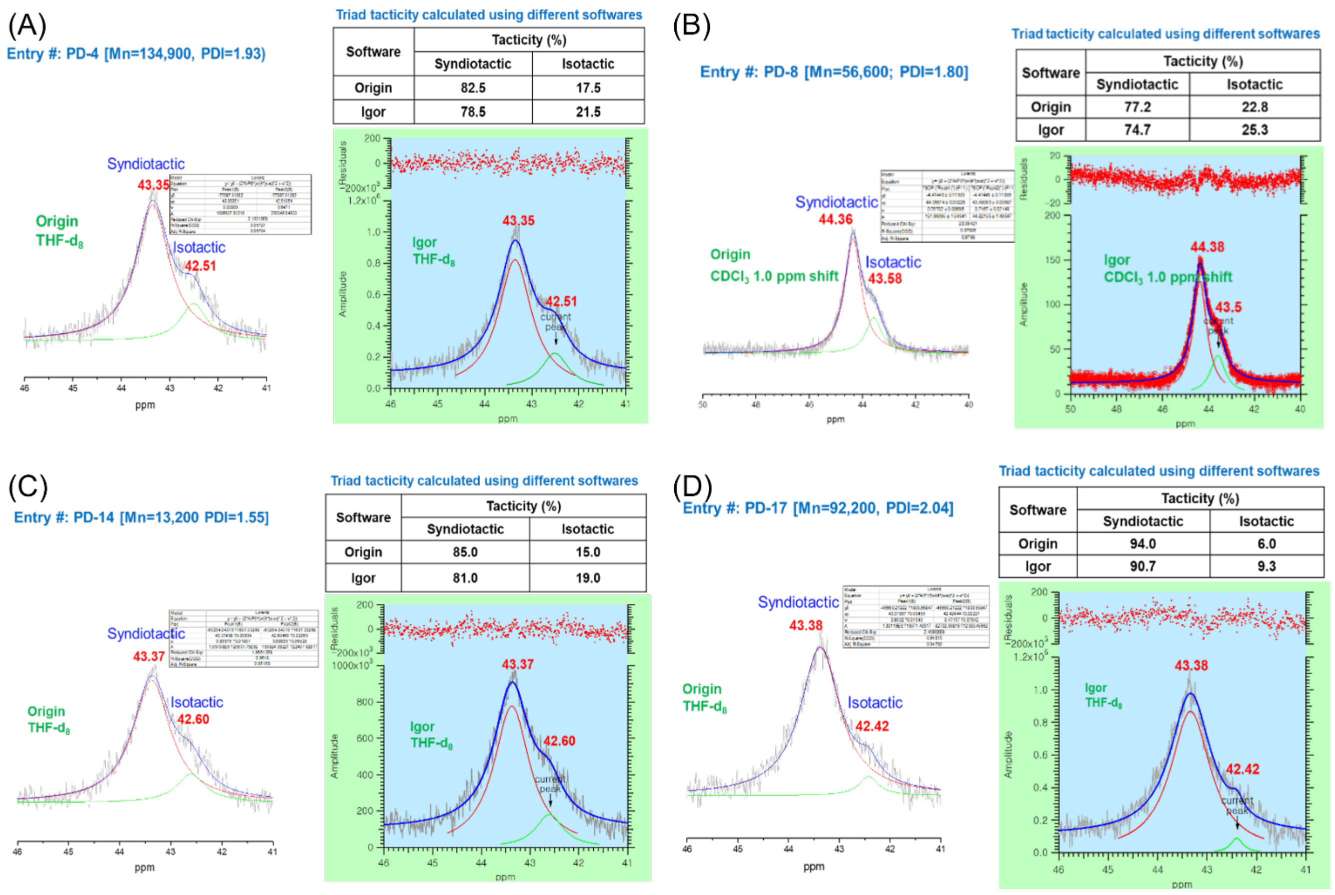

Figure S12. Triad tacticity of (A) PD-4, (B) PD-8, (C) PD-14 and (D) PD-17 calculated using Origin or Igor software. The (rr) and (mm) triads appear at 43.36±0.02 and 42.50±0.1 ppm in THF-d_8, respectively. The use of $CDCl_3$ (B) as solvent shifts the triad peaks up by 1.0 ppm.

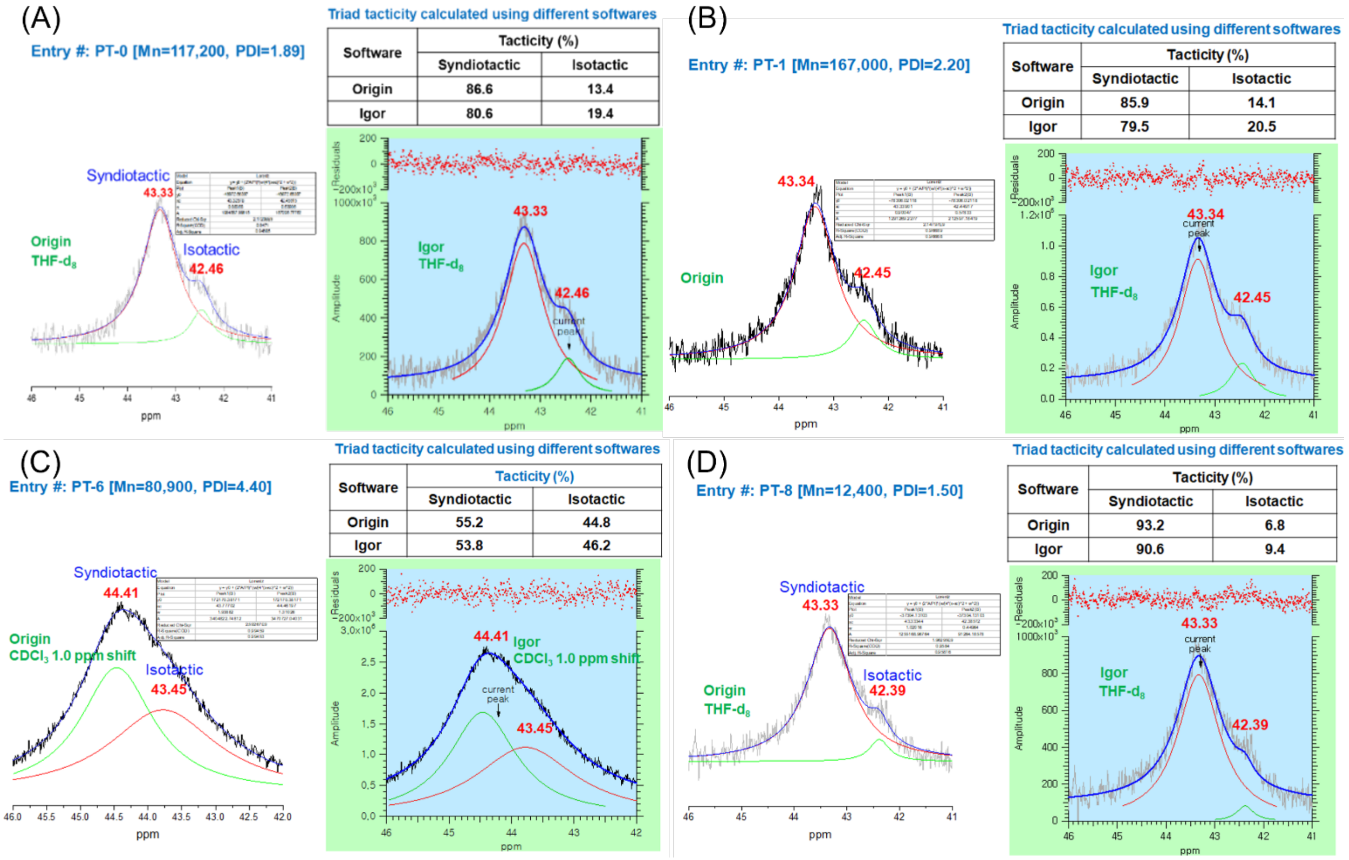

Figure S13. Triad tacticity of (A) PT-0, (B) PT-1, (C) PT-6 and (D) PT-8 calculated using Origin or Igor software. The (rr) and (mm) triads appear at 43.36±0.02 and 42.50±0.1 ppm in THF-d_8, respectively. The use of $CDCl_3$ (C) as solvent shifts the triad peaks up by 1.0 ppm.

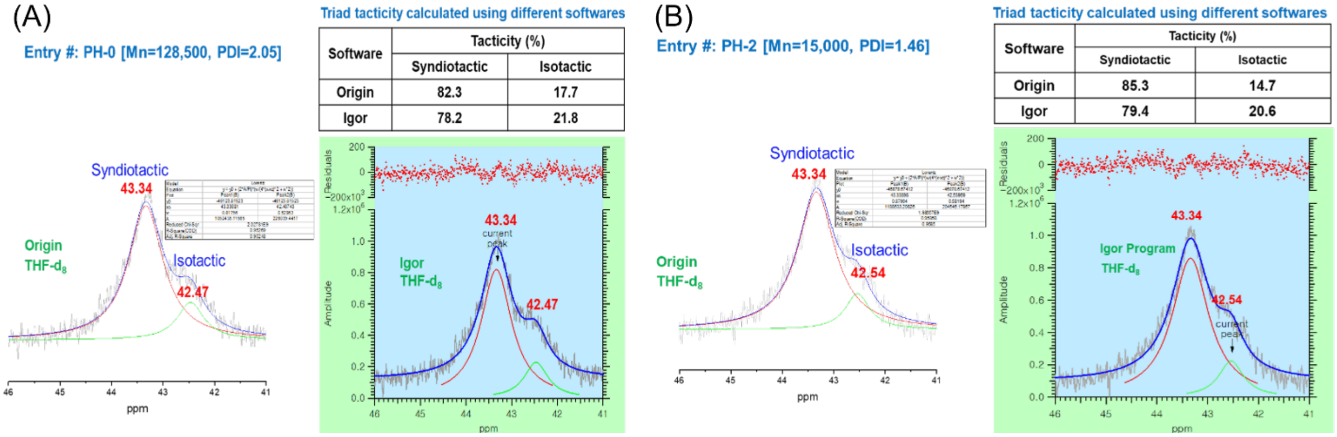

Figure S14. Triad tacticities of (A) PH-0 and (B) PH-2 calculated using Origin or Igor software. The (rr) and (mm) triads appear at 43.36±0.02 and 42.50±0.1 ppm in THF-d_8, respectively.

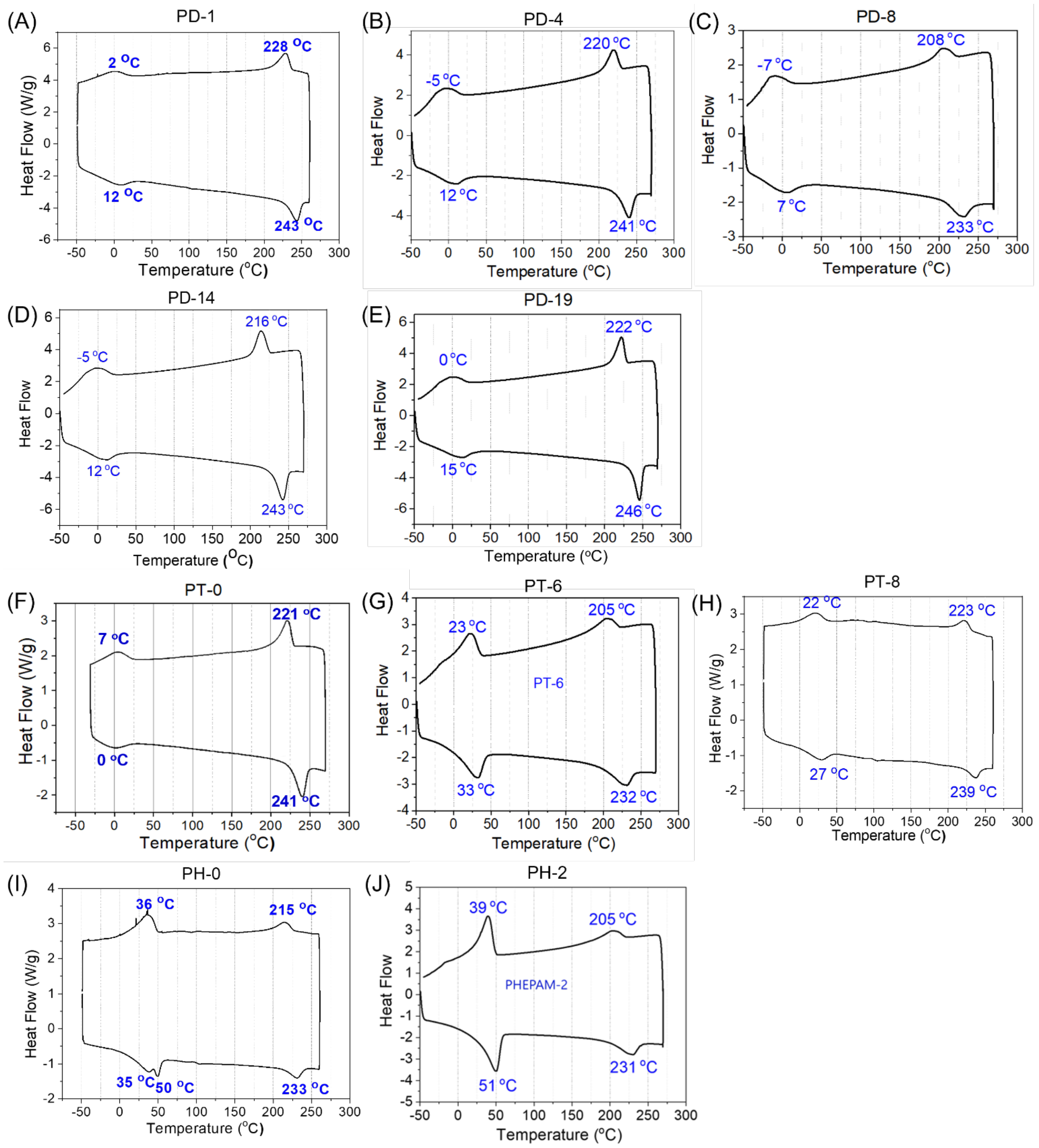

Figure S15. DSC thermograms of representative mesoporous APAA polymers: (A-E) PDOPAM, (F-H) PTEPAM, and (I, J) PHEPAM.

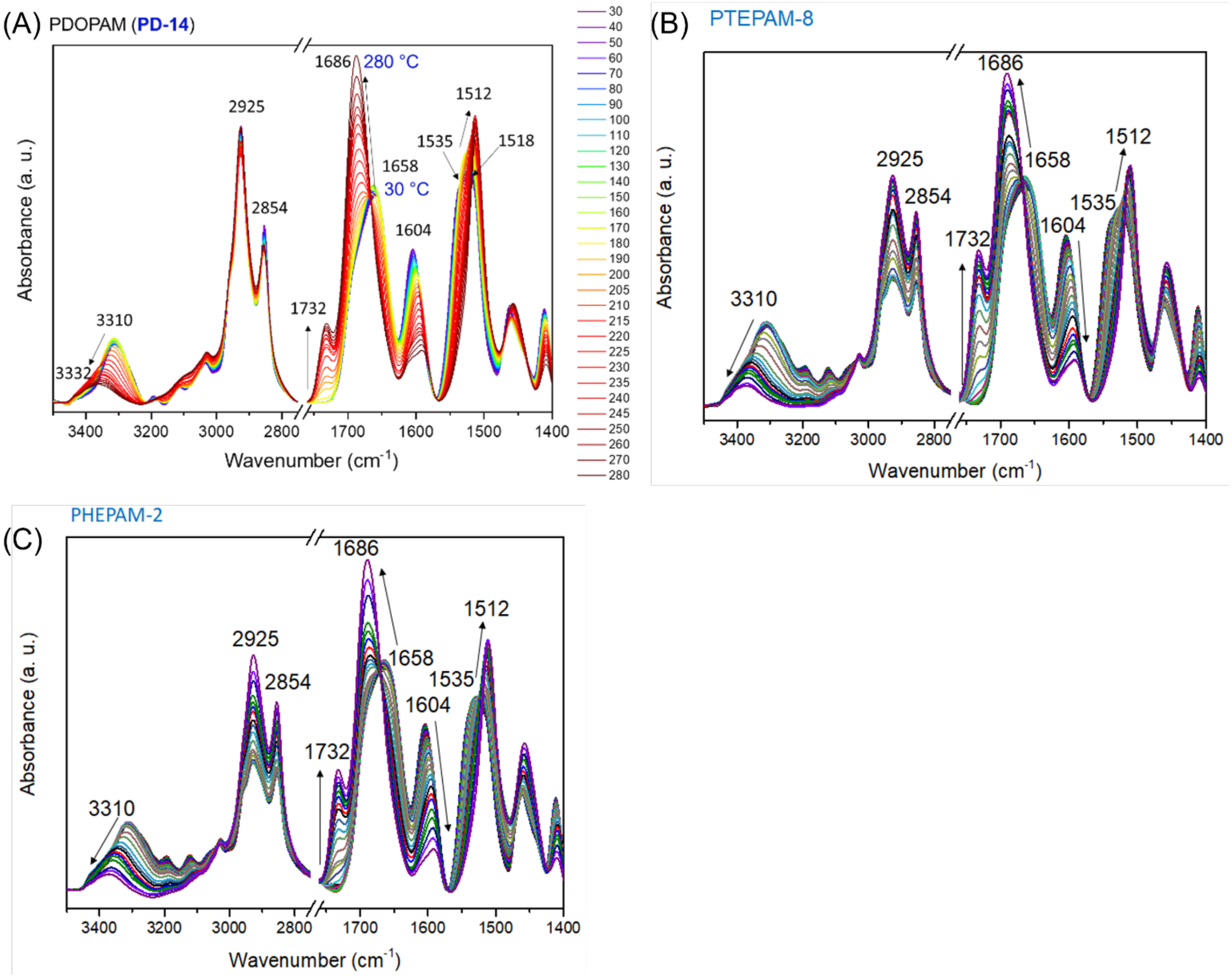

Figure S16. Temperature-dependent IRRAS of (A) PD-14, (B) PT-8 and (C) PH-2 obtained during the heating process from 30 to 280°C. The inset arrow indicates the direction of temperature increase.

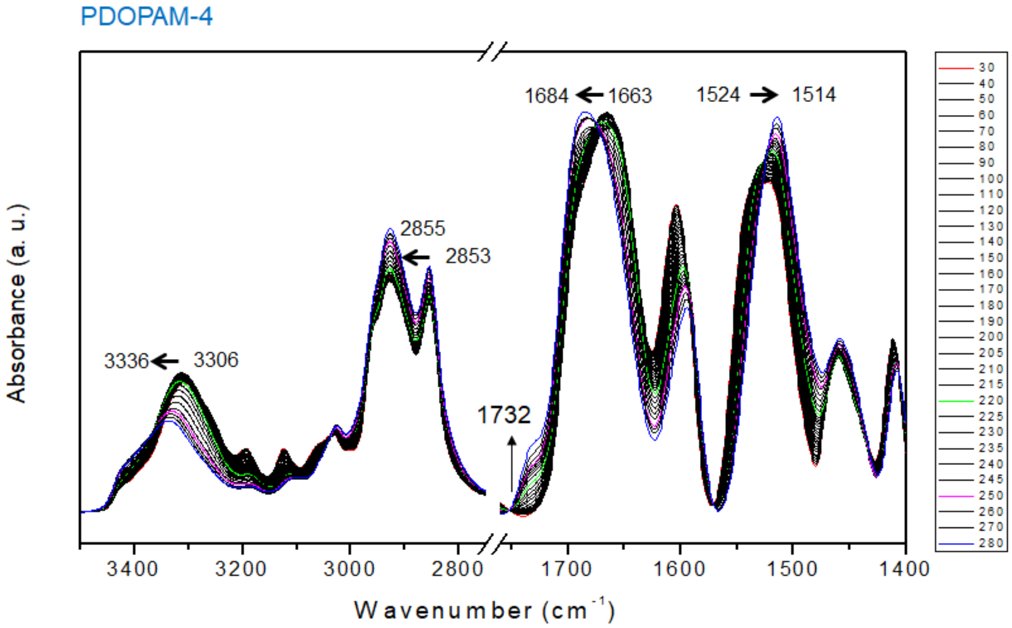

Figure S17. Temperature-dependent IRRAS of PD-4 with high molecular weight (M_n=134,900) obtained during the heating process from 30 to 280°C. The inset arrow indicates the direction of temperature increase.

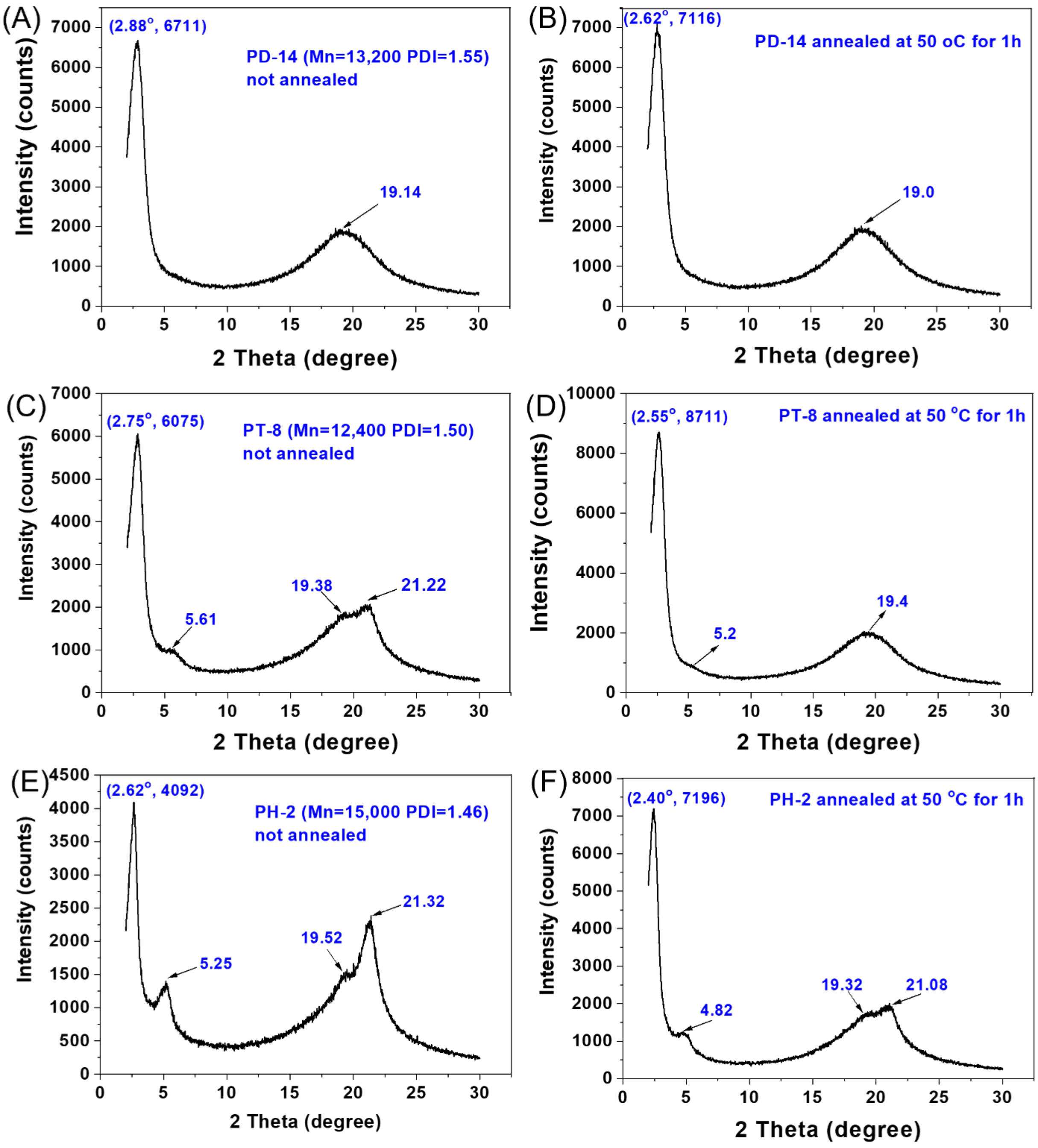

Figure S18. WAXD pattern of mesoporous APAA polymers purified by polymerization followed by reprecipitation and drying at 25°C for 24 h: The patterns were measured at room temperature without thermal annealing (A, C, E) or after annealing at 50°C for 1h (B, D, F).

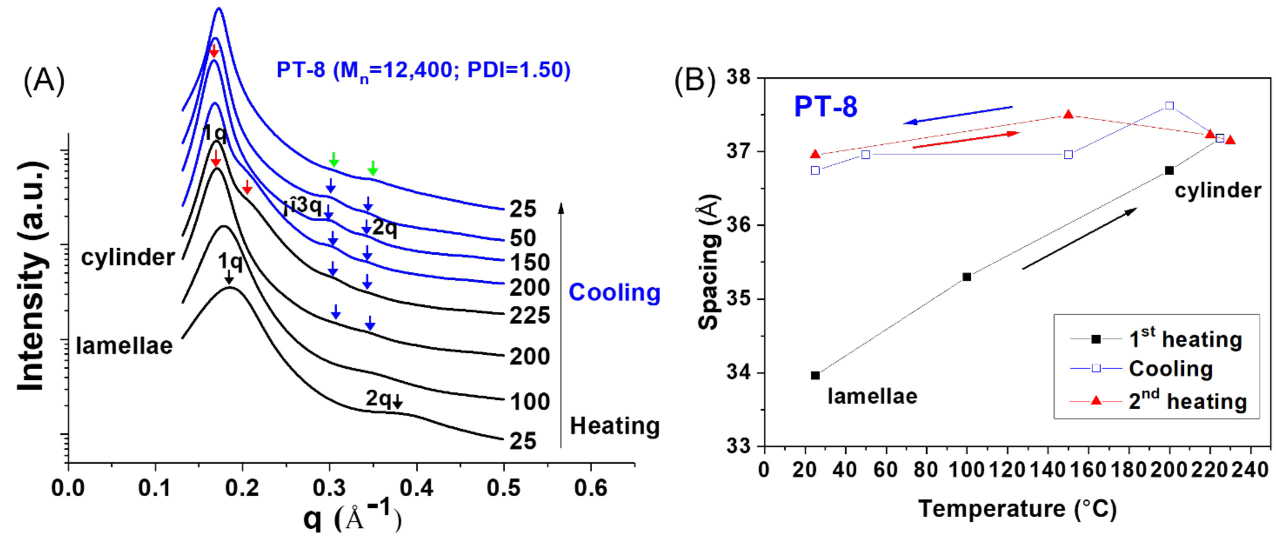

Figure S19. (A) Evolution of the SAXS pattern of PT-8 during successive heating and cooling cycles and (B) evolution of the interdomain spacing of the lamella and hexagonally packed cylinder nanostructures probed in (A). The interdomain spacing (distance, d) is calculated from the equation of d=2 π/q(Å^{-1}) in the SAXS pattern.

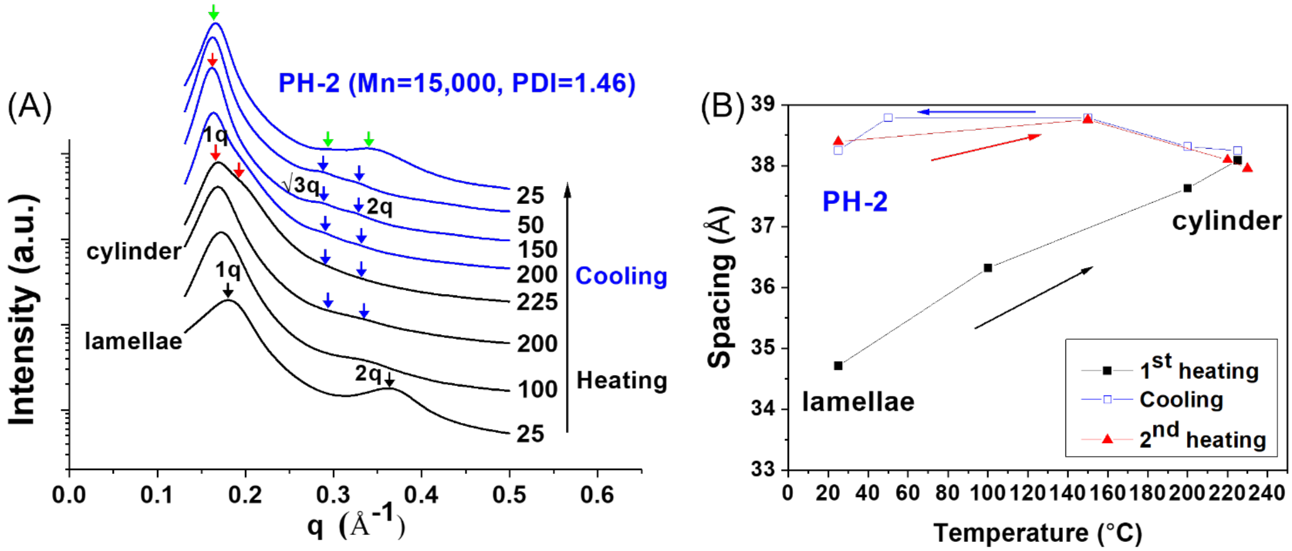

Figure S20. (A) Evolution of the SAXS pattern of PH-2 during successive heating and cooling cycles and (B) evolution of the interdomain spacing of the lamellae and hexagonally packed cylinder nanostructures probed in (A). The interdomain spacing (distance, d) is calculated from the equation of d=2 π/q(Å^{-1}) in the SAXS pattern.

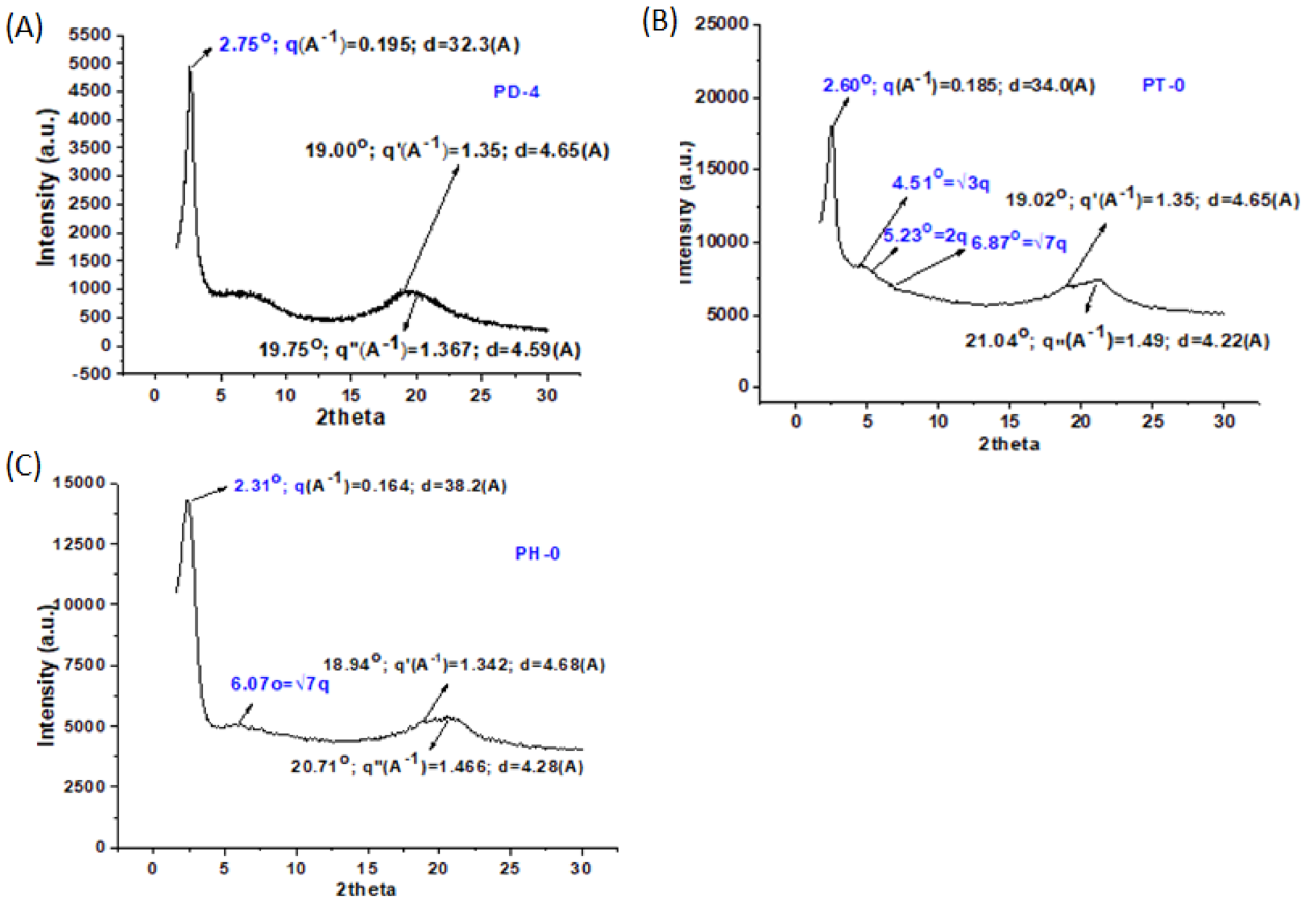

Figure S21. WAXD pattern of mesoporous APAA polymers (A) PD-4, (B) PT-0 and (C) PH-0 purified by polymerization followed by reprecipitation, drying at 25°C for 24 h, and finally annealing at 150°C for 1 h.

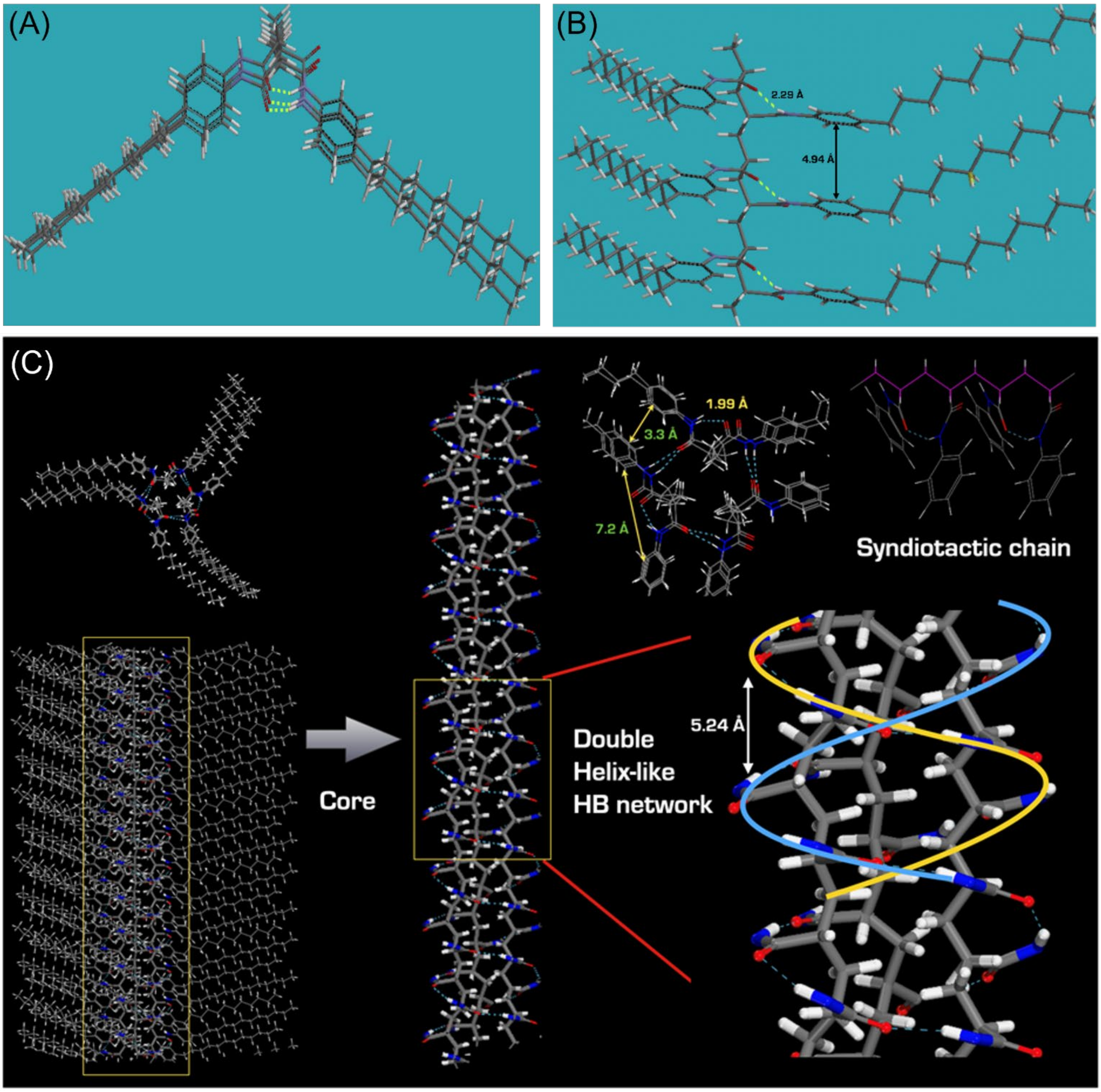

Figure S22. Self-assembly of syndiotactic APAA polymer chains into Y-shaped clusters: Top (A) and side (B) views of a single syndiotactic PDOPAM chain. Hydrogen bonds (HBs) between adjacent units are indicated by green dotted lines. Simulated, energy-minimized PDOPAM assembly structure using SPARTAN-2008. (C) Three syndiotactic PDOPAM chains assemble into a Y-shaped cluster held together by interchain hydrogen bonding, forming a double helix-like interchain HB network in the core of the polymer backbone. This structure is generated by performing molecular dynamics simulations using NAMD and the OPLS force field with periodic boundary conditions.

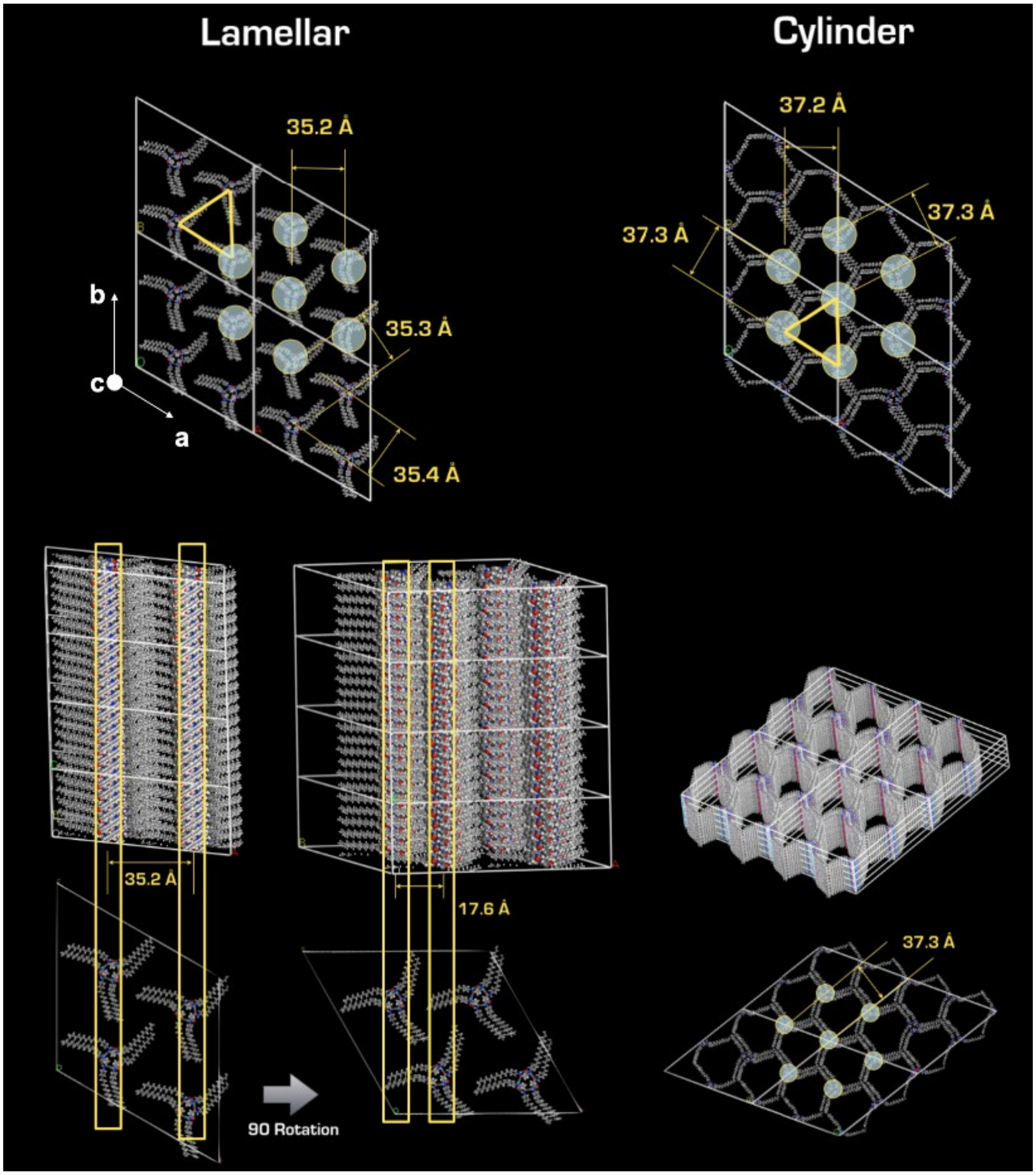

Figure S23. Self-assembled lamellar and hexagonal cylindrical suprastructures of Y-shaped PDOPAM clusters in top and side views.

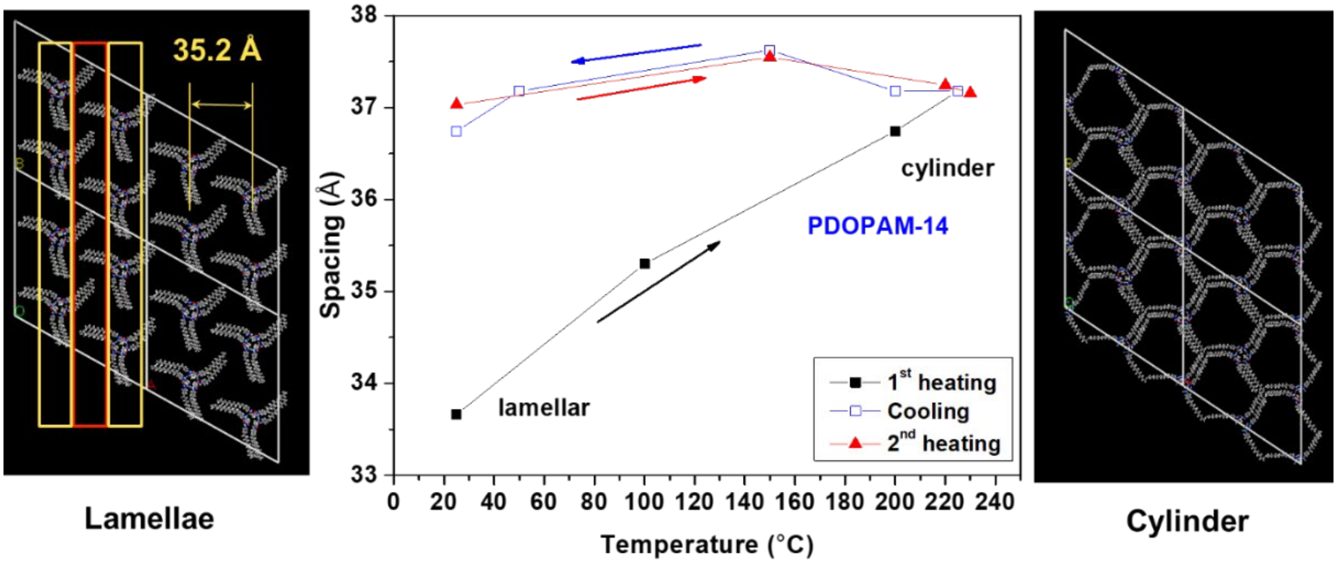

Figure S24. Phase transition behavior of PD from lamellar to hexagonal cylindrical structure by thermal annealing.

(A)

(B)

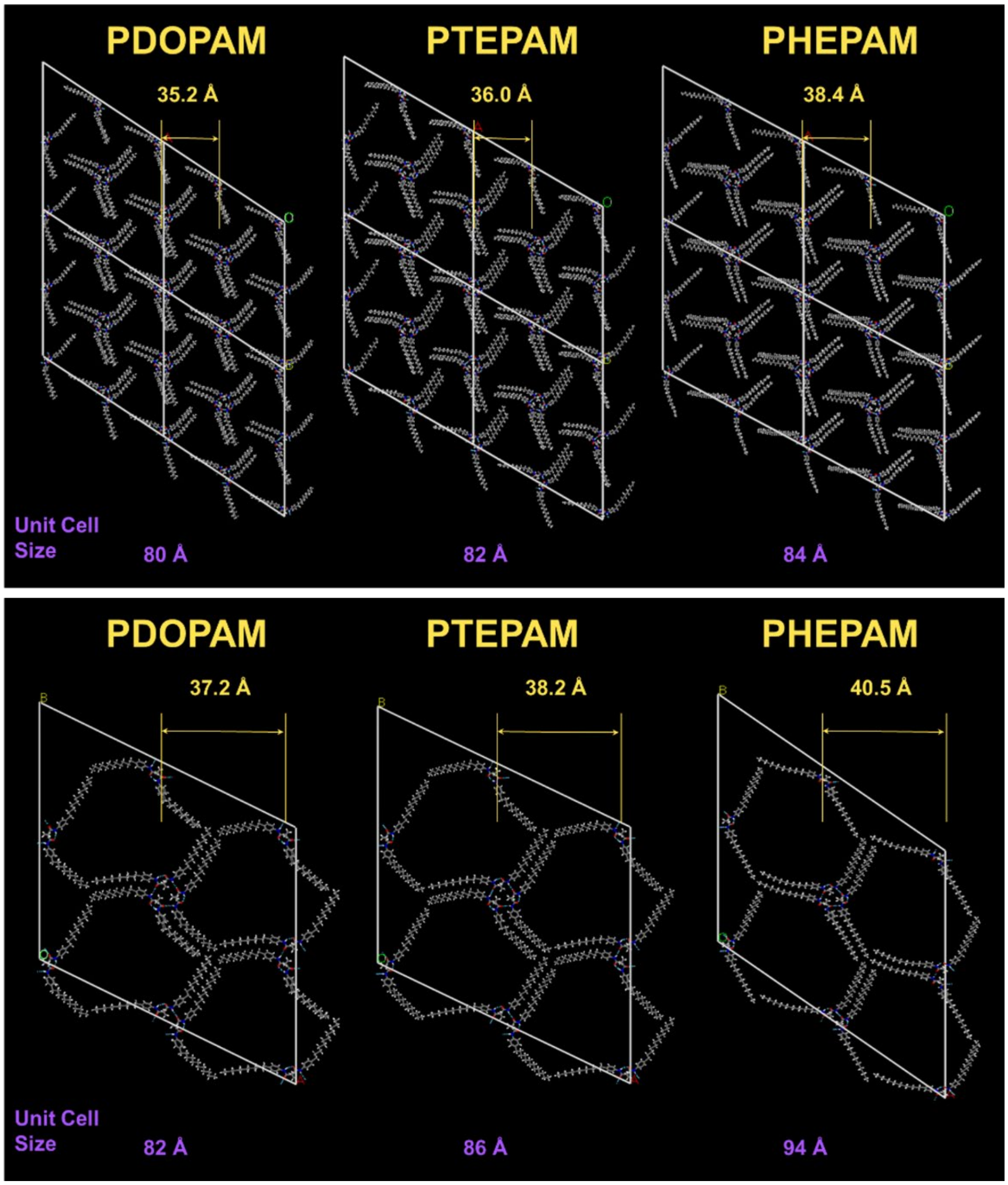

Figure S25. Interdomain spacing of (A) lamellar and (B) hexagonal cylindrical nanostructures of PD, PT, and PH composed of Y-shaped polymer building blocks in a unit cell as calculated by the above molecular mechanics simulations.

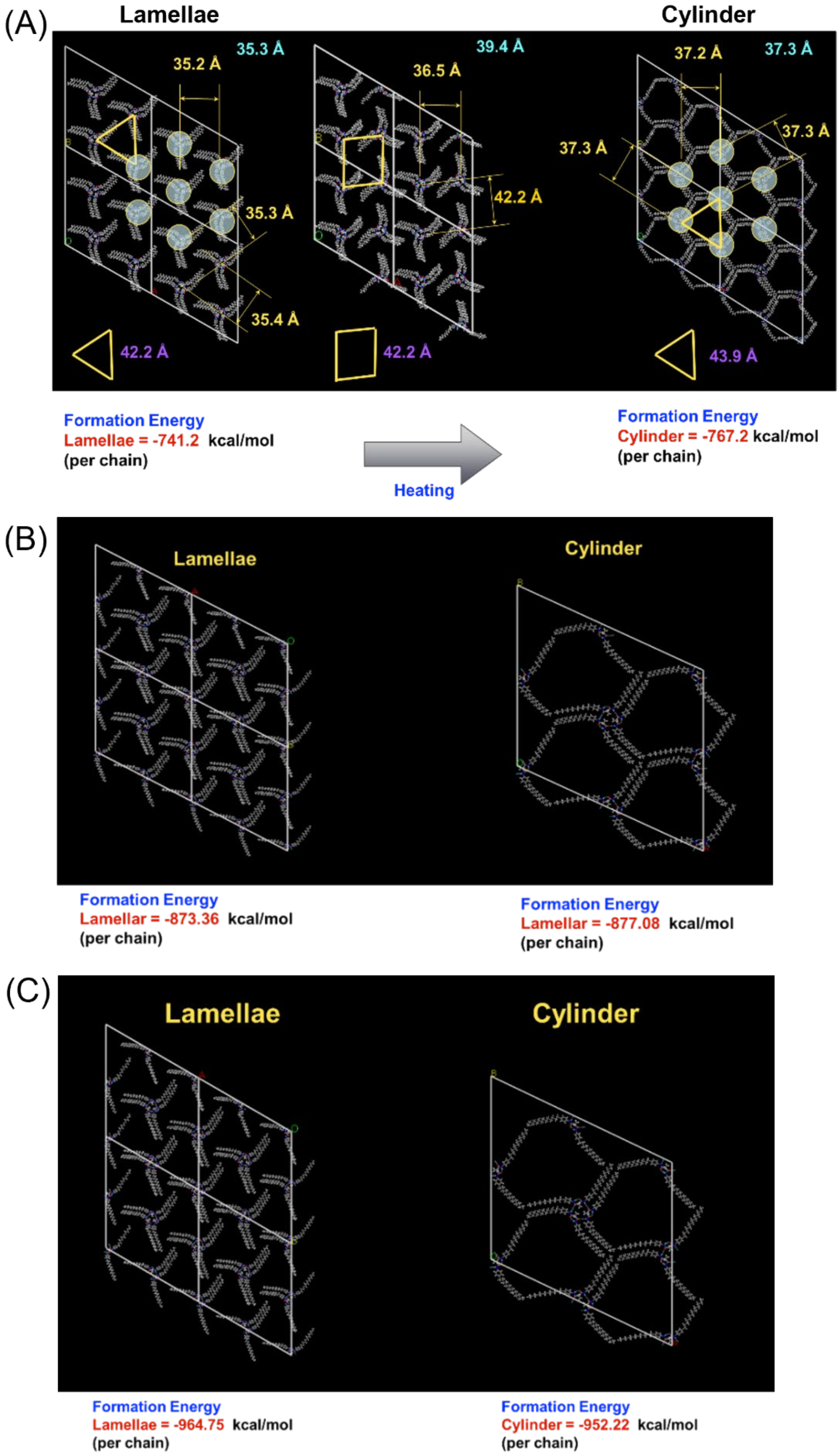

Figure S26. Formation energies of lamellar and hexagonal cylindrical nanostructures consisting of Y-shaped polymer building blocks in a unit cell: (A) PD, (B) PT, and (C) PH.

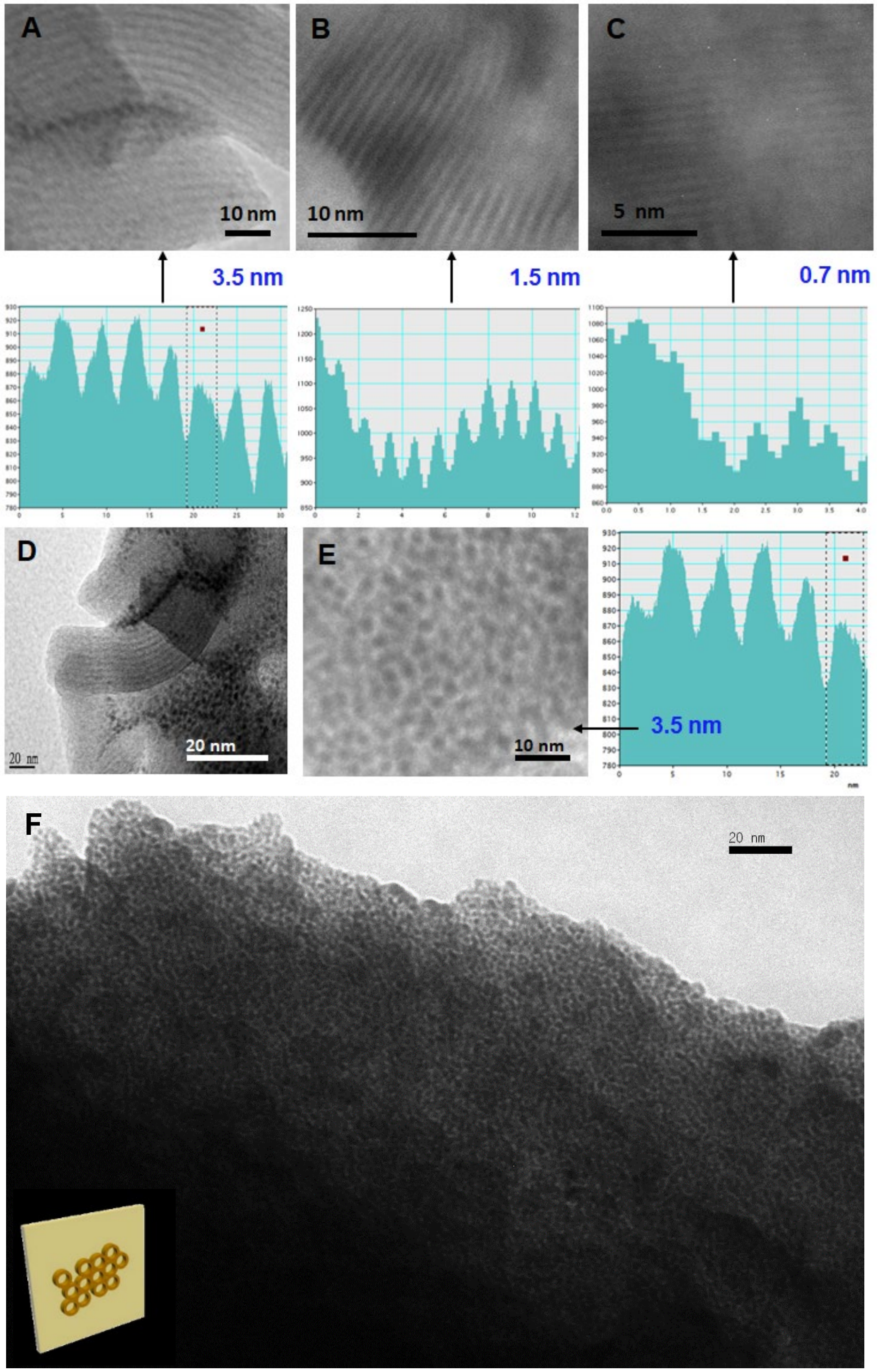

Figure S27. TEM image of the ultrathin film of PD-14: (A-C) side view and (D-F) top view.

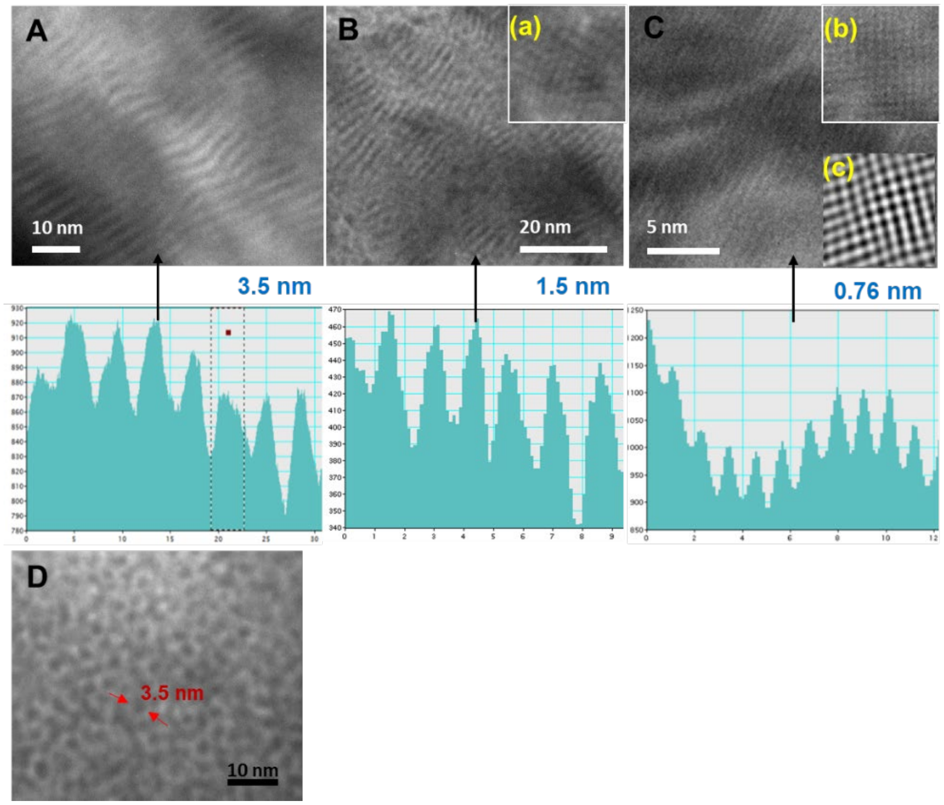

Figure S28. TEM images of the ultrathin film of PT-8: (A, B, C) side view and (D) top view.

(A)

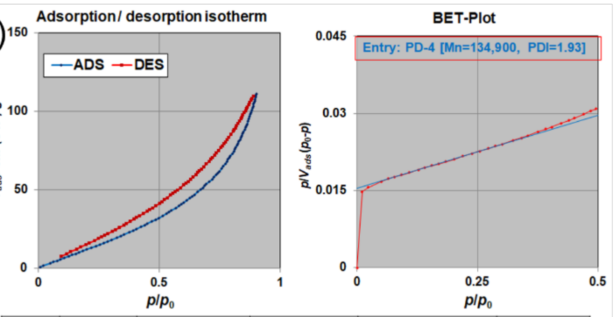

Entry #	Sample weight (g)	V_m (cm^3(STP) g^{-1})	BET surface area (m^2 g^{-1})	Total pore volume (p/p_0 = 0.900) (cm^3 g^{-1})	Mean pore diameter (nm)
PD-4	0.2089	22.863	264.14	0.4427	6.7040

(B)

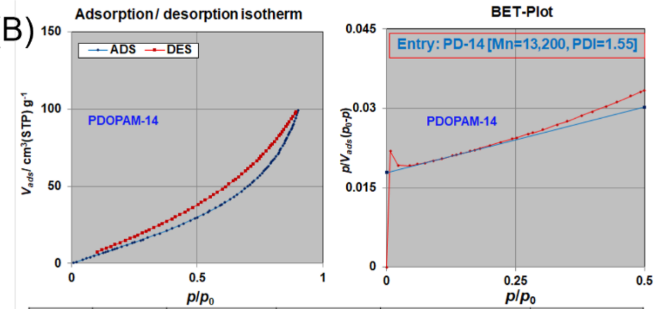

Entry #	Sample weight (g)	V_m (cm^3(STP) g^{-1})	BET surface area (m^2 g^{-1})	Total pore volume (p/p_0 = 0.900) (cm^3 g^{-1})	Mean pore diameter (nm)
PD-14	0.1921	23.41	270.45	0.3958	5.8539

(C)

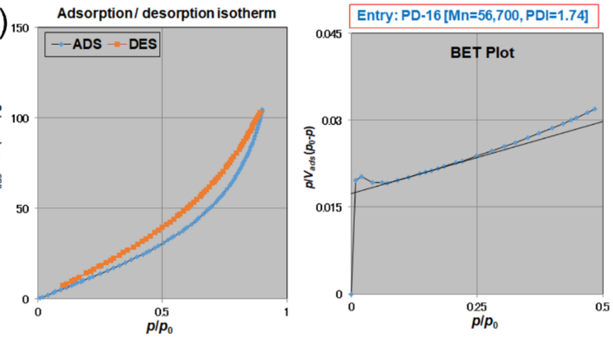

Entry #	Sample weight (g)	V_m (cm^3(STP) g^{-1})	BET surface area (m^2 g^{-1})	Total pore volume (p/p_0 = 0.900) (cm^3 g^{-1})	Mean pore diameter (nm)
PD-16	0.1163	23.716	273.99	0.4177	6.0980

(D)

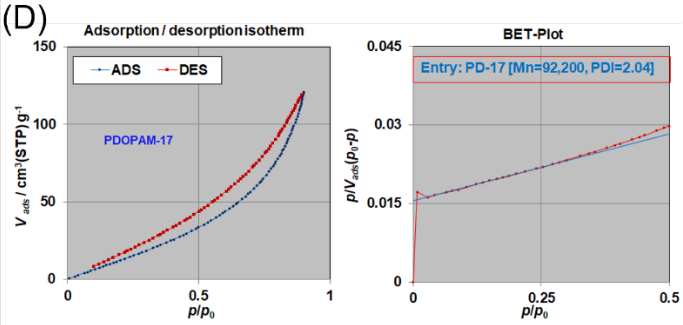

Entry #	Sample weight (g)	V_m (cm^3(STP) g^{-1})	BET surface area (m^2 g^{-1})	Total pore volume (p/p_0 = 0.900) (cm^3 g^{-1})	Mean pore diameter (nm)
PD-17	0.1011	24.344	281.25	0.4826	6.8636

Figure S29. Benzene vapor sorption isotherms of representative PDOPAMs, (A) PD-4, (B) PD-14, (C) PD-16 and (D) PD-17, polymerized with BPO for 0.5 h at 70°C.

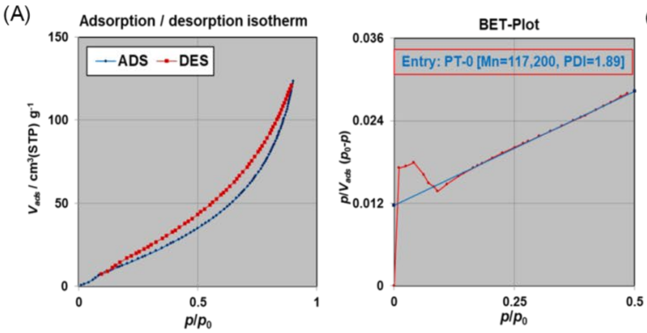

Entry #	Sample weight (g)	V_m (cm³(STP) g⁻¹)	BET surface area (m² g⁻¹)	Total pore volume (p/p_0 = 0.900) (cm³ g⁻¹)	Mean pore diameter (nm)
PT-0	0.1028	22.285	257.46	0.492	7.6439

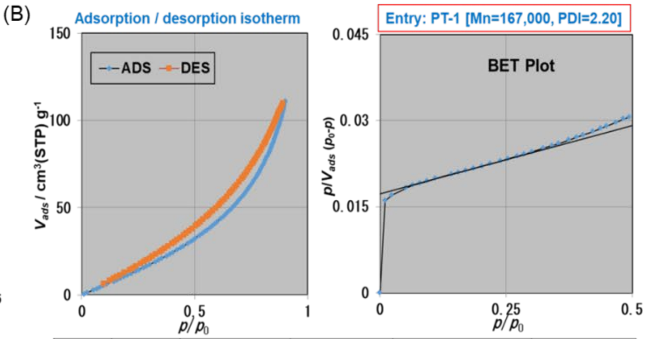

Entry #	Sample weight (g)	V_m (cm³(STP) g⁻¹)	BET surface area (m² g⁻¹)	Total pore volume (p/p_0 = 0.900) (cm³ g⁻¹)	Mean pore diameter (nm)
PT-1	0.2332	24.355	281.37	0.4438	6.3091

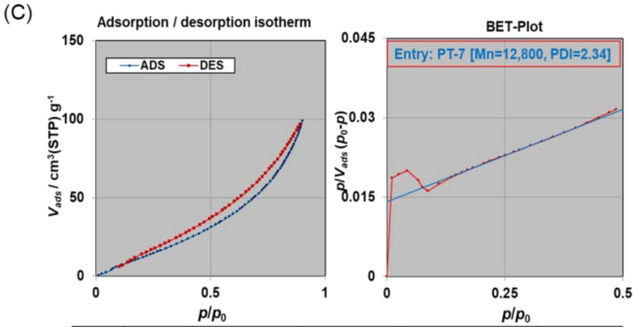

Entry #	Sample weight (g)	V_m (cm³(STP) g⁻¹)	BET surface area (m² g⁻¹)	Total pore volume (p/p_0 = 0.900) (cm³ g⁻¹)	Mean pore diameter (nm)
PT-7	0.1093	20.396	235.63	0.3948	6.7020

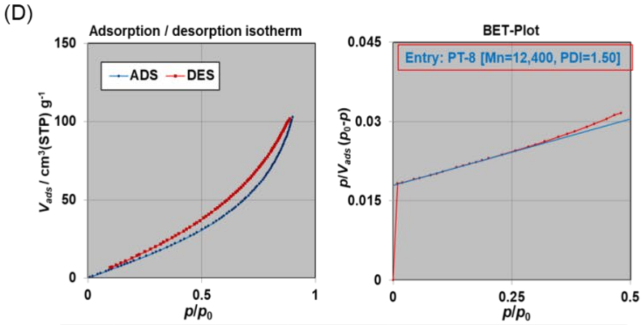

Entry #	Sample weight (g)	V_m (cm³(STP) g⁻¹)	BET surface area (m² g⁻¹)	Total pore volume (p/p_0 = 0.900) (cm³ g⁻¹)	Mean pore diameter (nm)
PT-8	0.3225	23.217	268.23	0.4106	6.1231

Figure S30. Benzene vapor sorption isotherms of representative PTEPAMs, (A) PT-0, (B) PT-1 (1h), (C) PT-7 (THF, 80°C, 48h) and (D) PT-8, polymerized with BPO for 0.5 h at 70°C.

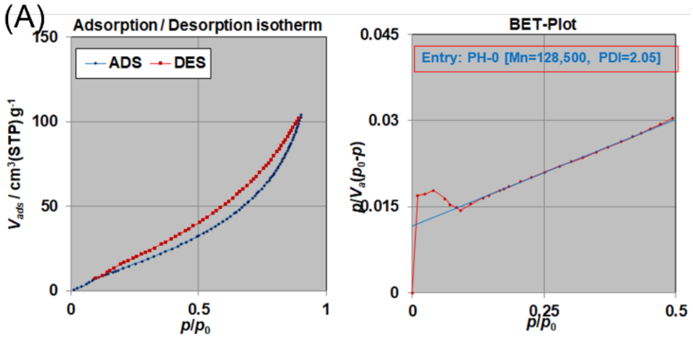

Entry #	Sample weight (g)	V_m (cm³(STP) g⁻¹)	BET surface area (m² g⁻¹)	Total pore volume (p/p_0 = 0.900) (cm³ g⁻¹)	Mean pore diameter (nm)
PH-0	0.2246	20.6370	238.84	0.4224	7.0742

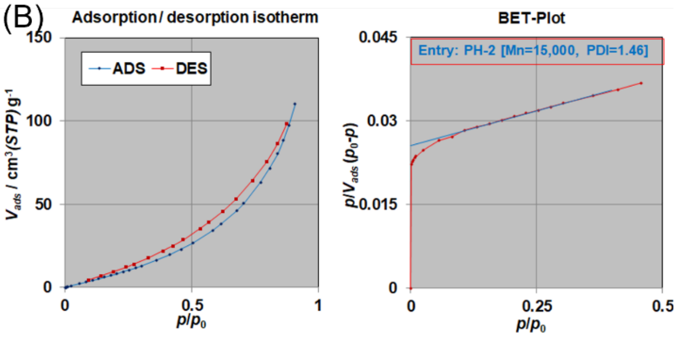

Entry #	Sample weight (g)	V_m (cm³(STP) g⁻¹)	BET surface area (m² g⁻¹)	Total pore volume (p/p_0 = 0.900) (cm³ g⁻¹)	Mean pore diameter (nm)
PH-2	0.2352	20.2012	226.28	0.4401	7.7797

Figure S31. Benzene vapor sorption isotherms of representative PHEPAMs, (A) PH-0 and (B) PH-2, polymerized with BPO for 0.5 h at 70°C.

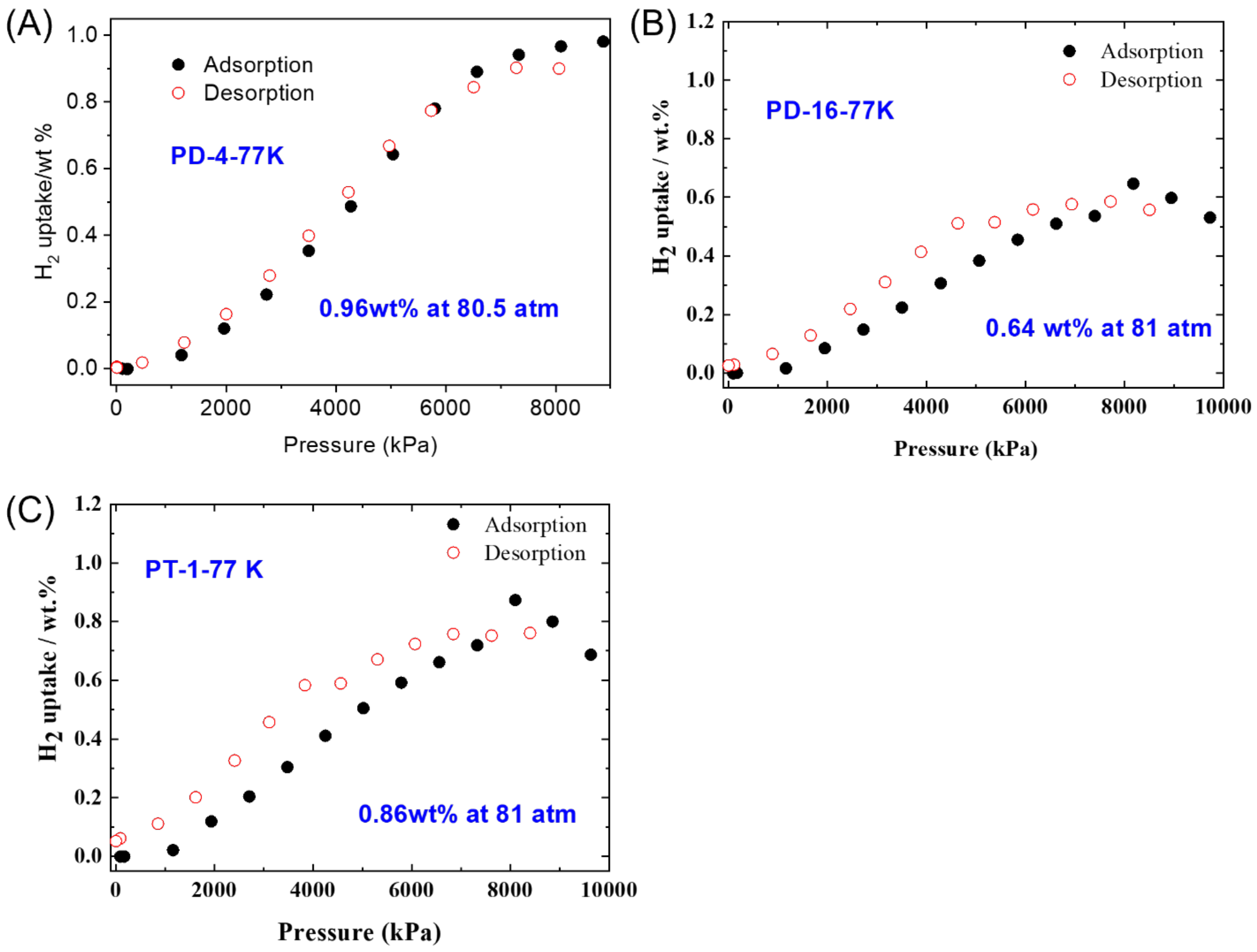

Figure S32. High pressure hydrogen sorption isotherms of (A) PD-4, (B) PD-16, and (C) PT-1 powders at 77 K.

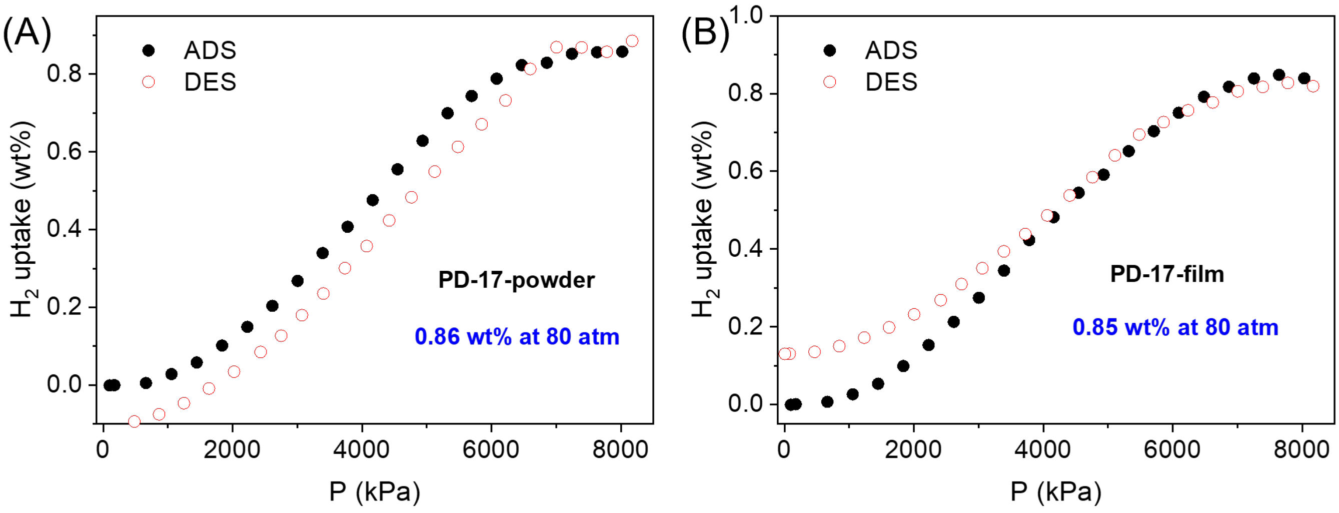

Figure S33. High pressure hydrogen sorption isotherms of (A) PD-17 powder and (B) its film at 77 K.

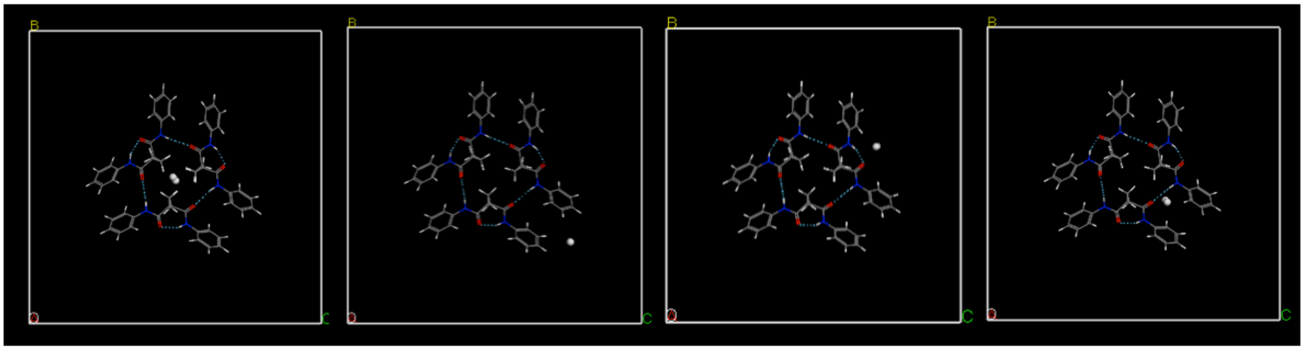

ΔE	4.17 kcal/mol	0.20 kcal/mol	0.12 kcal/mol	0 kcal/mol
E_{bind}	3.88 kcal/mol	-0.10 kcal/mol	-0.17 kcal/mol	-0.29 kcal/mol

Figure S34. Hydrogen gas adsorption capacity depending on different binding sites of PD.

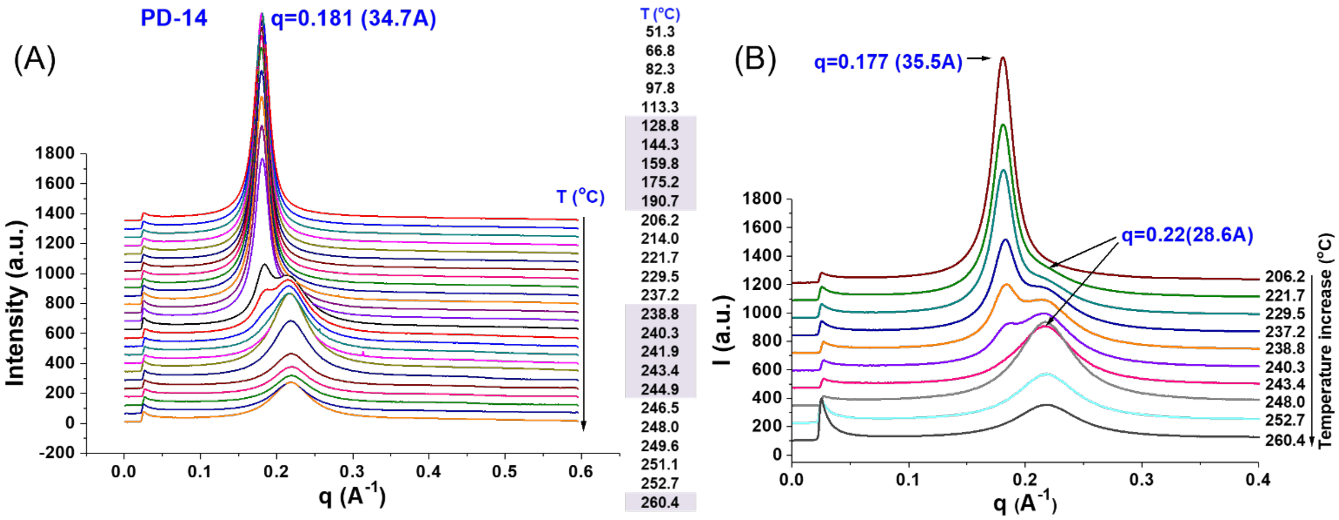

Figure S35. Evolution of the SAXS pattern of PD-14 during continuous heating in the range of (A) 51~261°C and (B) 206~261°C without cooling.

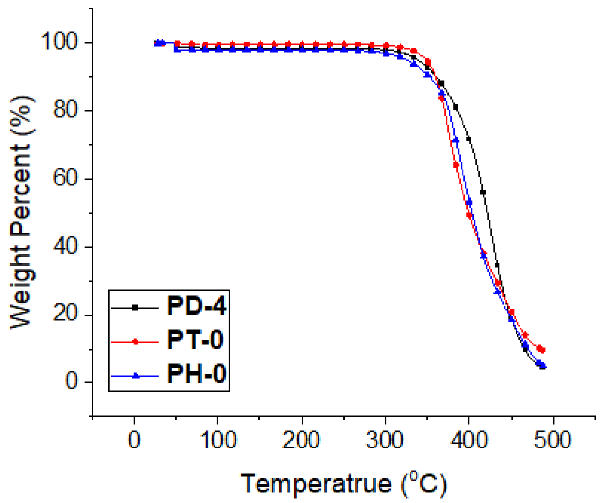

Figure S36. TGA thermogram of mesoporous APAA polymers versus temperature: Their weight loss temperatures at 3%, 5% and 10% are listed in Table S4.

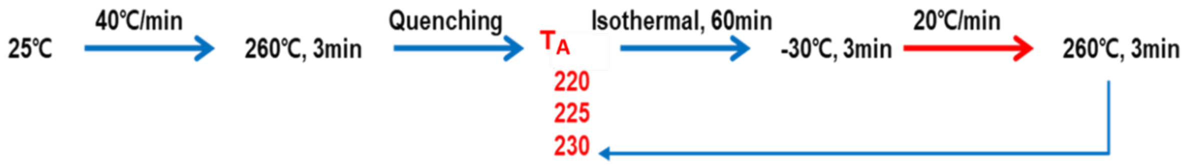

Figure S37. Diagram of the isothermal annealing process of APAA polymers prior to DSC measurements (T_A: annealing temperature).

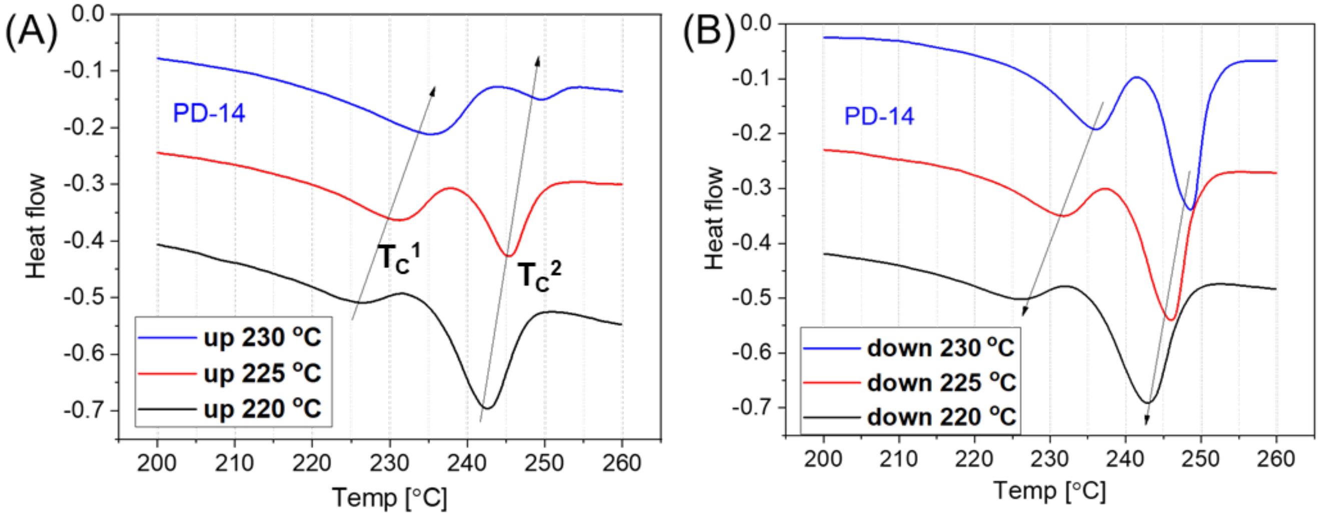

Figure S38. DSC thermograms obtained by heating the polymer PD-14 at different annealing temperatures (T_A) in (A) ascending and (B) descending order: A detailed thermal history is shown in **Figure S37**. Changes in enthalpy and critical phase transition temperature (T_C) with T_A are listed in **Table S5**.

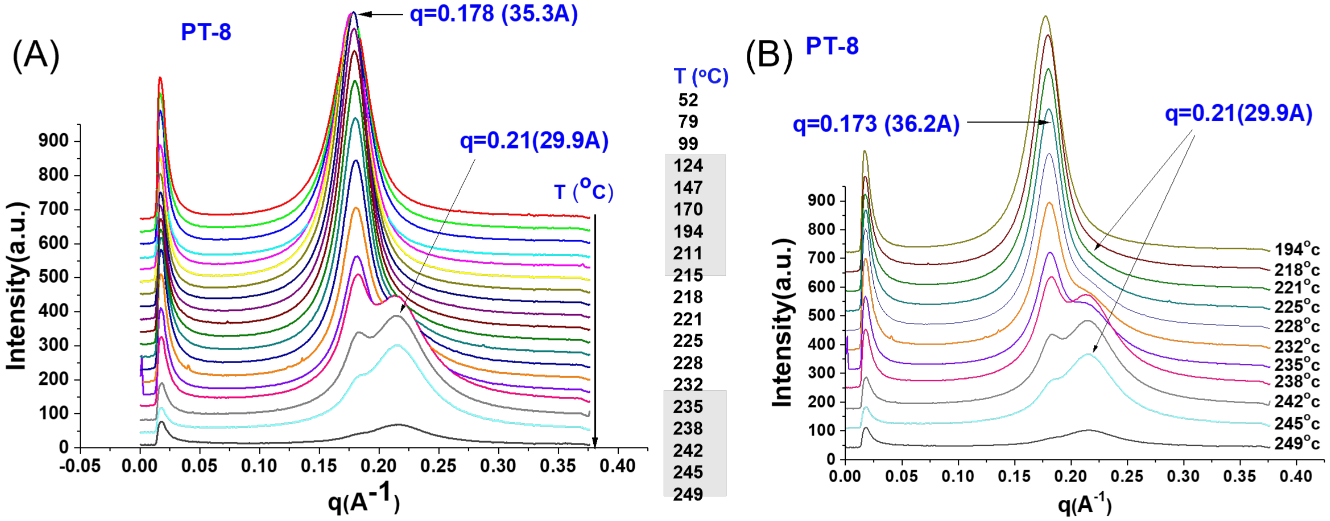

Figure S39. Evolution of the SAXS pattern of PT-8 during continuous heating in the range of (A) 52-249°C and (B) 194-249°C without cooling.

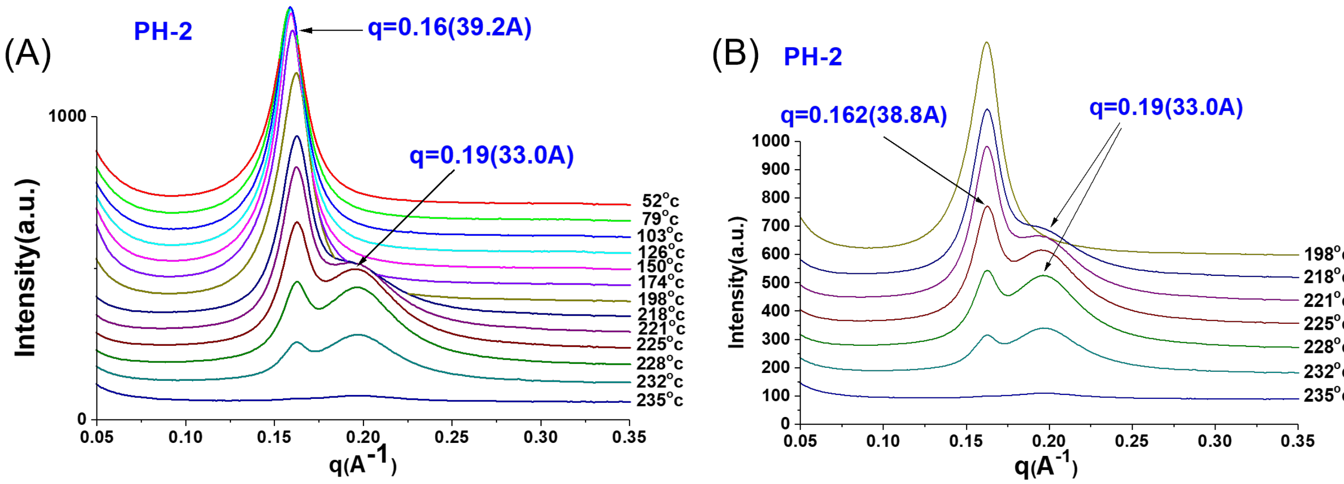

Figure S40. Evolution of the SAXS pattern of PH-2 during continuous heating in the range of (A) 52-235°C and (B) 198-235°C without cooling.

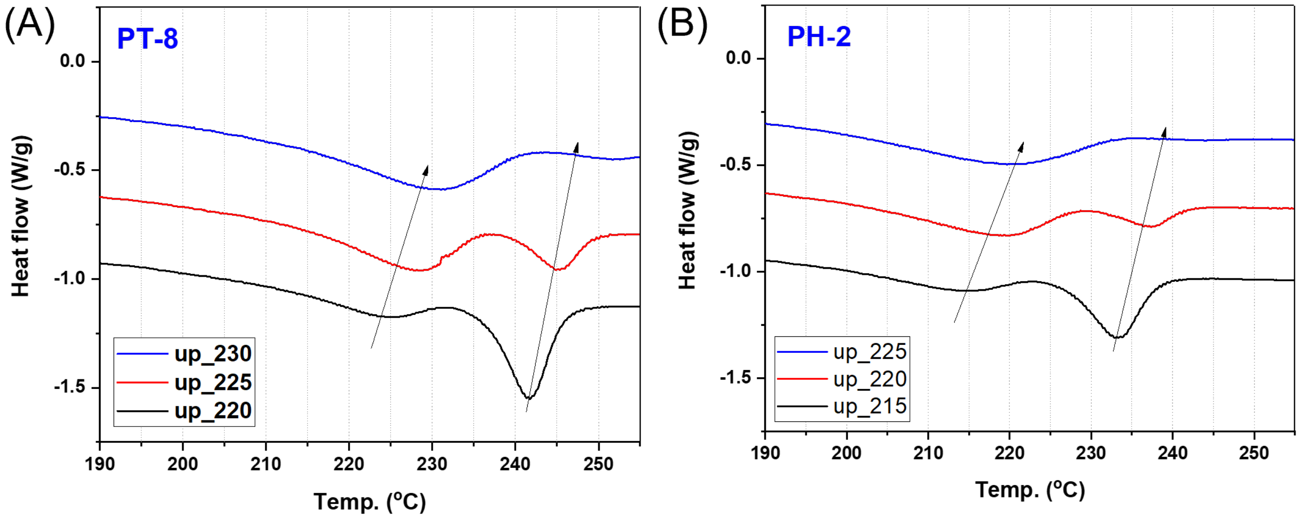

Figure S41. DSC thermograms obtained by heating the polymers (A) PT-8 and (B) PH-2 at different annealing temperatures (T_A) in ascending order. A detailed thermal history is shown in Figure S37.

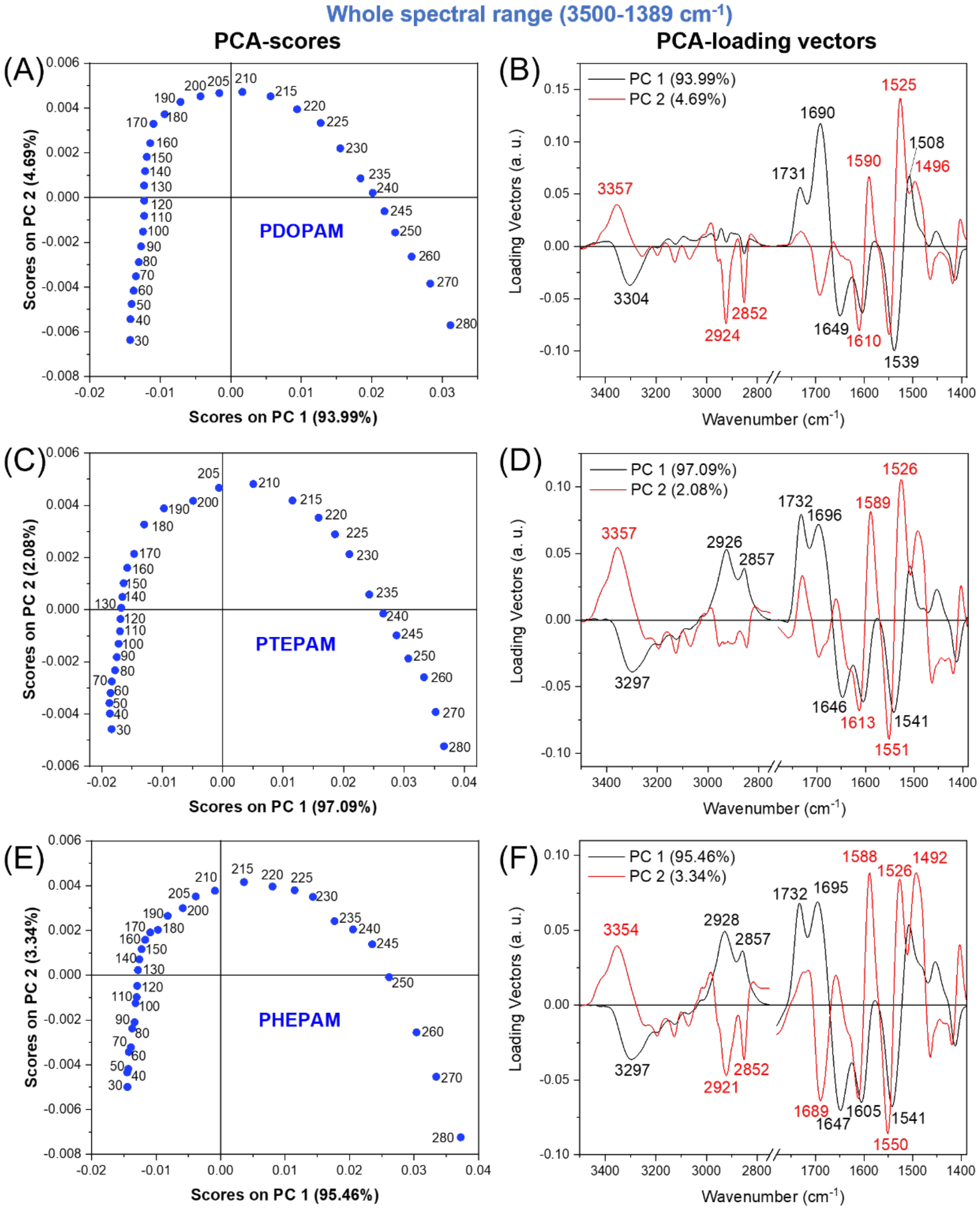

Figure S42. Principal component analysis of one-dimensional FT-IRRAS spectra of APAA polymers. (A, C, E) Score plots of the spectra for the first two principal components of PDOPAM (PD-14), PTEPAM (PT-8) and PHEPAM (PH-2). (B, D, F) Plots of the loading vectors of the spectra for PC1 (black line) and PC2 (red line) in the region of the N-H and C-H stretching vibrations (3500-2750 cm^{-1}) and the amide I and II vibrations (1750-1400 cm^{-1}).

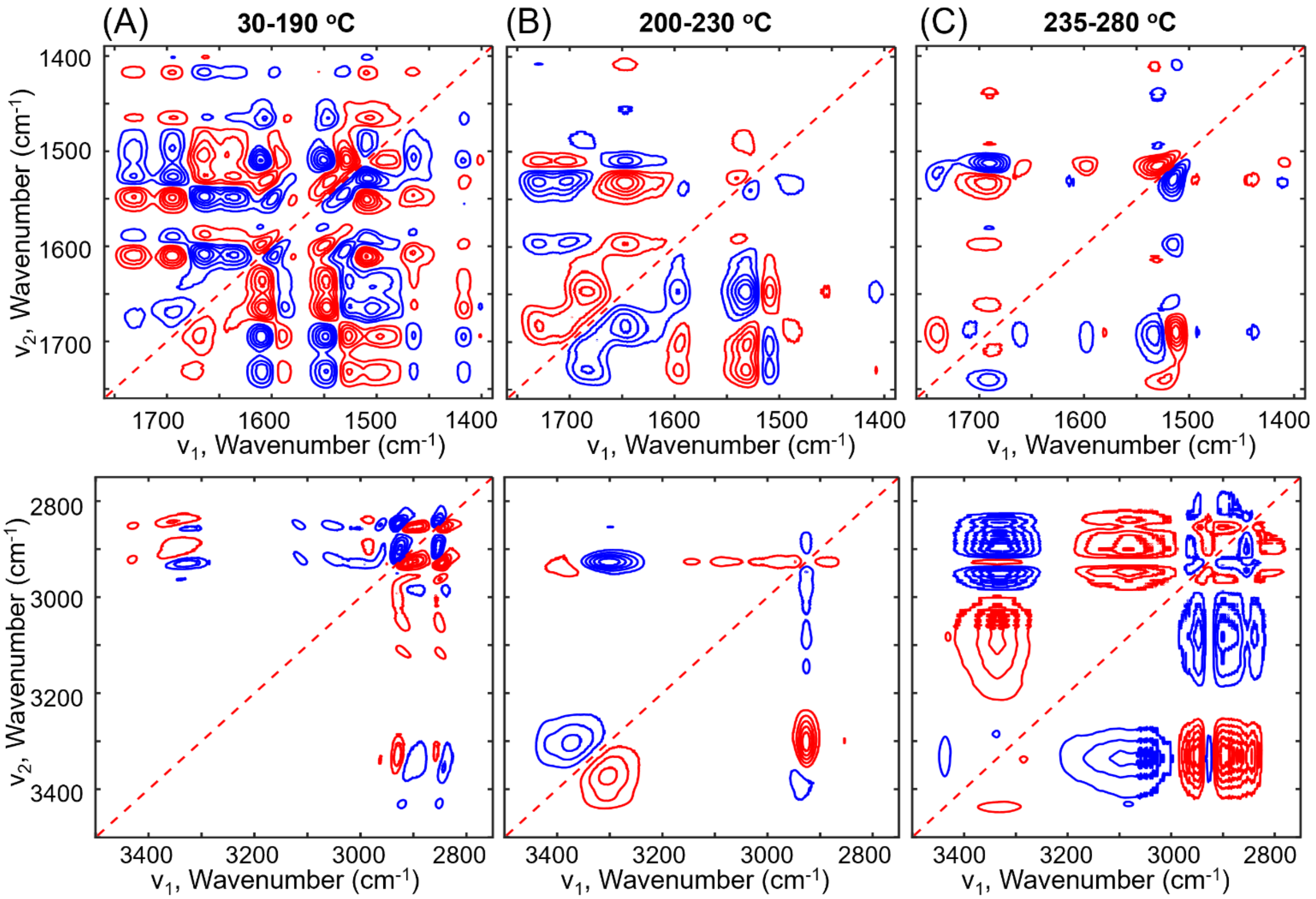

Figure S43. Asynchronous 2D correlation spectra of PD-14 in the region of amide I and II vibrations (1750-1400 cm^{-1}) and N-H and C-H stretching vibrations (3500-2750 cm^{-1}) obtained in three different temperature ranges (30-190, 200-230, and 235-280°C). The red and blue lines represent positive and negative cross peaks, respectively.

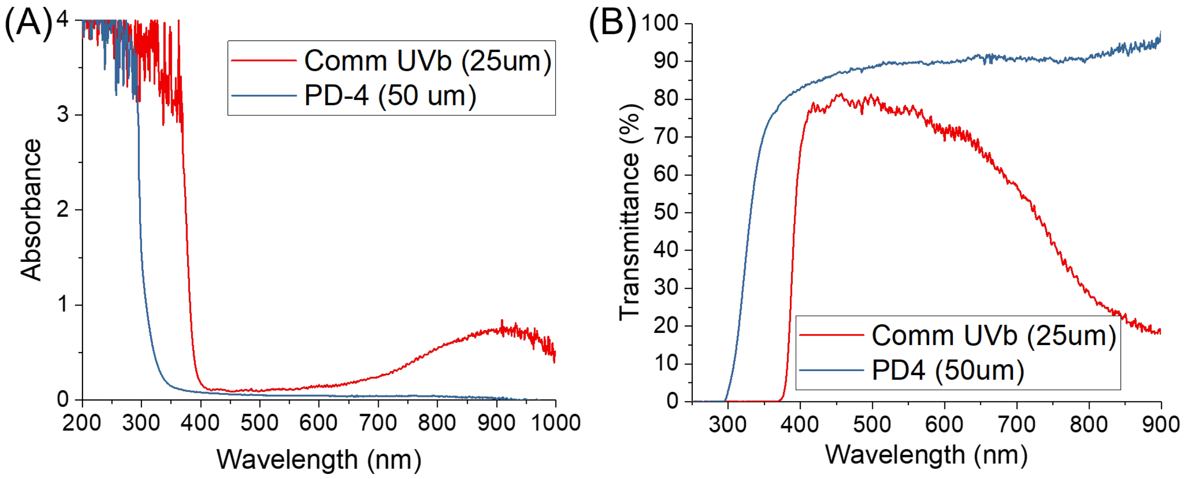

Figure S44. Comparison of commercial PET-based UV blocking film (Decorative Films, IR8360, 25 um) and PD-4 (50um) for their (A) absorbance and (B) transmittance.

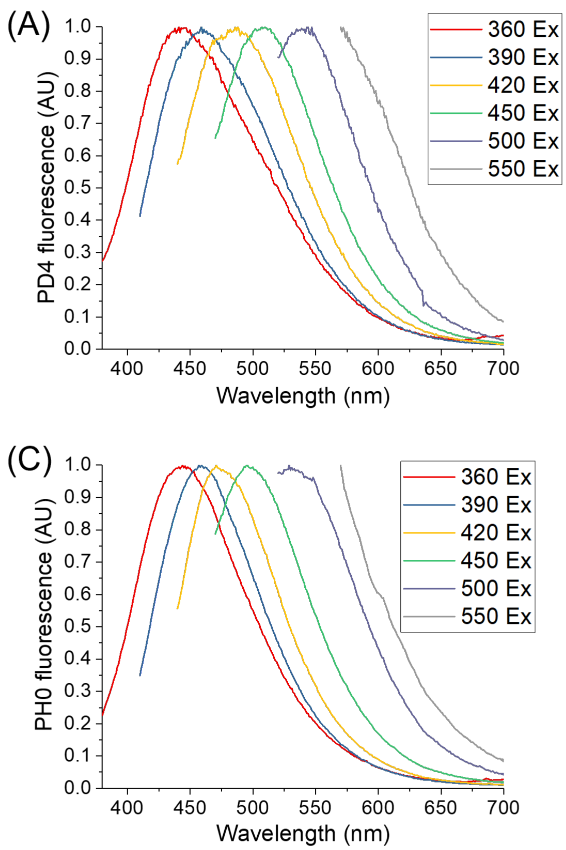

Figure S45. Normalized fluorescence with different excitation wavelengths: (A) PD-4, (B) PT-0 and (C) PH-0. Excitation at higher wavelengths results in red-shifted emission.

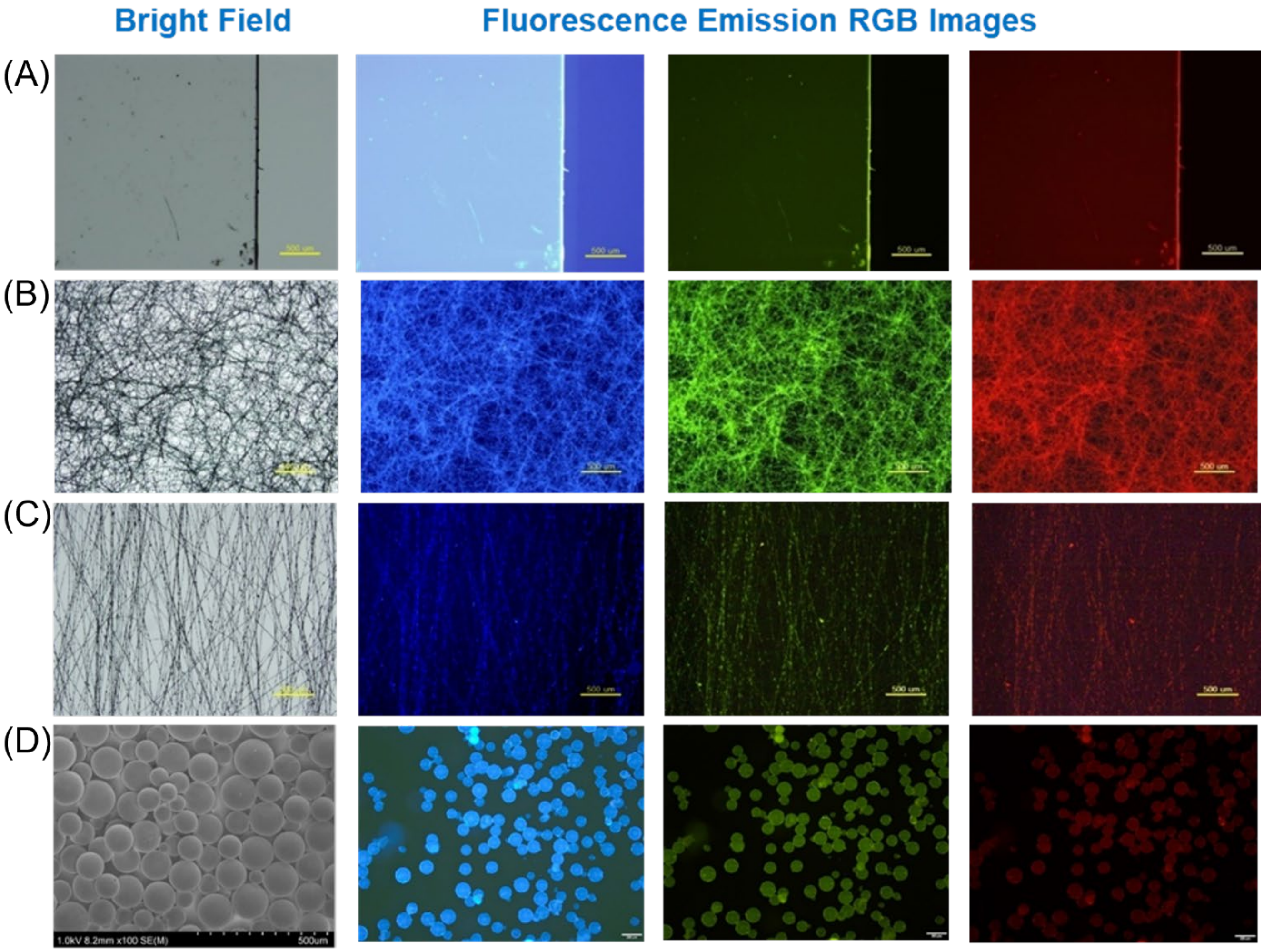

Figure S46. Bright field and fluorescence emission RGB (red/green/blue) images of PD-4 film (100 μm thick, A), its nonaligned/aligned eletrospinning fiber (d=4-5 μm, B/C), and crosslinked PD particles (average d=120 μm, D). Bright field photographs were taken using optical microscope (A/B/C) and SEM (D). The RGB images of the film, fiber, and particle were observed using a fluorescence microscope (OLYMPUS BX51) in red (U-MWG2 filter, Ex: 510-550 nm, Em: >590 nm), green (U-MWB2 filter, Ex: 460-490 nm, Em: >520 nm), and blue (U-MWU2 filter, Ex: 330-385 nm, Em: >420 nm), respectively, where the exposure time and scale bar are 300 ms and 500 μm for the film and fiber, but 400 ms and 200 μm for the particle: Diameter (d), Excitation (Ex) and Emission (Em).

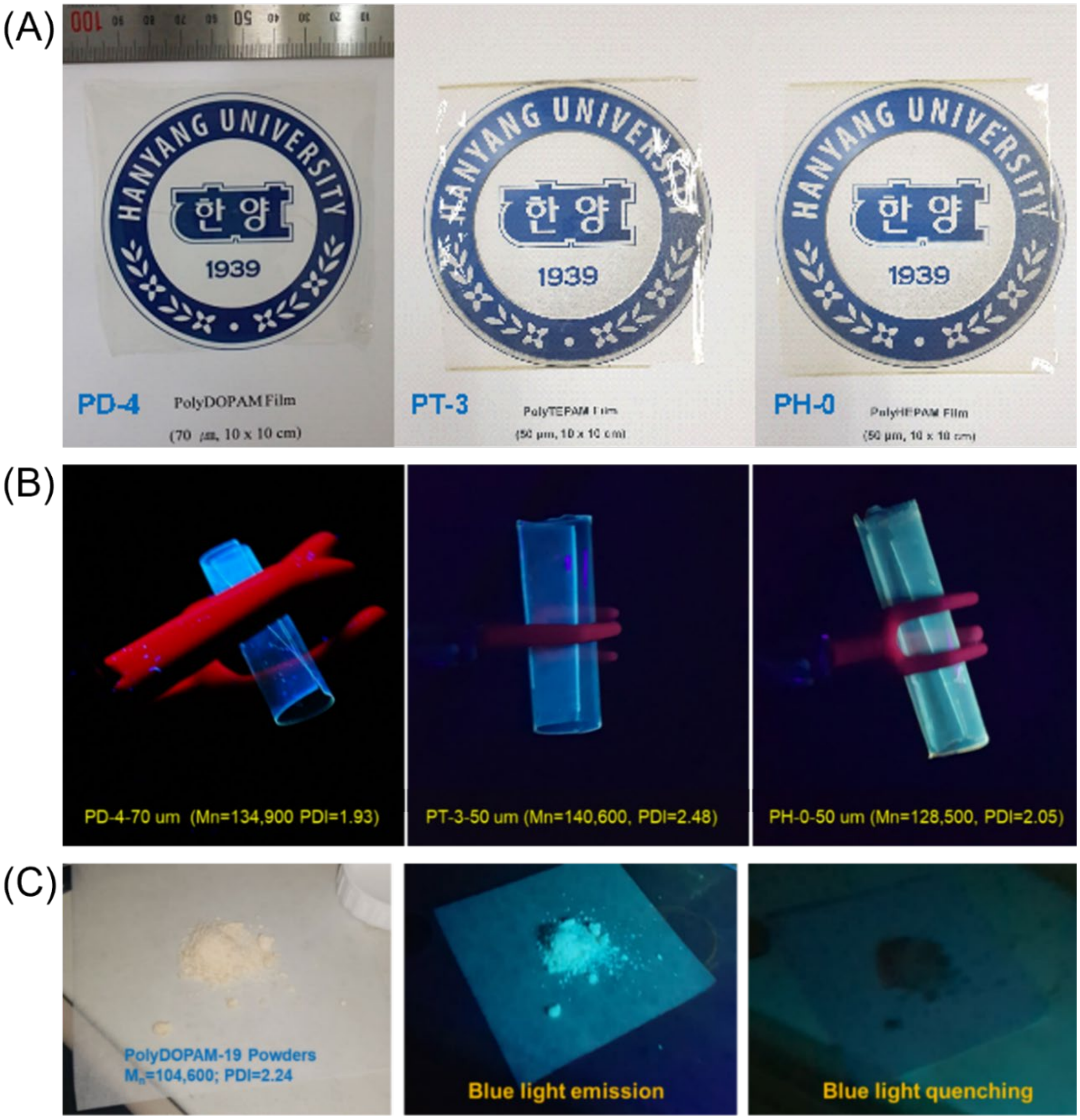

Figure S47. Optical properties of mesoporous APAA polymers: (A) films with high transparency above 80% in the visible range (400~800 nm), (B) blue light emission of thick films when excited at 365nm, (C) PD-19 shows blue light emission or quenching when excited at 365 or 254 nm, respectively, using a portable UV lamp.

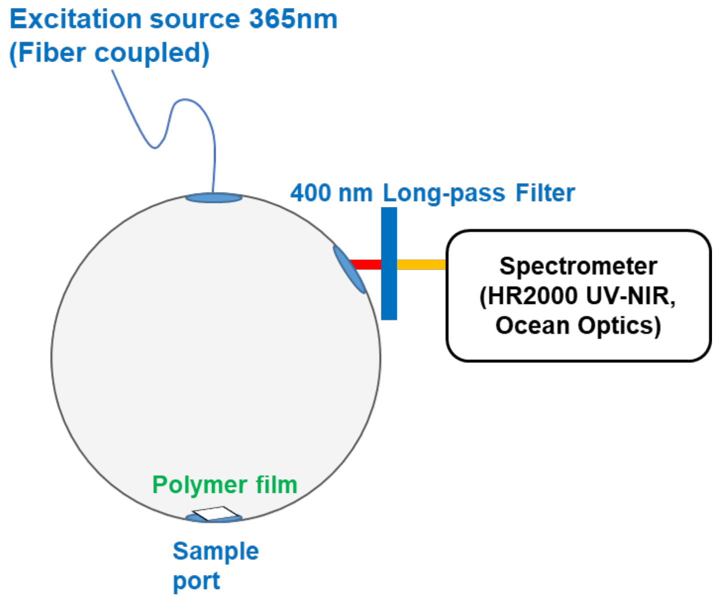

Figure S48. Optical setup for measuring the photoluminescence quantum yield of APAA polymer films.

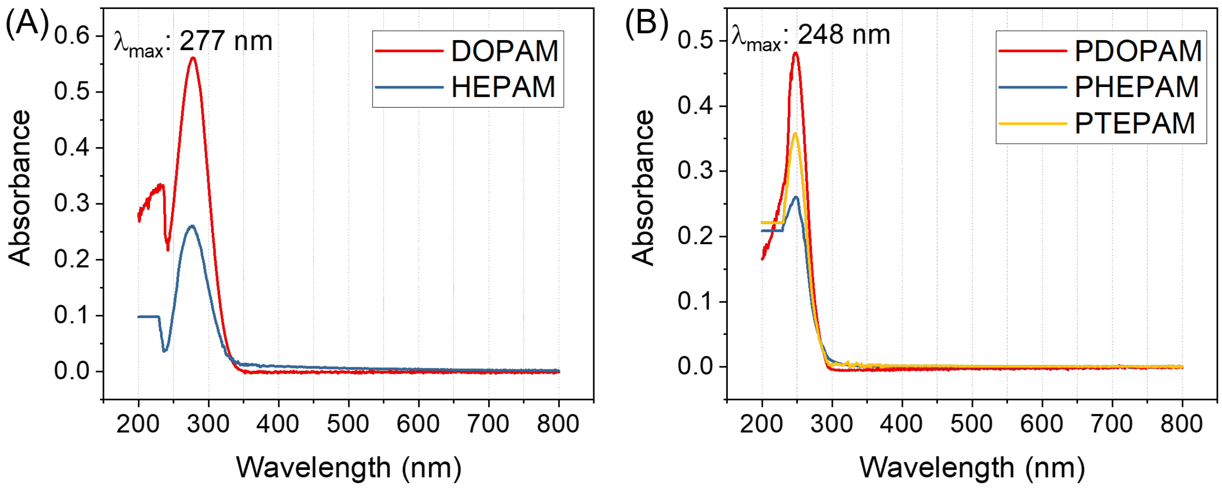

Figure S49. UV-Vis spectra of 0.001 wt% chloroform solutions of (A) APAA monomers and (B) their polymers.

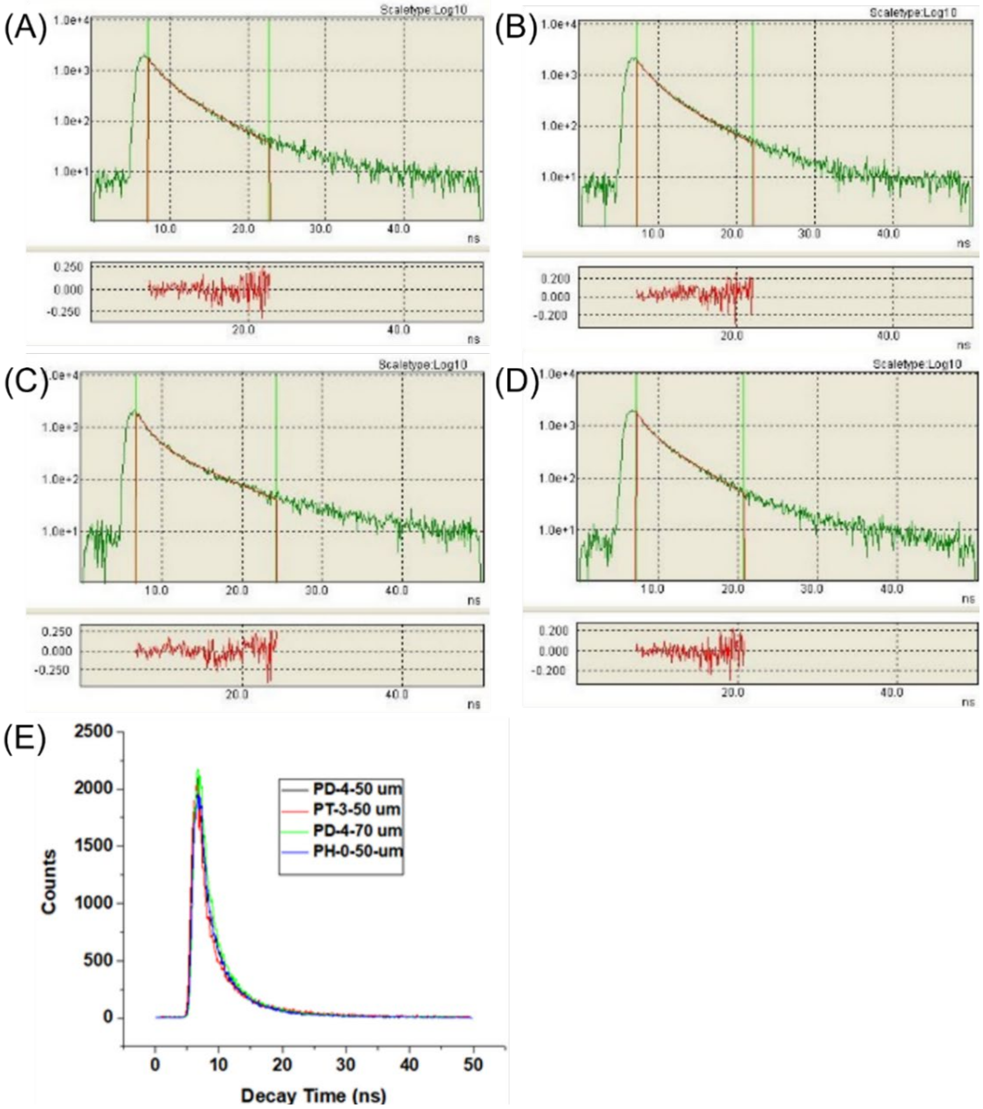

Figure S50. Time-resolved fluorescence decay curve of APAA polymer films when excited at 374 nm: (A) PD-4 (50 μm), (B) PD-4 (70 μm), (C) PT-3 (50 μm), (D) PH-0 (50 μm), and (E) combined plots. The resulting lifetimes, fractional intensities and reduced chi-squares are given in **Table S7**.

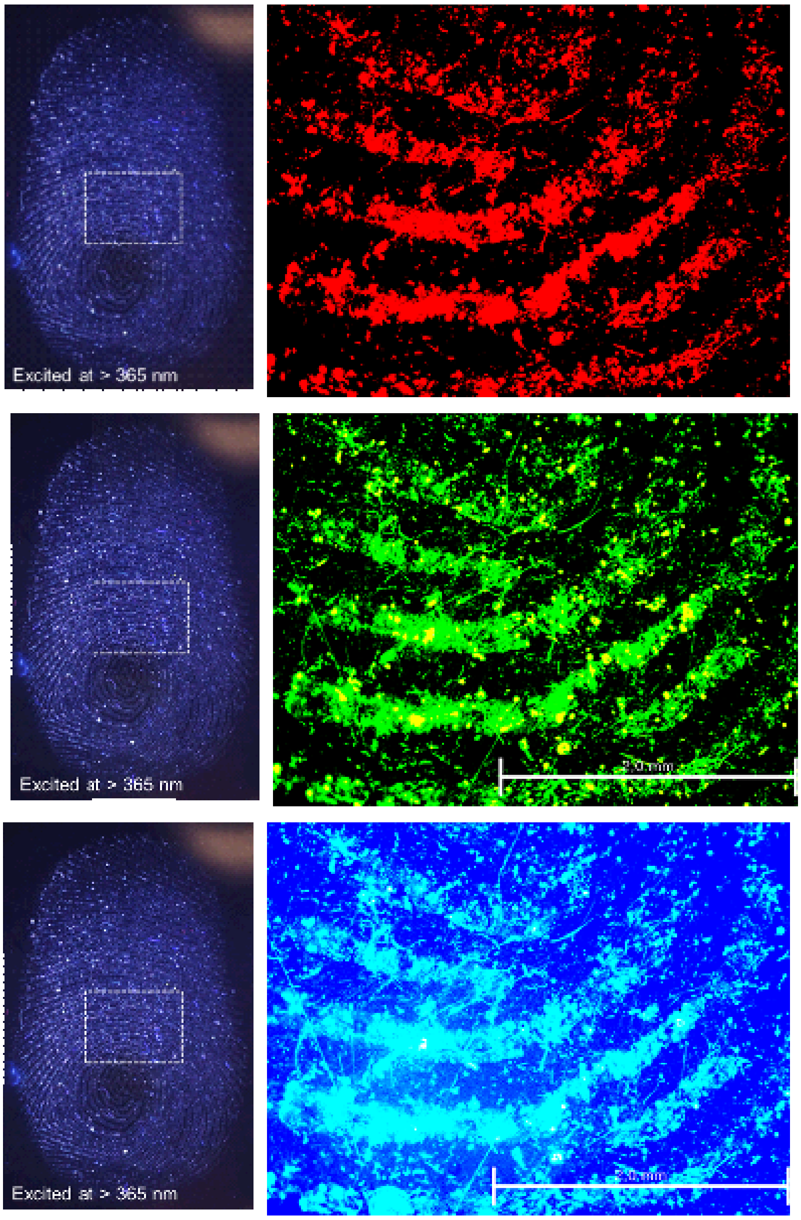

Figure 51. PL images of thumbprint on 60 μm PD-4 film (**left**) emitted by a thumbprint when excited by a 365 nm portable UV lamp, and enlarged RGB images (**right**).

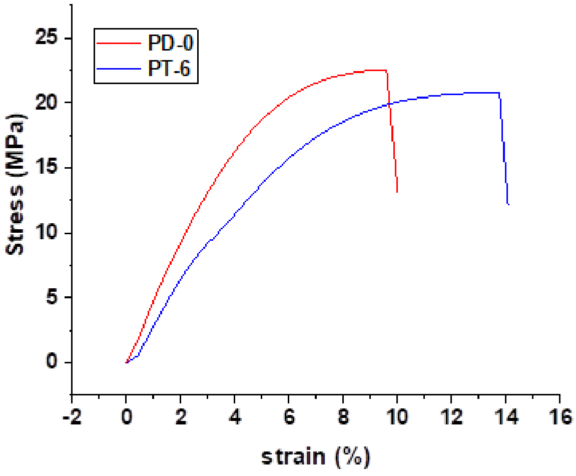

Figure S52. Stress-Strain curves of PD-0 and PT-6 measured with an Instron^R tester: PD-0, M_n=116,900, PDI=2.84; PT-6, M_n=80,900, PDI=4.4. Their tensile properties are summarized in **table S8.**

(A)

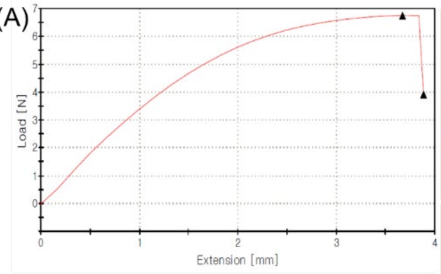

	Maximum Load [N]	Tensile stress at Maximum Load [MPa]	Tensile strain (Extension) at Maximum Load [%]	Load at Break (Standard) [N]	Tensile stress at Break (Standard) [MPa]	Tensile strain (Extension) at Break (Standard) [%]
1	6.75	22.50	9.17	3.92	13.08	9.70
Coefficient of variation	-----	-----	-----	-----	-----	-----
Maximum	6.75	22.50	9.17	3.92	13.08	9.70
Mean	6.75	22.50	9.17	3.92	13.08	9.70
Median	6.75	22.50	9.17	3.92	13.08	9.70
Minimum	6.75	22.50	9.17	3.92	13.08	9.70
Range	0.00	0.00	0.00	0.00	0.00	0.00
Standard deviation	-----	-----	-----	-----	-----	-----

	Modulus (Automatic) [MPa]
1	473.92223
Coefficient of variation	-----
Maximum	473.92223
Mean	473.92223

(B)

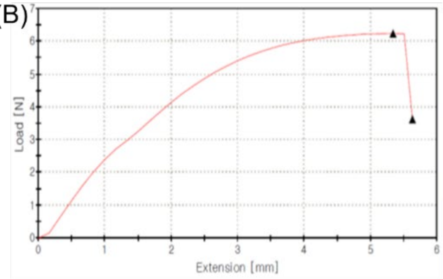

	Maximum Load [N]	Tensile stress at Maximum Load [MPa]	Tensile strain (Extension) at Maximum Load [%]	Load at Break (Standard) [N]	Tensile stress at Break (Standard) [MPa]	Tensile strain (Extension) at Break (Standard) [%]
1	6.24	20.79	13.33	3.63	12.10	14.06
Coefficient of variation	-----	-----	-----	-----	-----	-----
Maximum	6.24	20.79	13.33	3.63	12.10	14.06
Mean	6.24	20.79	13.33	3.63	12.10	14.06
Median	6.24	20.79	13.33	3.63	12.10	14.06
Minimum	6.24	20.79	13.33	3.63	12.10	14.06
Range	0.00	0.00	0.00	0.00	0.00	0.00
Standard deviation	-----	-----	-----	-----	-----	-----

	Modulus (Automatic) [MPa]
1	359.36450
Coefficient of variation	-----
Maximum	359.36450
Mean	359.36450

Figure S53. Raw data for tensile properties of (A) PD-0 and (B) PT-6 measured on an Instron^R tester.

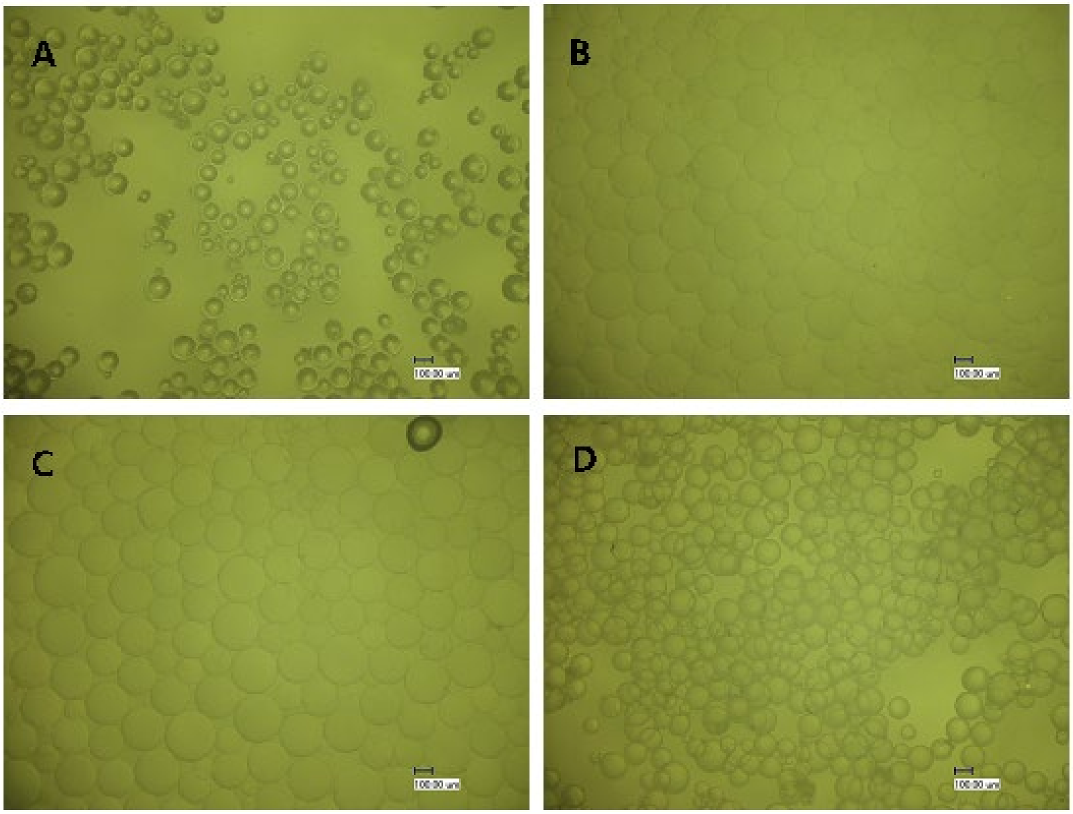

Figure S54. Brightfield images of crosslinked PD particles before (A) and after saturation in chloroform (B) benzene (C) and n-octane (D): The absorbencies (swelling ratios) for benzene, chloroform, and n-octane were 10.5, 16.3, and 3.4 times the dried particle weights, respectively. The absorption rates for benzene and chloroform were very fast (saturation in 7 seconds) compared to n-octane (saturation in 15 minutes). Magnification: 100X. Scale bar: 100 μm.

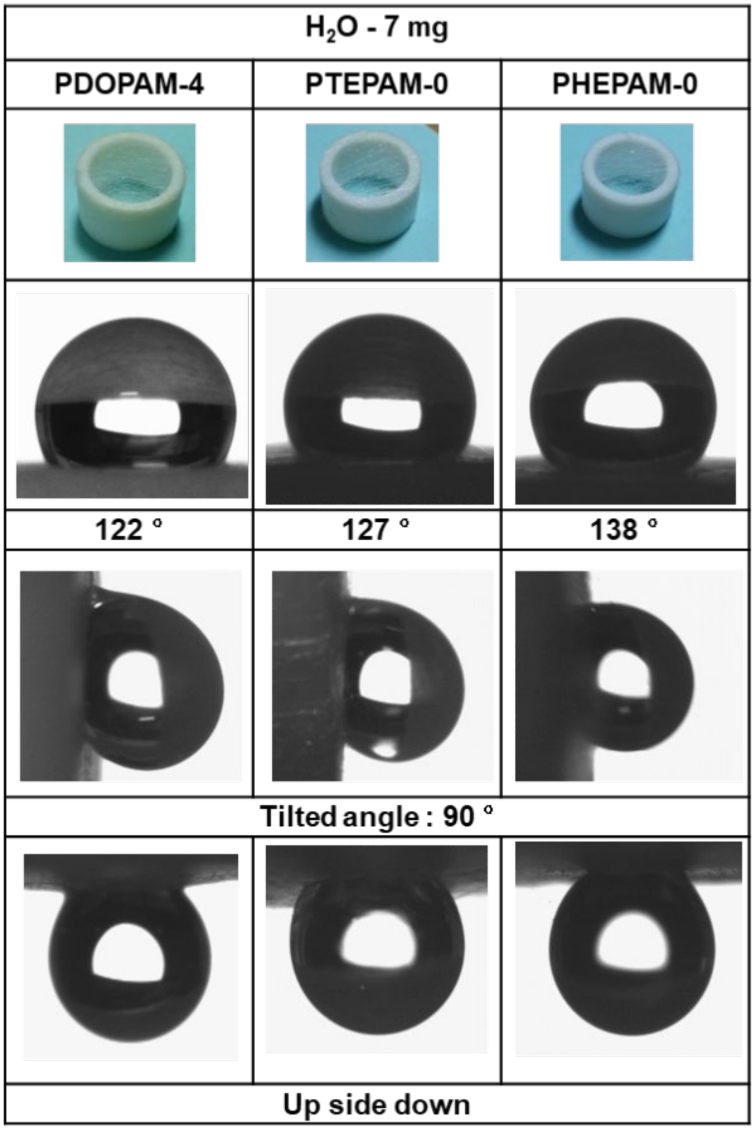

Figure S55. Contact angle of 7 mg water droplets on electrospun fibers of PD-4, PT-0, and PH-0.

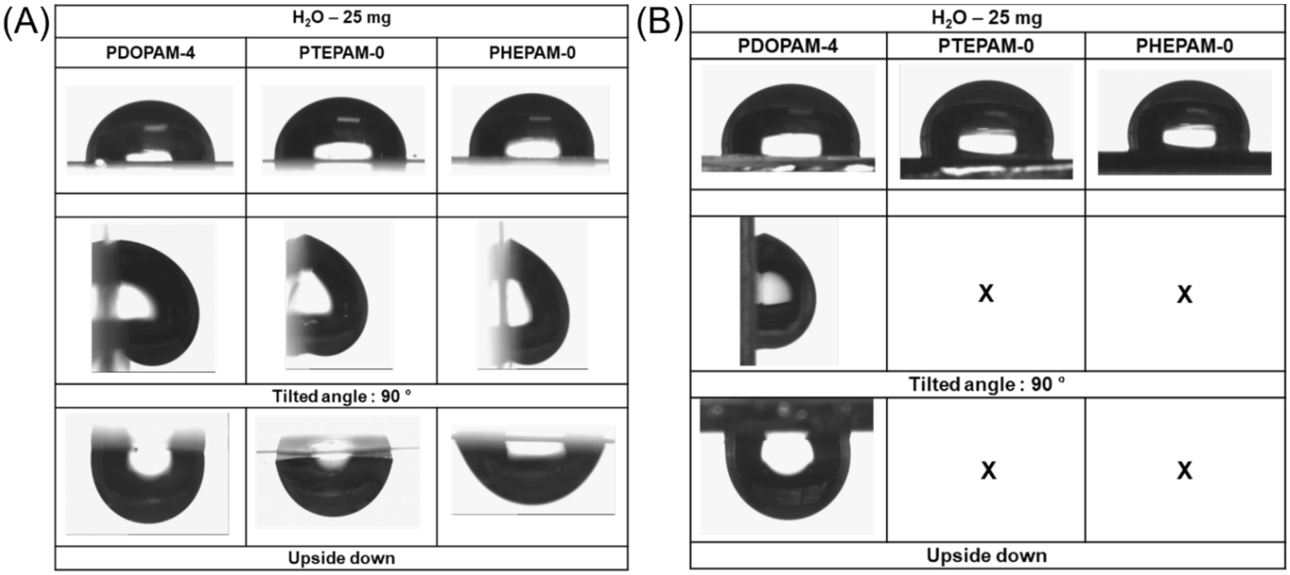

Figure S56. Contact angle of water droplets on (A) PD-4, PT-0, and PH-0 thick films (70±3 μm) and (B) thin films (25±2 nm) coated on a Si wafer. "X" indicates that the water droplets roll off the film surface.

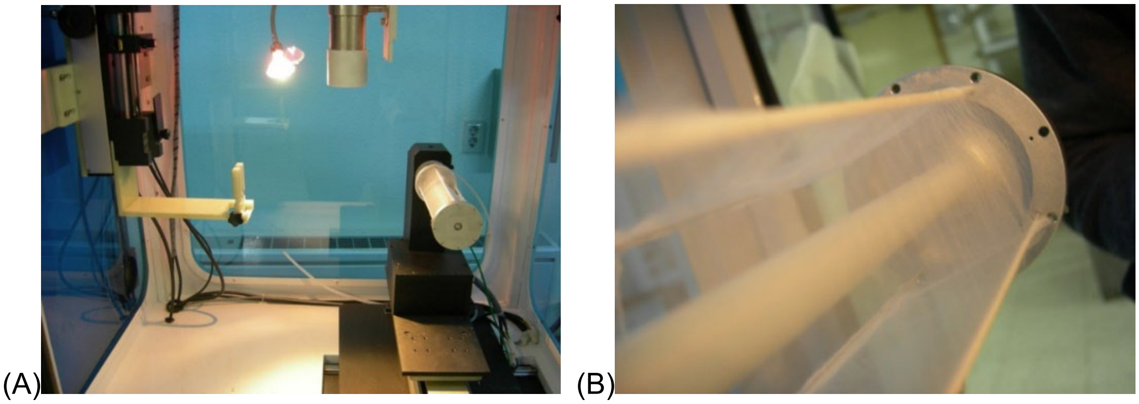

Figure S57. (A) Electrospinning device (NanoNC Model ESR-200RD, Korea) and (B) aligned fibers electrospun on a special aluminum collector consisting of 6 wires.

8. Tables S1 to S8

Table S1. Polymerization conditions and physical and chemical properties of poly(p-alkyl-N-phenyl-acrylamide) samples (PDOPAM, PTEPAM, PHEPAM) prepared by **MARRS** polymerization

Entry[1] #	Monomer[2] (g, wt%)	Initiator[3] (mg)	Solvent (mL)	Dielectric constant of solvent[4]	Temp (ºC) /Time (h)	M_n	Đ	Conv. (%)	Tacticity[5] (%) by ^{13}C-NMR		BET sample weight (g)	BET surface area ($m^2 g^{-1}$)	Mean pore diameter (nm)
									rr (syndio-)	mm (iso-)			
PD-0	3.0, 20	BPO (20.1)	Benzene (13.7)	2.28	80 / 48	116,900	2.84	86.4	[86.4] (80.1)	[13.6] (19.9)	0.1125	242.44	7.2628
PD-1	1.0, 20	BPO (6.7)	Benzene (4.6)	2.28	70 / 0.5	191,100	1.93	60.1	[87.6] (80.0)	[12.4] (20.0)	0.1134	267.99	6.9420
PD-2	3.0, 15	BPO (20.1)	Benzene (19.4)	2.28	70 / 0.5	134,400	1.88	54.5	[87.8] (81.1)	[12.2] (18.9)	0.099	232.39	7.4909
PD-3	1.5, 15	BPO (10.1)	Benzene (9.7)	2.28	70 / 1.0	143,400	1.94	54.7	[86.6] (79.7)	[13.4] (20.3)	0.1051	258.01	6.6865
PD-4	3.0, 10	BPO (20.1)	Benzene (30.8)	2.28	70 / 0.5	134,900	1.93	60.3	[82.5] (78.5)	[17.5] (21.5)	0.2089	264.14	6.7040
PD-5	1.5, 10	BPO (50.3)	Benzene (15.4)	2.28	70 / 0.5	80,900	2.08	13.4	-	-	-	-	-
PD-6	1.0, 5.0	BPO (6.7)	Benzene (21.6)	2.28	70 / 0.5	85,900	1.98	30.4	[82.5] (80.1)	[17.5] (19.9)	0.2276	255.13	6.8545
PD-7	2.0, 5.0	BPO (13.4)	Benzene (43.2)	2.28	70 / 2.0	88,600	2.02	47.0	-	-	-	-	-
PD-8[6]	1.5, 2.0	BPO (10.1)	Benzene (83.6)	2.28	70 / 72	56,600	1.80	32.0	[77.2] (74.7)	[22.8] (25.3)	0.1086	264.91	6.8793
PD-9	1.5, 10	AIBN (6.7)	Benzene (15.4)	2.28	70 / 0.5	152,800	2.15	71.4	[81.2] (75.1)	[18.8] (24.9)	-	-	-
PD-10	1.0, 10	AIBN (4.5)	Benzene (10.3)	2.28	60 / 0.5	170,200	2.80	65.0	[86.7] (80.2)	[13.3] (19.8)	-	-	-
PD-11	1.5, 15	AIBN (6.7)	Benzene (9.7)	2.28	60 / 2.0	169,800	2.68	87.2	[87.6] (80.5)	[12.4] (19.5)	0.1155	244.36	7.3956
PD-12	1.0, 20	BPO (6.7)	THF (4.5)	7.52	80 / 48	14,100	2.58	75.4	[88.9] (82.2)	[11.1] (17.8)	0.1114	238.33	6.7755
PD-13	1.0, 20	BPO (6.7)	THF (4.5)	7.52	70 / 2.0	19,300	1.66	41.4	[91.8] (87.6)	[8.2] (12.4)	0.1161	272.79	6.5504
PD-14	1.5, 10	BPO (10)	THF (15.1)	7.52	70 / 0.5	13,200	1.55	26.7	[85.0] (81.0)	[15.0] (19.0)	0.1921	270.45	5.8539
PD-15	1.0, 10	BPO (6.7)	THF (10.1)	7.52	65 / 3.0	9,100	1.64	59.6	[94.5] (84.5)	[5.5] (15.5)	0.1169	267.8	5.9746
PD-16[6]	1.0, 10	BPO (6.7)	Monoglyme (10.3)	7.30	70 / 0.5	56,700	1.74	15.2	[79.6] (84.5)	[20.4] (15.5)	0.1163	273.99	6.0980
PD-17	1.0, 10	BPO (6.7)	Dioxane (8.7)	2.21	70 / 0.5	92,200	2.04	44.0	[94.0] (90.7)	[6.0] (9.3)	0.1011	281.25	6.8636
PD-18	2.0, 10	BPO (13.4)	Dioxane (17.4)	2.21	70 / 1.0	101,800	1.99	63.1	[92.6] (85.4)	[7.4] (14.6)	-	-	-

Entry[1] #	Monomer[2] (g, wt%)	Initiator[3] (mg)	Solvent (mL)	Dielectric constant of solvent[4]	Temp (°C) /Time (h)	M_n	Đ	Conv. (%)	Tacticity[5] (%) by ^{13}C-NMR		BET sample weight (g)	BET surface area (m^2 g^{-1})	Mean pore diameter (nm)
									rr (syndio-)	mm (iso-)			
PD-18	2.0, 10	BPO<br>(13.4)	Dioxane<br>(17.4)	2.21	70 / 1.0	101,800	1.99	63.1	[92.6]<br>(85.4)	[7.4]<br>(14.6)	-	-	-
PD-19[6]	2.7, 20	BPO<br>(18.1)	Dioxane<br>(10.5)	2.21	70 / 2.0	104,600	2.24	73.7	[98.8]<br>(100)	[1.2]<br>(0)	0.119	261.35	6.6210
PD-20[7]	1.0, 10	BPO<br>(6.7)	DMF<br>(9.5)	38.25	70 / 0.5	55,500	1.74	13.6	[90.2]<br>(83.3)	[9.8]<br>(16.7)	-	-	-
PD-21[7]	1.0, 10	BPO<br>(6.7)	DMF<br>(9.5)	38.25	70 / 2.0	68,600	1.87	33.4	[84.1]<br>(79.6)	[15.9]<br>(20.4)	-	-	-
PD-22	1.0, 10	BPO<br>(6.7)	Toluene<br>(10.3)	2.38	70 / 0.5	112,000	1.8	21.8	[88.7]<br>(81.8)	[11.3]<br>(18.2)	-	-	-
PD-23	1.5, 10	AIBN<br>(6.7)	THF<br>(15.1)	7.52	70 / 0.5	13,500	1.61	66.7	[83.9]<br>(85.9)	[16.1]<br>(14.1)	0.2178	267.04	6.0620
PD-24	1.0, 10	AIBN<br>(4.5)	THF<br>(10.1)	7.58	60 / 2.0	14,100	1.51	72.1	[86.5]<br>(79.0)	[13.5]<br>(21.0)	-	-	-
PT-0	3.0, 10	BPO<br>(20.1)	Benzene<br>(30.8)	2.28	70 / 0.5	117,200	1.89	53.2	[86.6]<br>(80.6)	[13.4]<br>(19.4)	0.1028	257.46	7.6439
PT-1	3.0, 10	BPO<br>(20.1)	Benzene<br>(30.8)	2.28	70 / 1.0	167,000	2.2	64.9	[85.9]<br>(79.5)	[14.1]<br>(20.5)	0.2332	281.37	6.3091
PT-2[6]	2.0, 10	BPO<br>(20.1)	Benzene<br>(20.5)	2.28	70 / 0.5	96,700	2.14	53.3	[77.8]<br>(78.3)	[22.2]<br>(21.7)	0.0995	257.75	6.6312
PT-3	1.0, 7.0	BPO<br>(6.7)	Benzene<br>(15.1)	2.28	70 / 1.0	140,600	2.48	17.5	[84.7]<br>(80.3)	[15.3]<br>(19.7)	0.1233	284.03	6.2359
PT-4	1.5, 10	BPO<br>(30)	Benzene<br>(15.4)	2.28	70 / 0.5	131,700	2.62	18.0	[90.4]<br>(82.1)	[9.6]<br>(17.9)	0.1835	272.26	6.4967
PT-5	1.5, 10	AIBN<br>(6.8)	Benzene<br>(15.4)	2.28	70 / 0.5	115,000	2.41	72.0	[83.5]<br>(78.4)	[16.5]<br>(21.6)	-	-	-
PT-6[6]	2.0, 20	AIBN<br>(13.4)	Benzene<br>(9.1)	2.28	70 / 48	80,900	4.4	80.6	[55.2]<br>(53.8)	[44.8]<br>(46.2)	-	-	-
PT-7[6]	2.0, 20	BPO<br>(13.4)	THF<br>(9.0)	7.52	80 / 48	12,800	2.34	81.7	[77.6]<br>(78.0)	[22.4]<br>(22.0)	0.1093	235.63	6.7020
PT-8	1.5,10	BPO<br>(10)	THF<br>(15.1)	7.52	70 / 0.5	12,400	1.50	27.0	[93.2]<br>(90.6)	[6.8]<br>(9.4)	0.3225	268.23	6.1231
PT-9	1.5,10	AIBN<br>(6.8)	THF<br>(15.1)	7.52	70 / 0.5	12,400	1.55	67.2	[86.4]<br>(83.5)	[13.6]<br>(16.5)	-	-	-
PH-0	1.5, 10	BPO<br>(10)	Benzene<br>(15.4)	2.28	70 / 0.5	128,500	2.05	54.7	[82.3]<br>(78.2)	[17.7]<br>(21.8)	0.2246	238.84	7.0742
PH-1[6]	1.0, 10	AIBN<br>(4.5)	Benzene<br>(10.3)	2.28	65 / 0.5	176,900	2.45	67.0	[79.4]<br>(77.3)	[20.6]<br>(22.7)	-	-	-
PH-2	1.5, 10	BPO<br>(10)	THF<br>(15.1)	7.52	70 / 0.5	15,000	1.46	30.1	[85.3]<br>(79.4)	[14.7]<br>(20.6)	0.2352	226.28	7.7797

[1]PD: PDOPAM; PT: PTEPAM; PH: PHEPAM. [2]Monomer concentration (wt%) in solvent. [3]BPO: benzoyl peroxide; AIBN: 2,2-azobisisobutyronitrile. [4]The dielectric constant was measured at 20°C except for toluene and 1,4-dioxane, which were measured at 25°C, and reported by the Division of Organic Chemistry of the American Chemical Society. [5]Triad tacticity, the ratio of [syndiotactic] to [isotactic], was measured by quantifying the relative proportions of racemic (rr) to meso (mm) triads in ^{13}C NMR (Bruker Avance III HD 500) spectra. The quantitative ratio of rr to mm was calculated using commercially available [Origin] or (Igor) software. [6]To measure the tacticity, THF-d8 was used as a solvent for ^{13}C NMR (500 MHz), except that $CDCl_3$ was used in PD-8, PD-16, PD-19, PT-2, PT-6, PT-7, and PH-1. [7]Unlike other solvents, dimethyl formamide (DMF) precipitated the polymers during polymerization. (-): not measured.

Table S2. Characteristic carbon peak positions (ppm) in the ^{13}C CP MAS solid state NMR spectra of DOPAM monomer and mesoporous APAA polymers and in the ^{13}C NMR spectra of their THF-d_8 solutions

Sample	Carbon	Peaks						
DOPAM monomer	Carbonyl	not observed at 25°C, but 164.6 at 100 and 110°C						
	Aromatic[1] & Vinyl[1]	139.0 ($\mathbf{C_a}$), 136.4 ($\mathbf{C_b}$), 132.5 ($\mathbf{C_c}$), 128.5 ($\mathbf{C_d}$), 125.8 ($\mathbf{C_e}$), 121.3 ($\mathbf{C_f}$)						
	Dodecyl group -$(CH_2)_{11}CH_3$	**Peak positions (ppm) at different temperatures (°K)**						
		**298**	**323**	**343**	**353**	**373**	**383**	**298**
	CH_2 (C_1, benzyl)	37.5	37.0	36.5	36.5	35.5	35.5	37.5
	CH_2 (C_2)	35.5	35.5	35.0	34.7	32.0	32.0	35.5
	CH_2 (C_3)	35.5	35.5	35.0	34.7	31.3	31.3	35.5
	CH_2 (C_4~C_{10})	33.8	33.8	33.4	33.0	29.6	29.6	33.8
	CH_2 (C_{11})	25.4	25.2	24.9	24.9	22.6	22.6	25.4
	CH_3 (C_{12})	15.9	15.9	15.9	15.9	14.0	14.0	15.9
PD-4 (PT-0)[2]	Carbonyl PD (PT)	174.1 (173.2) at 25~110°C, insensitive to temp.						
	Aromatic[1] PD (PT)	136.2 (135.9), 127.8 (127.3), 120.5 (119.5), insensitive to temp.						
		**Peak positions (ppm) at different temperatures (°K)**						
		**298**	**323**	**343**	**353**	**373**	**383**	**298**
	α-carbon[3] PD (PT)	42.4 (42.2)	-	42.4 (42.2)	42.4 (42.2)	42.4 (42.2)	42.4 (42.2)	42.4 (42.2)
	β-carbon[3] PD (PT)	35.73 (35.3)	-	35.73 (35.3)	35.73 (35.3)	35.73 (35.3)	35.73 (35.3)	35.73 (35.3)
	Alkyl group							
	CH_2 (C_1, benzyl) PD (PT)	32.42 (31.40)	-	32.24 (31.23)	32.24 (31.23)	32.10 (31.13)	32.00 (31.03)	32.42 (31.40)
	CH_2 (PD, C_2~C_{10}) (PT, C_2~C_{12})	30.38 (29.36)	-	30.05 (29.02)	30.05 (29.02)	29.90 (28.92)	29.85 (28.82)	30.36 (29.37)
	CH_2 (PD, C_{11}) (PT, C_{13})	23.12 (22.18)	-	22.91 (21.88)	22.91 (21.88)	22.80 (21.78)	22.71 (21.70)	23.12 (22.17)
	PD, CH_3 (C_{12}) (PT, C_{14})	14.22 (13.35)	-	14.05 (13.17)	14.05 (13.17)	13.95 (13.07)	13.82 (12.95)	14.22 (13.35)
PT-6[4]	Carbonyl	172.39 at 25~110°C, weak and broad peak, insensitive to temp.						
	Aromatic[1]	141.15~112.61, medium and broad peak						
	α-carbon	43.72, weak and broad shoulder peak						
	β-carbon	38.38, weak shoulder peak						
	Teradecyl group	36.66~24.81, broad and strong peak*						
PD-4 (PT-0) in THF-d_8[5]	Carbonyl PD (PT)	173.0 (173.1) at room temp., weak and broad peak						
	Aromatic[1] PD (PT)	137.1 (137.2), 127.9 (128.0), 120.2 (119.9), weak but sharp peaks						
	α-carbon[3] PD (PT)	syndiotactic 43.35 (43.33); isotactic 42.51 (42.46)						
	β-carbon[3] PD (PT)	35.5 (35.2), weak and sharp peak						
	Alkyl group							
	Benzyl CH_2: C_1 PD (PT)	31.53 very strong and sharp peak (31.36)						
	PD, C_2~C_{10} CH_2 (PT, C_2~C_{12} CH_2)	29.23 and 28.84, strong and sharp two peaks (29.10)						
	PD, C_{11} CH_2 (PT, C_{13} CH_2)	22.30 very strong and sharp peak (21.82)						
	PD, C_{12} CH_3 (PT, C_{14} CH_3)	13.46, very strong and sharp peak (13.18)						

[1]The characteristic carbons in the aromatic group are split into four peaks ($\mathbf{C_a}$, $\mathbf{C_b}$, $\mathbf{C_c}$, $\mathbf{C_e}$) in DOPAM monomer (**Figure S7**) and three peaks ($\mathbf{C_a}$, $\mathbf{C_b}$, $\mathbf{C_c}$) in PD and PT polymers (**Figures S8 and S9**). The α and β carbons of the vinyl group of DOPAM are separated into two distinct peaks (**$\mathbf{C_d}$ and $\mathbf{C_f}$**). [2]PD-4 (M_n=139,000; PDI=19.3) and

PT-0 (M_n=117,200; PDI=1.89) are homopolymers prepared from DOPAM with dodecyl group [-$(CH_2)_{11}CH_3$] and TEPAM with tetradecyl group [-$(CH_2)_{13}CH_3$], respectively. [3]α and β-carbons in the polymer backbone are represented by a weak and broad peak and a medium sharp peak, respectively. [4]PT-6 (M_n=80,900; PDI=4.40) has no tacticity ([rr/mm] = [Origin, 55.2/44.8]) in the main chain. As a result, unlike PD-4 and PT-0 polymers, the alpha carbon in the backbone chain and all the characteristic carbons in the tetradecyl group overlap to form a strong and broad peak, as shown in **Figure S10**. [5]The characteristic carbon peaks in the ^{13}C NMR spectrum of a representative stereoregular PD-4 polymer using THF-d_8 (or $CDCl_3$) solvent are shown in **Figure S11**, where individual characteristic carbon peaks of the carbonyl, benzene, and long alkyl groups in PD-4 are present in the same position as those in PD-8, PD-14, or PD-17. However, the alpha carbon (-CH-) of each polymer splits into syndiotactic (racemic, rr) and isotactic (meso, mm) triads between 46 and 41 ppm, as shown in **Figure S12**. The triad tacticity of representative PT and PH polymers is also shown in **Figures S13 and S14**.

Table S3. Interdomain spacing of lamellar and hexagonal cylindrical nanostructures and shoulder peak of novel mesoporous APAA polymers

Polymer	Characterization Method	First Nanostructure Peak (Å)		**Shoulder Peak** (Å) (225℃)
		**Lamella** (25℃)	**Cylinder** (225℃)	
**PD-14**	SAXS#	33.6	37.2	30.5
	MM simulation*	35.2	37.2	X
**PT-8**	SAXS	34.0	37.2	30.4
	MM simulation	36.0	38.2	X
**PH-2**	SAXS	34.7	38.1	32.6
	MM simulation	38.4	40.5	X

#Obtained from **Figure 1d** for PD-14, **Figure. S19** for PT-8 and **Figure S20** for PH-2, respectively; *Calculated by a molecular mechanics (MM) simulation method using NAMD based on OPLS force field.

Table S4. Thermal stability of mesoporous APAA polymers with temperature

Polymer	Weight loss temperature (°C)			at 480°C
	3%	5%	10%	
**PD-4**	321	338	360	5.7 %
**PT-0**	339	349	360	10.8 %
**PH-0**	300	325	354	6.7 %

Table S5. Variation of enthalpy and T_C as a function of annealing temperature of PD-14

Cycle	T_A	First Enthalpy (J/g)	Second Enthalpy (J/g)	T_C^1 (°C)	T_C^2 (°C)
Down	230	6.251	7.899	235.81	248.67
	225	3.741	9.511	231.38	246.07
	220 ↓	2.54	10.07	225.7	243
Up	220	2.27	8.17	225.16	242.5
	225	5.277	4.451	230.80	245.39
	230 ↓	8.176	0.5986	234.85	249.57

Table S6. Preparation conditions of mesoporous APAA films and their quantum yields

Entry #	APAA polymers			Thickness (µm)	Preparation condition of polymer films[a]		**Quantum Yield[b] (±1 %)**
	M_n	Đ	Syndiotactic (%)		Solvent	Polymer concentration	
PD-0-F1	116,900	2.84	86.4	50	CF	4%	**11.1**
PD-4-F1	134,900	1.93	82.5	50	CF	4%	**11.7**
PD-4-F2				60	BZ	4%	**12.1**
PD-4-F3				130	CF	7%	**12.1**
PD-4-F4				220	BZ	7%	**13.7**
PT-0-F1	117,200	1.89	86.6	50	Bz	4%	**10.3**
PT-1-F1	167,000	2.20	85.9	40	CF	4%	**11.0**
PT-1-F2				130	CF	7%	**14.9**
PT-1-F3				310[c]	CF	10%	**8.3**
PT-1-F4				340			**17.3**
PT-1-F5				160	BZ	8%	**16.4**
PT-6-F1	80900	2.28	55.2	45	CF	5%	**7.5**
PT-6-F2				140	BZ	9%	**8.3**

[a]Polymer films were cast onto glass plates using APAA polymer solutions in chloroform (CF) or benzene (BZ). [b]As-cast films were annealed at 150°C for 1 h prior to quantum yield measurement. [c]The film was annealed at 200°C for 1 h prior to quantum yield measurement and the resulting transparent film turned pale yellow due to traces of thermal damage.

Table S7. Fluorescence lifetime (decay time) of APAA polymer films*

Sample	Excitation Wavelength (nm)	Emission Wavelength (nm)	τ_1	f_1	τ_2	f_2	χ^2
PD-4 50 µm	374	401-552	1.665	0.655	5.374	0.345	1.317
PD-4 70 µm	374	398-549	1.739	0.710	5.501	0.290	1.495
PT-3 50 µm	374	404-550	1.520	0.745	6.734	0.255	1.597
PH-0 50 µm	374	399-550	1.319	0.624	4.824	0.376	1.070

*τ_1 and τ_2 are lifetimes (ns), f_1 and f_2 are fractional intensities, and χ^2 is the reduced chi-square.

Table S8. Tensile properties of APAA polymer films

Sample*	Thickness (µm)	Modulus (MPa)	Ultimate Stress (MPa)	Stress at break (MPa)	Strain at break (%)
**PD-0**	60	473.9	22.5	13.08	9.7
**PT-6**	62	359.4	20.8	12.10	14.1

9. References

[1] T. D. Bennett, F.-X. Coudert, S. L. James, A. I. Cooper, The changing state of porous materials. *Nat. Mater.* **2021,** *20*, 1179-1187. doi:10.1038/s41563-021-00957-w

[2] A. G. Slater, A. I. Cooper, Function-led design of new porous materials. *Science* **2015,** *348*, aaa8075. doi:10.1126/science.aaa8075

[3] T. Xue, T. He, L. Peng, O. A. Syzgantseva, R. Li, C. Liu, D. T. Sun, G. Xu, R. Qiu, Y. Wang, S. Yang, J. Li, J.-R. Li, W. L. Queen, A customized MOF-polymer composite for rapid gold extraction from water matrices. *Sci. Adv.* **2023,** *9*, eadg4923. doi:10.1126/sciadv.adg4923

[4] S. Fajal, S. Dutta, S. K. Ghosh, Porous organic polymers (POPs) for environmental remediation. *Mater. Horiz*. **2023,** *10*, 4083-4138. doi:10.1039/D3MH00672G

[5] A. Pal, S. Suresh, A. Khan, L. H. Kuo, L. T. Chi, A. Ganguly, C.-Y. Kao, M. K. Sharma, T.-S. A. Wang, D.-Y. Kang, Z.-H. Lin, Metal-organic frameworks as thermocatalysts for hydrogen peroxide generation and environmental antibacterial applications. *Sci. Adv.* **2025,** *11*, eads4711. DOI: 10.1126/sciadv.ads4711

[6] J. E. Mondloch, M. J. Katz, W. C. Isley III, P. Ghosh, P. Liao, W. Bury, G. W. Wagner, M. G. Hall, J. B. DeCoste, G. W. Peterson, R. Q. Snurr, C. J. Cramer, J. T. Hupp, O. K. Farha, Destruction of chemical warfare agents using metal–organic frameworks. *Nat. Mater.* **2015,** *14*, 512-516. doi:10.1038/nmat4238

[7] R. Zhuang, X. Zhang, C. Qu, X. Xu, J. Yang, Q. Ye, Z. Liu, S. Kaskel, F. Xu, H. Wang, Fluorinated porous frameworks enable robust anode-less sodium metal batteries. *Sci. Adv.* **2023,** *9*, eadh8060. doi:10.1126/sciadv.adh8060

[8] A. H. Alawadhi, S. Chheda, G. D. Stroscio, Z. Rong, D. Kurandina, H. L. Nguyen, N. Rampal, Z. Zheng, L. Gagliardi, O. M. Yaghi, Harvesting water from air with high-capacity, stable furan-based metal–organic frameworks. *J. Am. Chem. Soc.* **2024,** *146*, 2160-2166. doi:10.1021/jacs.3c11947

[9] J. A. Finbloom, C. Huynh, X. Huang, T. A. Desai, Bioinspired nanotopographical design of drug delivery systems. *Nat. Rev. Bioeng*. **2023,** *1*, 139-152. doi:10.1038/s44222-022-00010-8

[10] J. Yang, D. Dai, X. Zhang, L. Teng, L. Ma, Y.-W. Yang, Multifunctional metal-organic framework (MOF)-based nanoplatforms for cancer therapy: From single to combination therapy. *Theranostics*, **2023,** *13*, 295-323. doi:10.7150/thno.80687

[11] D. Gang, Z. Uddin Ahmad, Q. Lian, L. Yao, M. E. Zappi, A review of adsorptive remediation of environmental pollutants from aqueous phase by ordered mesoporous carbon. *Chem. Eng. J.* **2021,** *403*, 126286. doi:10.1016/j.cej.2020.126286

[12] D.-H. Choi, R. Ryoo, Template synthesis of ordered mesoporous organic polymeric materials using hydrophobic silylated KIT-6 mesoporous silica. *J. Mater. Chem.* **2010,** *20*, 5544-5550. doi:10.1039/C0JM00671H

[13] J. G. Croissant, Y. Fatieiev, A. Almalik, N. M. Khashab, Mesoporous silica and organosilica nanoparticles: Physical chemistry, biosafety, delivery strategies, and biomedical applications. *Adv. Healthc. Mater*. **2018,** *7*, 1700831. doi:10.1002/adhm.201700831

[14] M. Robertson, M. M. Zagho, S. Nazarenko, Z. Qiang, Mesoporous carbons from self-assembled polymers. *J. Polym. Sci*. **2022,** *60*, 2015-2042. doi:10.1002/pol.20220122

[15] M. Seo, M. A. Hillmyer, Reticulated Nanoporous Polymers by Controlled Polymerization-Induced Microphase Separation. *Science* **2012,** *336*, 1422-1425. doi:10.1126/science.1221383

[16] Z. Zhang, Z. Liu, C. Xue, H. Chen, X. Han, Y. Ren, Amorphous porous organic polymers containing main group elements. *Comms. Chem.* **2023,** *6*, 271. doi:10.1038/s42004-023-01063-5

[17] X. Han, Z. Zhou, K. Wang, Z. Zheng, S. E. Neumann, H. Zhang, T. Ma, O. M. Yaghi, Crystalline polyphenylene covalent organic frameworks. *J. Am. Chem. Soc*. **2024,** *146*, 89-94. doi:10.1021/jacs.3c11688

[18] G. Cai, P. Yan, L. Zhang, H.-C. Zhou, H.-L. Jiang, Metal−organic framework-based hierarchically porous materials: Synthesis and applications. *Chem. Rev.* **2021,** *121*, 12278-12326. doi:10.1021/acs.chemrev.1c00243
[19] M. Kalaj, K. C. Bentz, S. Ayala, Jr., J. M. Palomba, K. S. Barcus, Y. Katayama, S. M. Cohen, MOF-polymer hybrid materials: From simple composites to tailored architectures. *Chem. Rev.* **2020,** *120*, 8267-8302. doi:10.1021/acs.chemrev.9b00575
[20] X. Song, Y. Wang, C. Wang, D. Wang, G. Zhuang, K. O. Kirlikovali, P. Li, O. K. Farha, Design rules of hydrogen-bonded organic frameworks with high chemical and thermal stabilities. *J. Am. Chem. Soc*. **2022,** *144*, 10663-10687. doi:10.1021/jacs.2c02598
[21] M.S. Silverstein, PolyHIPEs: Recent advances in emulsion-templated porous polymers. *Prog. Polym. Sci.* **2014,** *39*, 199-234. doi:10.1016/j.progpolymsci.2013.07.003
[22] A. Zenati, Y.-K. Han, Synthesis and properties of azo-based ABC triblock copolymers owning interaction and composition parameters that influence their phase behaviors. *Macromolecules* **2018,** *51*, 101-114. doi:10.1021/acs.macromol.7b02012
[23] I. J. Byun, J. H. Lee, K. U. Jeong, Y. K. Han, Synthesis of high χ block copolymers with LC moieties and PMMA segments using RAFT polymerization, and their nanostructure morphologies. *Polymer* **2018,** *145*, 184-193. doi:10.1016/j.polymer.2018.04.072
[24] J. G. Lee, Y. S. Jung, S. H. Han, K. M. Kim, Y. K. Han, Long-range ordered self-assembly of novel acrylamide-based diblock copolymers for nanolithography and metallic nanostructure fabrication. *Adv. Mater.* **2014,** *26*, 2894-2900. doi:10.1002/adma.201305186
[25] Y. Isobe, D. Fujioka, S. Habaue, Y. Okamoto, Efficient Lewis acid-catalyzed stereocontrolled radical polymerization of acrylamides. *J. Am. Chem. Soc.* **2001,** *123*, 7180-7181. doi:10.1021/ja015888l
[26] T. Kitayama, W. Shibuya, K.-i. Katsukawa, Synthesis of highly isotactic poly(N-isopropylacrylamide) by anionic polymerization of a protected monomer. *Polym. J.* **2002,** *34*, 405-409. doi:10.1295/polymj.34.405
[27] W. Liu, T. Nakano, Y. Okamoto, Stereocontrol in radical polymerization of N,N-dimethylacrylamide and N,N-diphenylacrylamide and thermal properties of syndiotactic poly(methyl acrylate)s derived from the obtained polymers. *Polym. J*. **2000,** *32*, 771-777. doi:10.1295/polymj.32.771
[28] J. Mei, N. L. C. Leung, R. T. K. Kwok, J. W. Y. Lam, B. Z. Tang, Aggregation-induced emission: Together we shine, united we soar. *Chem. Rev*. **2015,** *115*, 11718-11940. doi:10.1021/acs.chemrev.5b00263
[29] N. Ishihara, M. Kuramoto, M. Uoi, Stereospecific polymerization of styrene giving the syndiotactic polymer. *Macromolecules* **1988,** *21*, 3356-3360. doi:10.1021/ma00190a003
[30] R. Pó, N. Cardi, Synthesis of syndiotactic polystyrene: Reaction mechanisms and catalysis. *Prog. Polym. Sci.* **1996,** *21*, 47-88. https://doi.org/10.1016/0079-6700(95)00016-X
[31] E. M. Woo, Y. S. Sun, C. P. Yang, Polymorphism, thermal behavior, and crystal stability in syndiotactic polystyrene vs. its miscible blends. *Prog. Polym. Sci.* **2001,** *26*, 945-983. https://doi.org/10.1016/S0079-6700(01)00010-7
[32] L. S. Baugh, D. N. Schulz, Discovery of Syndiotactic Polystyrene: Its Synthesis and Impact. *Macromolecules* **2020,** *53*, 3627-3631. https://dx.doi.org/10.1021/acs.macromol.0c00350
[33] J. C. Phillps, R. Braun, W. Wang, J. Gumbart, E. Tajkhorshid, E. Villa, C. Chipot, R. D. Skeel, L. Kale, K. Schulten, Scalable molecular dynamics with NAMD. *J. Comput, Chem.* **2005,** *26*, 1781-1802. doi:10.1002/jcc.20289
[34] T. Komamura, K. Okuhara, S. Horiuchi, Y. Nabae, T. Hayakawa, Fabrication of well-ordered mesoporous polyimide films by a soft-template method. *ACS Appl. Polym. Mater*. **2019,** *1*, 1209-1219. doi:10.1021/acsapm.9b00211

[35] J. X. Shi, H. Yang, S. Y. Xing, H. Zhang, Molecular dynamics simulation of the fold of alkyl groups with different lengths when N-hexane molecules forming ordered structure on their functionalized graphene. *Surf. Sci*. **2022,** *716*, 121965. doi:10.1016/j.susc.2021.121965
[36] A. Alsbaiee, B. J. Smith, L. Xiao, Y. Ling, D. E. Helbling, W. R. Dichtel, Rapid removal of organic micropollutants from water by a porous β-cyclodextrin polymer. *Nature* **2016,** *529*, 190-194. doi:10.1038/nature16185
[37] D. J. Skrovanek, S. E. Howe, P. C. Painter, M. M. Coleman, Hydrogen bonding in polymers: Infrared temperature studies of an amorphous polyamide. *Macromolecules* **1985,** *18*, 1676-1683. doi: 10.1021/ma00151a006
[38] N. S. Myshakina, Z. Ahmed, S. A. Asher, Dependence of amide vibrations on hydrogen bonding. *J. Phys. Chem. B* **2008,** *112*, 11873-11877. doi:10.1021/jp8057355
[39] M. Miyazaki, J. Saikawa, H. Ishizuki, T. Taira, M. Fujii, Isomer selective infrared spectroscopy of supersonically cooled cis- and trans-N-phenylamides in the region from the amide band to NH stretching vibration. *Phys. Chem. Chem. Phys*. **2009,** *11*, 6098-6106. doi:10.1039/B822310F
[40] Y. M. Jung, I. Noda, New approaches to generalized two-dimensional correlation spectroscopy and its applications. *Appl. Spectrosc. Rev.* **2006,** *41*, 515-547. doi:10.1080/05704920600845868
[41] L. Guo, H. Sato, T. Hashimoto, Y. Ozaki, Thermally induced exchanges of hydrogen bonding interactions and their effects on phase structures of poly(3-hydroxybutyrate) and poly(4-vinylphenol) blends. *Macromolecules* **2011,** *44*, 2229-2239. doi:10.1021/ma102601p
[42] H. Zhang, Z. Zhao, P. R. McGonigal, R. Ye, S. Liu, J. W.Y. Lam, R. T.K. Kwok, W. Z. Yuan, J. Xie, A. L. Rogach, B. Z. Tang, Clusterization-triggered emission: Uncommon iuminescence from common materials. *Materials Today*, **2020,** *32*, 275-292. doi:10.1016/j.mattod.2019.08.010
[43] P. Shen, Z. Zhuang, X.-F. Jiang, J. Li, S. Yao, Z. Zhao, B. Z. Tang, Through-space conjugation: An effective strategy for stabilizing intramolecular charge-transfer states. *J. Phys. Chem. Lett.* **2019,** *10*, 2648-2656. doi:10.1021/acs.jpclett.9b01040
[44] J.-J. Yan, Z.-K. Wang, X.-S. Lin, C.-Y. Hong, H.-J. Liang, C.-Y. Pan, Y.-Z. You, Polymerizing nonfluorescent monomers without incorporating any fluorescent agent produces strong fluorescent polymers. *Adv. Mater*. **2012,** *24*, 5617-5624. doi:10.1002/adma.201202201
[45] X. Chen, Z. He, F. Kausar, G. Chen, Y. Zhang, W. Z. Yuan, Aggregation-Induced dual emission and unusual luminescence beyond excimer emission of poly(ethylene terephthalate). *Macromolecules* **2018,** *51*, 9035-9042. doi:10.1021/acs.macromol.8b01743
[46] D. Neher, Polyfluorene Homopolymers: Conjugated Liquid-Crystalline Polymers for Bright Blue Emission and Polarized Electroluminescence. *Macromol. Rapid Commun.* **2001,** *22*, 1365-1385. doi:10.1002/1521-3927(20011101)22:17<1365::AID-MARC1365>3.0.CO;2-B
[47] Q. Kuang, X. Hou, C. Du, X. Wang, D. Gao, Recent advances in the anti-counterfeiting applications of long persistent phosphors. *Phys. Chem. Chem. Phys*. **2023,** *25*, 17759-17768. doi:10.1039/D3CP01818K
[48] J. Xue, T. Wu, Y. Dai, Y. Xia, Electrospinning and electrospun nanofibers: Methods, materials, and applications. *Chem. Rev.* **2019,** *119*, 5298-5415. doi:10.1021/acs.chemrev.8b00593
[49] C. L. Casper, J. S. Stephens, N. G. Tassi, D. B. Chase, J. F. Rabolt, Controlling surface morphology of electrospun polystyrene fibers: Effect of humidity and molecular weight in the electrospinning process. *Macromolecules* **2004,** *37*, 573-578. doi:10.1021/ma0351975
[50] X. Xu, J.-F. Zhang, Y. Fan, Fabrication of cross-linked polyethyleneimine microfibers by reactive electrospinning with in situ photo-cross-linking by UV radiation. *Biomacromolecules* **2010,** *11*, 2283-2289. doi:10.1021/bm1003509
[51] L. Zhu, D. Shen, K. H. Luo, A critical review on VOCs adsorption by different porous materials: Species, mechanisms and modification methods. *J. Hazard. Mater.* **2020,** *389*, 122102. doi:10.1016/j.jhazmat.2020.122102

[52] T. Ono, T. Sugimoto, S. Shinkai, K. Sada, Lipophilic polyelectrolyte gels as super-absorbent polymers for nonpolar organic solvents. *Nat. Mater.* **2007,** *6*, 429-433. doi:10.1038/nmat1904
[53] T. Rattanaumpa, W. Naowanon, S. Amnuaypanich, S. Amnuaypanich, Polydimethylsiloxane sponges Incorporated with mesoporous silica nanoparticles (PDMS/H-MSNs) and their selective solvent absorptions. *Ind. Eng. Chem. Res.* **2019,** *58*, 21142-21154. doi:10.1021/acs.iecr.9b02946
[54] L. Feng, Y. Zhang, J. Xi, Y. Zhu, N. Wang, F. Xia, L. Jiang, Petal Effect: A superhydrophobic state with high adhesive force. *Langmuir* **2008,** *24*, 4114-4119. doi:10.1021/la703821h
[55] S. Parvate, P. Dixit, S. Chattopadhyay, Superhydrophobic surfaces: Insights from theory and experiment. *J. Phys. Chem. B* **2020,** *124*, 1323-1360. doi:10.1021/acs.jpcb.9b08567
[56] K. Deng, H. Feng, Y. Zhang, D. Liu, Q. Li, Ampere-level membrane-less water electrolysis enabled by rose-petal-effect-mimetic interface. *Joule* **2023,** *7*, 1852-1866. doi:10.1016/j.joule.2023.06.010
[57] C. Belbéoch, J. Lejeune, P. Vroman, F. Salaün, Silkworm and spider silk electrospinning: A review. *Environ. Chem. Lett.* **2021,** *19*, 1737-1763. doi:10.1007/s10311-020-01147-x
[58] S. W. Cranford, A. Tarakanova, N. M. Pugno, M. J. Buehler, Nonlinear material behaviour of spider silk yields robust webs. *Nature* **2012,** *482*, 72-76. doi:10.1038/nature10739
[59] S. Keten, Z. Xu, B. Ihle, M. J. Buehler, Nanoconfinement controls stiffness, strength and mechanical toughness of β-sheet crystals in silk. *Nat. Mater*. **2010,** *9*, 359-367. doi:10.1038/nmat2704
[60] Y. Zheng, H. Bai, Z. Huang, X. Tian, F.-Q. Nie, Y. Zhao, J. Zhai, L. Jiang, Directional water collection on wetted spider silk. *Nature* **2010,** *463*, 640-643. doi:10.1038/nature08729
[61] G. Ordian, *Principles of Polymerization*. 4th ed., Wiley: **2004**, pp. 633-637. doi:10.1002/047147875X.ch8
[62] Q. T. Pham, R. Petiaud, H. Waton, M.-F. Llauro-Darricades, *Proton and Carbon NMR Spectra of Polymers*. 5th ed., Wiley: **2002**, pp. 2–3 (ISBN: 10. 0470850752).
[63] B. G. M. Vandeginste, D. L. Massart, L. M. C. Buydens, S. De Jong, P. J. Lewi, J. Smeyers-Verbeke, "Analysis of Measurement Tables" In *Handbook of Chemometrics and Qualimetrics: Part B,* Elsevier: **1998**, Vol. 20, pp 87-160. doi:10.1016/S0922-3487(98)80041-5
[64] S. D. Brown, S. T. Sum, F. Despagne, B. K. Lavine, Chemometrics. *Anal. Chem.* **1996,** *68*, 21-62. doi:10.1021/a1960005x
[65] W. L. Jorgensen, D. S. Maxwell, J. Tirado-Rives, Development and testing of the OPLS all-atom force field on conformational energetics and properties of organic liquids. *J. Am. Chem. Soc.* **1996,** *118*, 11225-11236. doi:10.1021/ja9621760
[66] M. Thommes, K. Kaneko, A. V. Neimark, J. P. Olivier, F. Rodriguez-Reinoso, J. Rouquerol, K. S. W. Sing, Physisorption of gases, with special reference to the evaluation of surface area and pore size distribution (IUPAC Technical Report). *Pure Appl. Chem.* **2015,** *87* (9-10), 1051-1069. doi:10.1515/pac-2014-1117
[67] A. McClellan, H. F. Harnsberger, Cross-sectional areas of molecules adsorbed on solid surfaces. *J. Colloid Interface Sci*. **1967,** *23*, 577-599. doi:10.1016/0021-9797(67)90204-4
[68] A. Ahmed, S. Seth, J. Purewal, A. G. Wong-Foy, M. Veenstra, A. J. Matzger, D. J. Siegel, Exceptional hydrogen storage achieved by screening nearly half a million metal-organic frameworks. *Nat. Commun*. **2019,** *10*, 1568. doi:10.1038/s41467-019-09365-w
[69] J. M. Scherer, Principles of quantum dot photophysics and applications to luminescent downshifting. Massachusetts Institute of Technology, **2016**, pp. 45–48.
